# Identification and characterization of lncRNA-stemness-immune regulatory patterns

**DOI:** 10.1093/bib/bbag287

**Published:** 2026-06-04

**Authors:** Zhipeng Qian, Jiaqi Yin, Chunlong Zhang, Guohua Wang, Chunyu Wang, Yang Li, Yuming Zhao

**Affiliations:** College of Life Sciences, Northeast Forestry University, No. 26 Hexing Road, Xiangfang District, Harbin, Heilongjiang 150040, China; College of Computer and Control Engineering, Northeast Forestry University, No. 26 Hexing Road, Xiangfang District, Harbin, Heilongjiang 150040, China; College of Computer and Control Engineering, Northeast Forestry University, No. 26 Hexing Road, Xiangfang District, Harbin, Heilongjiang 150040, China; College of Computer and Control Engineering, Northeast Forestry University, No. 26 Hexing Road, Xiangfang District, Harbin, Heilongjiang 150040, China; School of Computer Science and Technology, Harbin Institute of Technology, No. 92 Xidazhi Street, Nangang District, Harbin, Heilongjiang 150001, China; School of Computer Science and Technology, Harbin Institute of Technology, No. 92 Xidazhi Street, Nangang District, Harbin, Heilongjiang 150001, China; College of Computer and Control Engineering, Northeast Forestry University, No. 26 Hexing Road, Xiangfang District, Harbin, Heilongjiang 150040, China; College of Life Sciences, Northeast Forestry University, No. 26 Hexing Road, Xiangfang District, Harbin, Heilongjiang 150040, China; College of Computer and Control Engineering, Northeast Forestry University, No. 26 Hexing Road, Xiangfang District, Harbin, Heilongjiang 150040, China

**Keywords:** Bayesian network inference, core regulatory triplets, stemness-related lncRNAs, prognostic, immunotherapy

## Abstract

Long noncoding RNAs (lncRNAs) play critical roles in regulating stemness signature genes (SSGs) and tumor immunity, thereby shaping the tumor microenvironment and antitumor immune responses. Increasing evidence suggests that cancer stem cell traits are closely associated with immune evasion and therapeutic resistance, underscoring the need to systematically characterize the pan-cancer interplay among SSGs, lncRNAs, and tumor immunity. Here, we developed an integrative analytical framework that combines network-based modeling with Bayesian network inference to identify core regulatory triplets (STEM-LncCRTs), each consisting of an lncRNA, an SSG, and an immune gene. We demonstrate that specific stemness-related lncRNAs can distinguish cancer subtypes, and that common stemness-related lncRNAs correlate significantly with immune cell infiltration. Notably, the ATAD5/PRR11-AS1/SKP2 triplet exhibits favorable prognostic potential across multiple cancers and consistently outperforms individual gene markers in predicting 1-, 3-, and 5-year overall survival. Furthermore, using four machine learning algorithms across three independent immunotherapy cohorts, we validate the predictive value of STEM-LncCRTs for immune checkpoint inhibitor response. Importantly, integrating STEM-LncCRTs with tumor mutation burden further improves predictive accuracy. Collectively, this study provides a systems-level view of stemness-related lncRNA regulation in tumor immunity and offers practical biomarkers for predicting immunotherapy efficacy.

## Graphical abstract

**Graphical Abstract:**
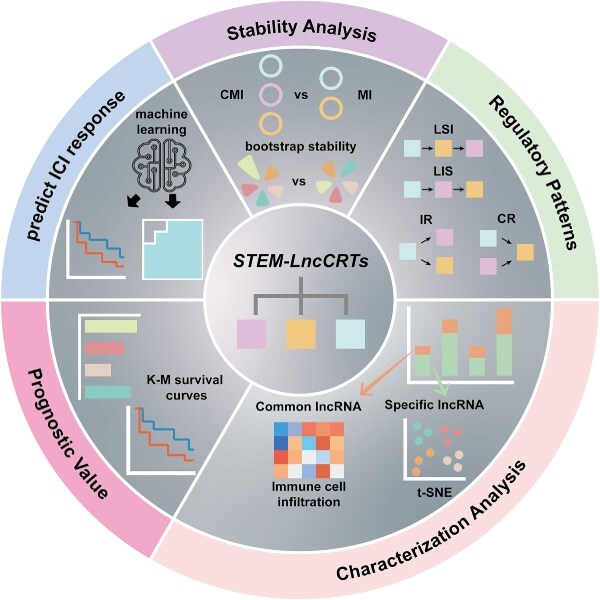

## Introduction

Cancer currently ranks as the second leading cause of death worldwide, following cardiovascular diseases [1]. Despite continuous advances in treatment strategies, the mortality rates of many cancer types remain alarmingly high [2]. With the emergence of targeted therapies, combination regimens such as atezolizumab (an anti-PD-L1 antibody) and bevacizumab (an anti-VEGF antibody) have demonstrated superior efficacy in improving patient prognosis across various cancers [3]. In addition, stem cell therapy has emerged as a promising treatment approach following conventional drug and surgical therapies. Currently, stem cell-based clinical trials are being conducted for nearly a hundred diseases, including various cancers, Alzheimer’s disease, and diabetes [4]. Recent studies have shown that both immune evasion and cancer stem cells (CSCs) are considered essential contributors to tumor growth and metastasis. Moreover, across 21 types of solid malignancies, a high stemness signature has been associated with a poor immunogenic response, highlighting a potential interaction between these two protumorigenic pathways [5]. Based on these associations, the present study links tumor stemness with immune regulation and proposes novel therapeutic strategies that simultaneously target CSCs and the immune system, thereby providing a theoretical foundation for the development of future cancer treatments.

Dysregulation of gene expression programs has been recognized as a key pathogenic factor in a wide range of human diseases [6]. In the human body, transcriptional regulation is precisely orchestrated by thousands of regulatory factors, including transcription factors, chromatin modifiers, and noncoding RNAs (ncRNAs) [7]. Although transcriptomic studies in cancer have identified a large number of long noncoding RNAs (lncRNAs) associated with tumorigenesis and progression [8], their functional roles and underlying mechanisms in tumor biology remain to be systematically elucidated. In recent years, a growing body of evidence has confirmed that lncRNAs play critical roles in regulating CSC functions during tumor progression. For instance, LncCCAT1 promotes stemness in breast cancer by activating the WNT/β-catenin signaling pathway [9]; lncRNA PKMYT1AR facilitates CSC maintenance in non-small cell lung cancer through activation of the Wnt pathway [10], and lncRNA INHEG is aberrantly upregulated in glioma cells, enhancing CSC self-renewal and tumorigenicity by modulating rRNA 2′-O-methylation [11]. Therefore, elucidating the complex regulatory mechanisms among lncRNAs, stemness-associated markers, and the immune system will expand our understanding of cancer biology and provide valuable insights for the development of effective therapeutic strategies targeting malignancies.

In this study, we proposed a computational framework that integrates network-based analysis with Bayesian network inference to identify stemness-related lncRNAs and infer the regulatory patterns of STEM-LncCRTs. Using Bayesian networks and maximum likelihood estimation, we inferred four major regulatory patterns. Immune genes were found to play pivotal roles in the process by which lncRNAs regulate SSGs. Some stemness-related lncRNAs exhibited broad or specific functions in various immune processes, among which cancer-type-specific lncRNAs could serve as molecular features to distinguish cancer types. Our analyses further revealed that stemness-related lncRNAs are closely correlated with immune cell infiltration in tumors. Notably, the ATAD5/PRR11-AS1/SKP2 triplet exhibited robust prognostic value across multiple cancer types, and its predictive performance outperformed that of any individual gene within the STEM-LncCRT. Moreover, the predictive effectiveness of multiple STEM-LncCRTs for ICI response was validated using four machine learning algorithms across three independent datasets. Importantly, combining STEM-LncCRTs with TMB significantly enhanced the predictive accuracy for ICI response in treated patients. In summary, in the era of precision medicine, our approach provides a novel perspective for deciphering the regulatory mechanisms of lncRNAs in controlling SSGs and tumor immunity, and identifies robust biomarkers that can improve prognosis evaluation and immunotherapy benefit prediction in cancer patients.

## Materials and methods

### Sources and preprocessing of data

RNA-seq transcriptomic data from 25 cancer types were downloaded from The Cancer Genome Atlas (TCGA) via the UCSC Xena platform (https://xena.ucsc.edu/). For cancer types where the number of available adjacent normal samples in TCGA was insufficient (*n* ≤ 5), we supplemented the analysis with normal tissue samples from the Genotype-Tissue Expression (GTEx, https://www.gtexportal.org/home/index.html) project, selecting tissues of the same anatomical origin as the corresponding cancer (Supplementary Table 1). Gene expression levels were first converted to transcripts per million (TPM) values and subsequently normalized to log_2_(TPM + 1) to ensure comparability across samples [12]. Batch effects between TCGA and GTEx samples were then corrected using the ComBat algorithm implemented in the “sva” package. Finally, genes with zero expression in more than 70% of the samples were excluded from further analysis. Transcriptome data of melanoma patients receiving immunotherapy were obtained from the Tumor Immunotherapy Gene Expression Resource [13] (TIGER, http://tiger.canceromics.org).

### Identification of SSGs

In this study, to systematically identify SSGs, we employed a comprehensive gene identification strategy integrating three approaches: (i) Database mining: stemness-related genes were collected from StemChecker, a specialized database of stem cell-related genes; (ii) Gene expression profiling: we analyzed expression data from stem cell samples, including embryonic stem cells and induced pluripotent stem cells, and compared them to normal adult tissue samples. Differentially expressed genes (DEGs) between these groups were identified as candidate stemness signature genes; (iii) Random walk with restart (RWR): using a protein–protein interaction (PPI) network, we applied the RWR algorithm to evaluate the proximity of genes to known stemness-related genes, thereby identifying additional stemness candidates.

(i) Database: a total of 26 stemness gene sets were obtained from the StemChecker database [14] (http://stemchecker.sysbiolab.eu/), comprising 4420 stemness-related genes (Supplementary Table 2); (ii) Gene expression profiles: one microarray dataset and three RNA-seq datasets were retrieved from the Gene Expression Omnibus [15] (GEO, https://www.ncbi.nlm.nih.gov/geo/), the GTEx [16], and the Progenitor Cell Biology Consortium [17] (PCBC, https://www.synapse.org/Synapse:syn1773109/wiki/54962) (Supplementary Table 3). DEGs were identified using the “limma” package in R, with thresholds set at |log_2_(Fold Change)| > 1 and a false discovery rate (FDR) < 0.05. (iii) RWR: the human PPI network was obtained from the STRING database [18] (https://string-db.org/), and to ensure high confidence in the constructed network, only interactions with a combined score greater than 700 were retained. First, we designated the classical stemness factors (LIN28, KLF4, NANOG, OCT4, and SOX2) as seed nodes [19, 20]. Specifically, a value of 1 was assigned to these stemness factors, while all other genes in the network were assigned a value of 0, serving as the input for the personalization vector in the algorithm. The formula for the RWR algorithm is as follows:


$$p\left(t+1\right)=\left(1-r\right) Ap(t)+ rp(0)$$

In this context, A denotes the normalized adjacency matrix, and $p(t)$ represents the probability vector at time step $t$. Typically, each node in the seed gene set is assigned an equal initial probability, while all other nodes are assigned an initial probability of zero. Therefore, $p(0)$ serves as the initial probability vector, representing the seed nodes from which the random walk is initiated. The parameter $r$ denotes the restart probability, and $1-r$ corresponds to the probability of continuing the walk. In this study, the restart probability $r$ was set to 0.85. After network propagation, the top 5% of genes with the highest influence scores were defined as proximal target genes.

Furthermore, we manually curated SSGs from previously published studies, all of which have been validated by robust experimental evidence (Supplementary Table 4).

### Identification of lncRNAs, immune genes, and SSGs that may have ternary regulatory relationships in different cancers

First, differentially expressed lncRNAs, immune genes, and SSGs between various cancer types and normal tissue samples were identified using the thresholds of |log₂(Fold Change)| > 1 and FDR < 0.05. Subsequently, a co-expression network was constructed among lncRNAs, immune genes, and SSGs, in which the correlation coefficients were assigned as edge weights. Next, the Page-Rank algorithm was employed to identify lncRNAs and immune genes that are closely associated with SSGs through network propagation. Specifically, a personalization vector was used in which SSGs were assigned a value of 1, and all other genes in the network were assigned a value of 0. Default settings were applied for all other parameters of the Page-Rank algorithm. After network propagation, the top 200 immune genes or lncRNAs with the highest influence scores were selected as those most closely associated with SSGs.

To eliminate the confounding effect of tumor purity, we further refined the correlations among lncRNAs, immune genes, and SSGs using partial correlation analysis. The expression profiles of lncRNA, immune gene, and SSG were defined as $L(i)=\left({l}_1,{l}_2,{l}_3,\dots, {l}_m\right)$, $G(i)=\left({g}_1,{g}_2,{g}_3,\dots, {g}_m\right)$, $S(i)=\left({s}_1,{s}_2,{s}_3,\dots, {s}_m\right)$, respectively. Tumor purity scores were defined as $P=\left({p}_1,{p}_2,{p}_3,\dots, {p}_n\right)$. We first calculated the partial correlation coefficient between the expression of ${lncRNA}_i$ and ${SSG}_j$ while controlling for tumor purity as a covariate:


$$PCC(ij)=\frac{R_{LS}-{R}_{LP}\ast{R}_{SP}}{\sqrt{1-{R}_{LP}^2}\ast \sqrt{1-{R}_{SP}^2}}$$

where ${R}_{LS}$, ${R}_{LP}$, and ${R}_{SP}$ represent the correlation coefficients between ${lncRNA}_i$ expression and ${SSG}_j$ expression, ${lncRNA}_i$ expression and tumor purity, and ${SSG}_j$ expression and tumor purity, respectively.

Subsequently, gene set enrichment analysis (GSEA) was performed to identify immune genes regulated by lncRNAs and SSGs, thereby pinpointing those enriched in 18 immune-related pathways (Supplementary Table 5). Based on the results of both GSEA and partial correlation analysis, we computed the weighted partial-correlation regulatory score (WPC score) for each lncRNA-SSG gene pair:


\begin{align*} WPC=&\sum_{i=1}^n{\beta}_i\left({-\mathit{\log}}_{10}\left({P}_i^{(1)}\right)\ast \mathit{\operatorname{sign}}\left({cor}_i^{(1)}\right) \right. \\&\left. -\,{\mathit{\log}}_{10}\left({P}_i^{(2)}\right)\ast \mathit{\operatorname{sign}}\left({cor}_i^{(2)}\right)\right) \end{align*}

In this equation, *n* represents the number of immune genes co-regulated by lncRNAs and SSGs. ${\beta}_i$ denotes the enrichment score of immune gene obtained from GSEA. ${P}_i^{(1)}$ and ${P}_i^{(2)}$ are the *P*-values from partial correlation analyses between the lncRNA and immune gene, and between the SSG and immune gene, respectively. ${cor}_i^{(1)}$ and ${cor}_i^{(2)}$ correspond to the partial correlation coefficients for these respective gene pairs.

After calculating the WPC score for each lncRNA-SSG gene pair, we performed 1000 random permutations of sample labels to estimate the significance of each score. The *P*-value for each gene pair was calculated using the following formula:


$$p=\frac{N}{1000}$$

Here, *N* represents the number of times that the WPC score for a given gene pair in the 1000 permutations exceeded the original score. To reduce the likelihood of false-positive gene pairs, the resulting *P*-values were adjusted using the Benjamini–Hochberg (BH) procedure to calculate the FDR. Gene pairs with an FDR < 0.05 were retained. Through this procedure, we identified lncRNAs, SSGs, and immune genes that may be involved in tripartite regulatory relationships. These regulatory relationships were termed stemness-related lncRNA-mediated core regulatory triplets, namely STEM-LncCRTs.

### Construction of a Bayesian inference network model within STEM-LncCRTs

To capture the causal relationships among lncRNAs, SSGs, and immune genes, we constructed a Bayesian inference network model based on the STEM-LncCRTs. Within this framework, we proposed four potential regulatory patterns: IR, CR, LIS, and LSI. For each pattern, we defined the joint probability distribution under the assumption of standard Markov features:


$${P}_{IR}\left(L,S,I\right)=P(L)P\left(S|L\right)P\left(I|L\right)$$

$${P}_{CR}\left(L,S,I\right)=P(L)P(I)P\left(S|L,I\right)$$

$${P}_{LIS}\left(L,S,I\right)=P(L)P\left(I|L\right)P\left(S|I\right)$$

$${P}_{LSI}\left(L,S,I\right)=P(L)P\left(S|L\right)P\left(I|S\right)$$

Here, *L, S*, and *I* represent the expression levels of lncRNAs, SSGs, and immune genes, respectively. Each STEM-LncCRT was assigned to the most likely regulatory pattern by calculating the maximum likelihood estimation. In addition, the Bayesian information criterion (BIC) score and corresponding weight ($\omega$) were computed:


$${BIC}_i=k\ast \ln (n)-2\ast \ln (L)$$

$${\Delta}_i={BIC}_i-{BIC}_{min}$$

$${\omega}_i=\frac{e^{-\frac{1}{2}{\Delta}_i}}{\sum_{i=1}^4{e}^{-\frac{1}{2}{\Delta}_i}}$$

where *L* represents the maximum likelihood value for the given model, *k* denotes the number of parameters in each regulatory model, and *n* is the sample size. The BIC was used for model selection because it balances model fit and model complexity while imposing a stronger penalty on model complexity, thereby reducing the risk of overfitting when comparing multiple regulatory patterns. Finally, the model with the lowest BIC score and the highest $\omega$ was selected as the optimal regulatory pattern for each STEM-LncCRT.

### Bootstrap-based stability analysis of lncRNA-mediated triplets

To evaluate the robustness of lncRNA-mediated core regulatory triplets, we computed both mutual information (MI) and conditional mutual information (CMI) under bootstrap resampling. For each triplet $\left(X,Y,Z\right)$, where *X, Y*, and *Z* denote the expression of an lncRNA, an immune gene, and an SSG, respectively, we estimated $I\left(X;Z\right)$ and $I\left(X;Z|Y\right)$ after discretizing expression values into five equiprobable bins. Repeating the procedure over *B* = 200 bootstrap replicates, we defined the stability probability of a significant MI/CMI relationship as the proportion of replicates exceeding a predefined information threshold (θ = 0.2):


$${S}_{pair}=\frac{1}{B}\sum_{b=1}^B\left[{I}_b\left(X;Z\right)>\theta \right]$$

$${S}_{triplet}=\frac{1}{B}\sum_{b=1}^B\left[{I}_b\left(X;Z|Y\right)>\theta \right]$$

The difference $\Delta S={S}_{triplet}-{S}_{pair}$ reflects the degree to which immune gene *Y* enhances or suppresses the regulatory dependence between 𝑋 and 𝑍.

A higher ${S}_{triplet}$ compared with ${S}_{pair}$ indicates that the triplet relationship (lncRNA-immune gene-SSG) exhibits greater robustness than the lncRNA-SSG gene pair, suggesting a potential mediating or enhancing effect of the immune gene *Y* on this regulatory dependence.

### Functional roles of stemness-related lncRNAs in tumor immunity

Immune genes play a pivotal role in the regulation of SSGs by lncRNAs. Across different cancer types, due to the involvement of distinct immune genes, the same lncRNA-SSG gene pair can exhibit multiple regulatory patterns of STEM-LncCRTs. Therefore, we first defined common lncRNAs as stemness-related lncRNAs that appeared in more than 10 cancer types, and specific lncRNAs as those identified in no more than 2 cancer types. In addition, lncRNA-SSG gene pairs that occurred in more than 10 cancer types and exhibited diverse regulatory patterns due to the involvement of different immune genes were defined as pattern-variable lncRNA-SSG gene pairs.

Subsequently, the immunological characteristics of stemness-related lncRNAs were evaluated from two perspectives: (i) the Tumor Immune Estimation Resource (TIMER) algorithm was applied to estimate the infiltration abundance of five major immune cell types (B cells, CD4^+^ T cells, CD8^+^ T cells, neutrophils, and macrophages) across various cancer types and (ii) immunohistochemistry (IHC) and immunofluorescence (IF) staining were conducted using data from the Human Protein Atlas [21] (https://www.proteinatlas.org/).

### Prognostic analysis of STEM-LncCRTs

We employed both univariate Cox regression analysis and the log-rank test to assess the association between the expression of stemness-related lncRNAs and patient prognosis across various cancer types. Stemness-related lncRNAs with *P* < .05 in both the univariate Cox analysis and log-rank test were considered to be prognostically relevant.

For STEM-LncCRTs, we calculated a triplet score for each patient by linearly weighting the expression values of the three constituent genes using the regression coefficients derived from multivariate Cox regression. The STEM-LncCRT score for each sample was computed as follows:


\begin{align*} STEM- LncCRT\ score=\,&{coef}_{lnc}\ast{\mathit{\exp}}_{lnc}+{coef}_{imm}\ast{\mathit{\exp}}_{imm} \\&+{coef}_{SSG}\ast{\mathit{\exp}}_{SSG} \end{align*}

where ${coef}_{lnc}$, ${coef}_{imm}$, and ${coef}_{SSG}$ denote the Cox regression coefficients for the lncRNA, immune gene, and SSG, respectively, and ${\mathit{\exp}}_{lnc}$, ${\mathit{\exp}}_{imm}$, and ${\mathit{\exp}}_{SSG}$ represent their corresponding expression levels. Subsequently, patients were stratified into high- and low-score groups based on the optimal cutoff value, and survival differences between the two groups were evaluated using the log-rank test.

### Prediction of ICI response based on STEM-LncCRTs

Three melanoma cohorts treated with anti-PD-1 therapy, namely GSE78220 [22], GSE91061 [23], and phs000452 [24], were collected (Supplementary Table 6). After batch effect correction using the “sva” package in R, the datasets were merged, with 70% of the samples used as the training set and the remaining 30% as the validation set. First, STEM-LncCRTs significantly associated with prognosis were identified using the log-rank test. Subsequently, the least absolute shrinkage and selection operator (LASSO) regression model was applied for dimensionality reduction and feature selection, and the selected STEM-LncCRTs were used as predictive features for model construction. To comprehensively evaluate the predictive performance of the selected STEM-LncCRTs for ICI treatment response, we employed four machine learning algorithms, including random forest, gradient boosting machine, kernel support vector machine, and partial least squares. These approaches allowed us to capture both linear and nonlinear relationships and assess the stability of the selected STEM-LncCRTs under different modeling algorithms. The hyperparameter settings for the machine learning models are provided in Supplementary Table 7. Model performance was evaluated using receiver operating characteristic (ROC) curves, and the optimal model was determined based on predictive accuracy. The STEM-LncCRT with the highest contribution in the optimal model was identified using SHapley Additive exPlanations (SHAP) values. The performance of this optimal STEM-LncCRT was compared with other clinical indicators (PD-L1, IFNG, and CTLA-4). In addition, the IMvigor210 dataset was used to assess the predictive power of combining TMB and the average expression level of STEM-LncCRT in forecasting ICI response.

### Statistical analysis

All statistical analyses were conducted using R software (version 4.4.2). DEGs were identified using the “limma” package. Pearson correlation coefficients were calculated to assess gene–gene correlations. Pattern selection in the Bayesian network inference was performed using the “bnlearn” package. Gene set overlap significance was assessed using the hypergeometric test via the phyper function. The TIMER algorithm in the “IOBR” package was employed to assess immune cell infiltration proportions. Survival curve comparisons were conducted using the log-rank test, and the optimal cutoff values for stratifying cancer patients were determined using the “survminer” package.

## Results

### Identification of stemness-related lncRNA-mediated core regulatory triplets in pan-cancer

**Figure 1:**
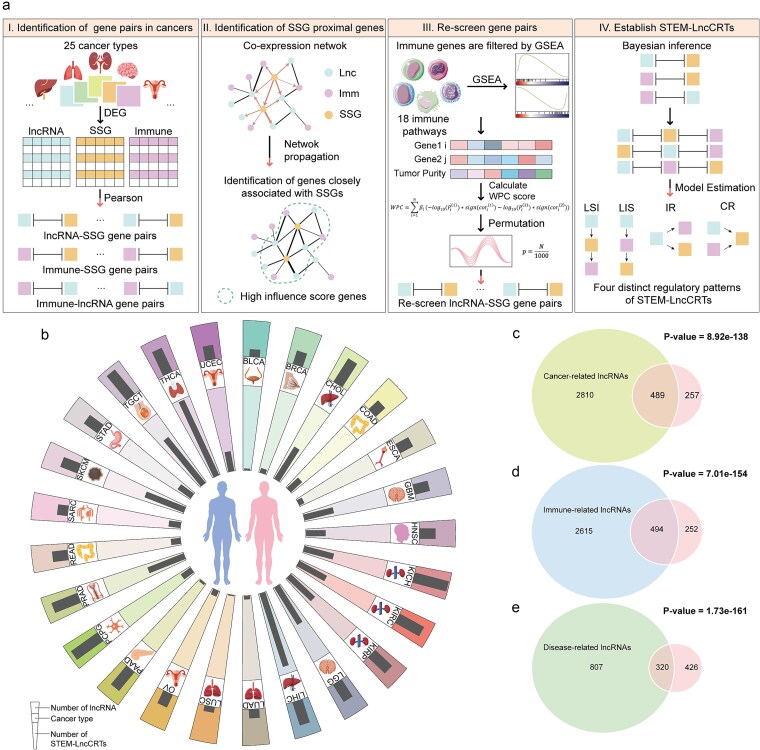
Computational framework for the identification of STEM-LncCRTs. (a) Schematic overview of the computational framework for STEM-LncCRT identification. The four regulatory patterns include: LIS (lncRNAs regulating SSGs via immune genes), LSI (lncRNAs directly regulating SSGs, thereby influencing immune genes), CR (cooperative regulation of SSGs by lncRNAs and immune genes), and IR (independent regulation of both SSGs and immune genes by lncRNAs); (b) the number of stemness-related lncRNAs and STEM-LncCRTs in various cancer type; (c–e) Venn diagram illustrating the intersection between stemness-related lncRNAs and those related to cancer, immunity, and disease, respectively.

To identify core regulatory triplets formed by lncRNAs associated with stemness, we proposed a four-step computational framework (Fig. 1a). This framework integrates gene expression profiles, network modules, and immune-related pathways to comprehensively infer stemness-related lncRNAs and their regulatory modes based on large-scale sample data. We hypothesized that lncRNAs participating in the regulation of both immune genes and SSGs could mediate the crosstalk between these two major signaling pathways, potentially enriching immune-related pathways and enabling dual regulation of tumor stemness and the immune microenvironment. Specifically, STEM-LncCRTs were identified through the following four steps: Step 1. Differentially expressed lncRNAs, immune genes, and SSGs were first extracted from gene expression data of cancer patients. Pearson correlation analysis was then conducted to identify significantly correlated gene pairs; Step 2. A co-expression network was constructed, and the Page-Rank algorithm was employed to identify lncRNAs and immune genes closely associated with SSGs; Step 3. Based on the elimination of tumor purity effects, we further identified lncRNAs, immune genes, and SSGs involved in strong triplet regulatory relationships by integrating partial correlation analysis, GSEA, WPC score, and random perturbation strategies; Step 4. Bayesian inference was used to determine the optimal regulatory pattern for each identified STEM-LncCRT.

Using this four-step computational framework and excluding the effect of sample size, we identified 23–146 stemness-related lncRNAs and 1300–131 881 STEM-LncCRTs across different cancer types (Fig. 1b). These findings suggest that lncRNAs may play diverse and complex roles in the tumor microenvironment by forming extensive STEM-LncCRTs. Moreover, we observed significant interactions between stemness-related lncRNAs and cancer-related, immune-related, and disease-related lncRNAs (Fig. 1c–e).

### The pan-cancer landscape of SSGs

**Figure 2:**
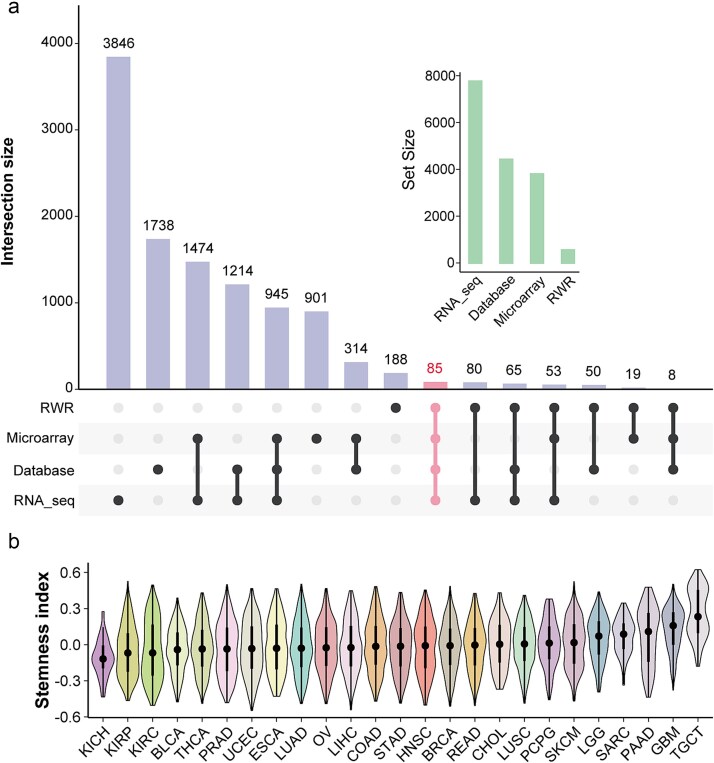
Pan-cancer distribution landscape of the identified SSGs. (a) The UpSet plot illustrates the intersection of SSGs identified by RWR, microarray, database, and RNA-seq, and the upper-right bar plot indicates the number of genes identified by each method; (b) violin plot illustrates the distribution of stemness scores across various cancer types, calculated using the identified SSGs as the background gene set.

According to the procedures described in the “Materials and Methods,” a total of 85 shared SSGs were identified through integrative analysis of public databases, gene expression profiles, and random walk-based approaches (Fig. 2a). Additionally, 84 experimentally validated SSGs were manually curated from previously published literature for subsequent analyses. Based on these 84 SSGs, we performed gene set variation analysis (GSVA) across each cancer type to calculate the stemness scores under the current background gene set. Compared with previously published studies on cancer stemness indices [25], our results showed a high level of concordance (Fig. 2b). Taken together, the identified SSGs are both accurate and of significant research value.

### Bootstrap-based stability analysis of core regulatory triplets

**Figure 3:**
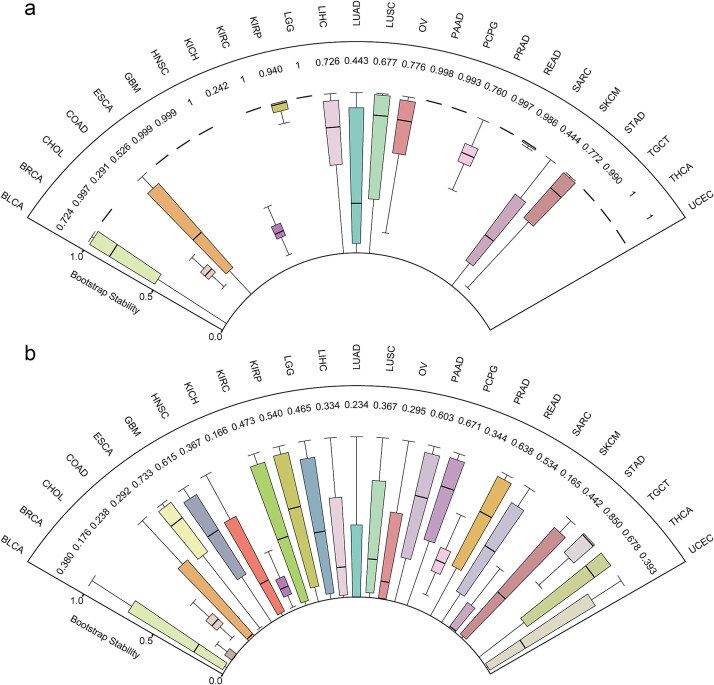
Bootstrap-based stability analysis. The boxplots illustrate the bootstrap stability of (a) triplet and (b) lncRNA-SSG gene pairs across different cancer types.

To evaluate the robustness of the inferred regulatory interactions, we performed a bootstrap-based stability analysis across all triplets. We next assessed the bootstrap stability of lncRNA-SSG gene pair and triplet associations across all cancer types using MI and CMI, respectively. In all cancer types, triplets exhibited higher mean bootstrap stability than lncRNA-SSG gene pairs (Fig. 3a and b). The observed difference in bootstrap stability was statistically significant (Supplementary Fig. 1), suggesting that incorporating the immune gene context improves the robustness and reliability of the inferred regulatory dependencies.

### Regulatory patterns of STEM-LncCRTs across cancer types

**Figure 4:**
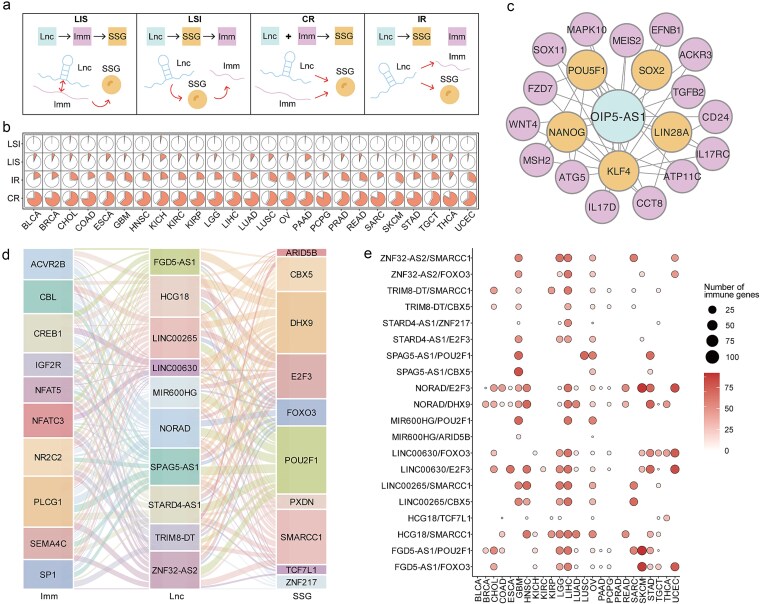
The complex regulatory mechanisms of STEM-LncCRTs. (a) Four inferred regulatory models; (b) the proportions of the four regulatory models across different cancer types; (c) a representative STEM-LncCRT subnetwork centered on OIP5-AS1. Immune genes, SSGs, and lncRNAs are colored in purple, orange, and blue, respectively; (d) a Sankey diagram illustrating that the same lncRNA-SSG gene pair can form distinct STEM-LncCRTs by interacting with different immune genes across various immune pathways; (e) the number of immune genes involved in regulating the same lncRNA-SSG gene pair across different cancer types.

We investigated the regulatory mechanisms by which lncRNAs modulate SSGs through four defined regulatory patterns of STEM-LncCRTs. These patterns include: (i) lncRNAs regulating SSGs via immune genes (LIS); (ii) lncRNAs directly regulating SSGs, which in turn affect immune genes (LSI); (iii) lncRNAs and immune genes cooperatively regulate SSGs (CR); and (iv) lncRNAs independently regulating both SSGs and immune genes (IR) (Fig. 4a). The proportions of these four regulatory patterns varied across cancer types. Among the 25 cancer types analyzed, the CR pattern accounted for the highest proportion (Fig. 4b). This suggests that immune genes exert a significant influence in the regulation of SSGs by lncRNAs under the synergistic regulation model. Furthermore, our results demonstrated that multiple immune genes were involved in nearly all STEM-LncCRTs (Supplementary Fig. 2). For example, the stemness-related lncRNA OIP5-AS1 can regulate SSGs by interacting with immune genes (Fig. 4c). Previous studies have reported that OIP5-AS1 contributes to the enhancement of stemness characteristics in cancer [26, 27]. The immune-regulatory effect of stemness-related lncRNAs depends on the number of immune genes involved in regulating SSGs across different cancer types. Moreover, the same lncRNA-SSG gene pairs can interact with different immune genes to form diverse STEM-LncCRTs complexes, thereby executing their functions through distinct immune pathways (Fig. 4d and e). Taken together, these findings highlight the complex regulatory landscape of STEM-LncCRTs in cancer.

### Characterization analysis of distinct types of stemness-related lncRNAs

**Figure 5:**
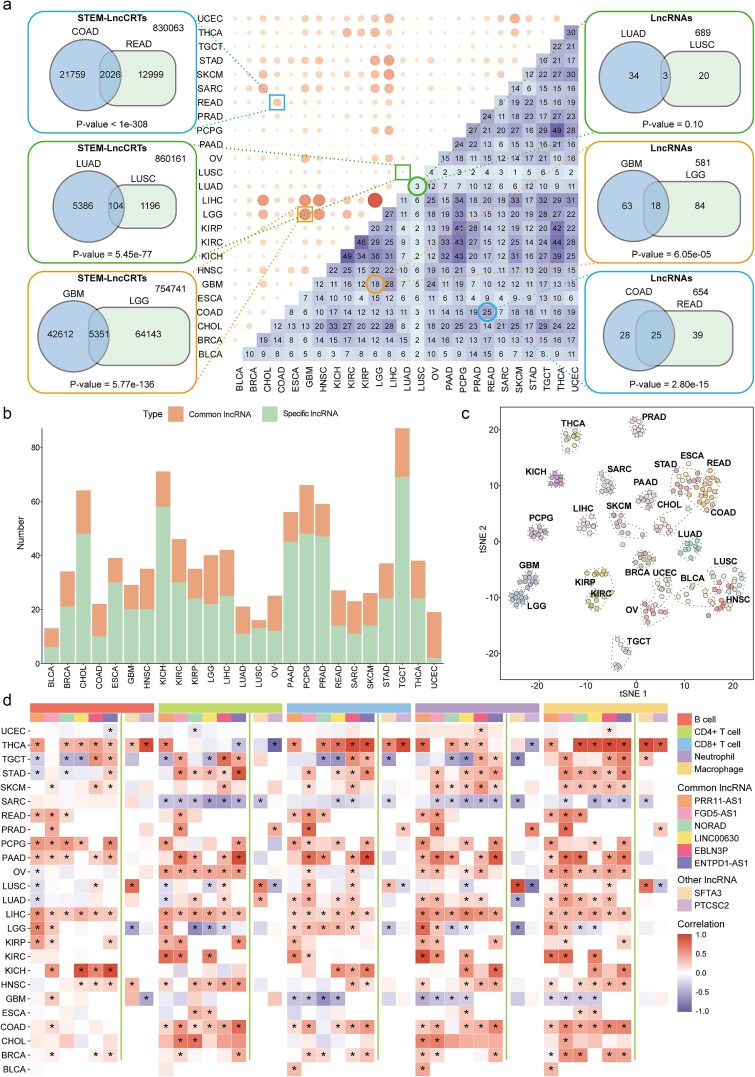
Characterization of distinct types of stemness-related lncRNAs in cancer immunity. (a) The upper triangular heatmap shows the number of shared STEM-LncCRTs between cancer types, while the lower triangular heatmap indicates the overlap of stemness-related lncRNAs between cancer types. Venn diagrams display the overlap in the number of stemness-related lncRNAs and STEM-LncCRTs among different subtypes of colorectal, brain, and lung cancers; (b) the number of common and specific lncRNAs identified in each cancer type; (c) cancer samples were clustered by t-SNE based on the expression of specific lncRNAs; (d) heatmap showing the correlations between common or specific stemness-related lncRNAs and infiltration levels of five immune cell types. “*” indicates statistically significant correlation (*P* < .05).

To further investigate the critical roles of stemness-related lncRNAs across different cancer types, we systematically characterized STEM-LncCRTs in each cancer. Our analysis revealed that cancer subtypes sharing the same tissue of origin exhibited common stemness-related lncRNAs and STEM-LncCRTs (Fig. 5a). For example, in the two colorectal cancer subtypes, COAD and READ, the stemness-related lncRNAs and their STEM-LncCRTs showed considerable similarity, with several STEM-LncCRTs identified in COAD also detected in READ (lncRNAs: *P* = 2.80e-15; STEM-LncCRTs: *P* < 1e-308). Similar observations were made in brain (lncRNAs: *P* = 6.05e-05; STEM-LncCRTs: *P* = 5.77e-136) and lung (lncRNAs: *P* = .10; STEM-LncCRTs: *P* = 5.45e-77) cancer subtypes. Although fewer overlapping lncRNAs were found between the two lung cancer subtypes, LUAD and LUSC, the similarity in STEM-LncCRTs was statistically significant. Based on these findings, we defined lncRNAs identified in two or fewer cancer types as specific lncRNAs, whereas those detected in more than 10 cancer types were considered common lncRNAs. Subsequently, for each cancer type, we constructed and analyzed STEM-LncCRT networks based on their respective regulatory models. With the exception of pancreatic cancer, SSGs consistently exhibited higher node degrees than lncRNAs and immune genes across all networks, indicating that regulatory relationships were predominantly centered around SSGs (Supplementary Fig. 3). Moreover, in the network constructed from common lncRNAs and SSGs, these lncRNAs demonstrated close interactions with SSGs across multiple cancers (Supplementary Fig. 4). Some lncRNAs and genes within this network have been functionally validated in previous studies. For instance, OIP5-AS1 was reported to interact with miR-363-3p and promote hepatocellular carcinoma progression by upregulating SOX4 [28]. Likewise, NORAD was shown to accelerate non-small cell lung cancer progression through the miR-129-1-3p/SOX4 axis [29].

According to the results, these common lncRNAs paired with SSGs were detected across multiple cancer types; however, the immune genes involved in their corresponding triplets and the associated regulatory mechanisms varied among cancer types, indicating a degree of diversity. Most lncRNA-SSG gene pairs were associated with 20 to 80 immune genes and involved at least 2 distinct regulatory modes (Supplementary Fig. 5). Among them, FGD5-AS1 was identified as one of the common stemness-related lncRNAs. In the lncRNA-SSG gene pair composed of FGD5-AS1 and SMARCC1, multiple immune genes were involved, and diverse regulatory patterns were observed. In this study, such gene pairs were referred to as “pattern-variable lncRNA-SSG gene pairs”. These findings suggest that the regulatory associations between lncRNAs and SSGs are highly complex.

For specific lncRNAs, we randomly selected 10 patients from each cancer type for further characterization. The number of specific lncRNAs varied considerably across different cancer types (Fig. 5b). For instance, TGCT exhibited the highest number with 69 identified cancer-specific lncRNAs, whereas UCEC had the lowest number, with only 2 identified. We further validated the expression patterns of these cancer-specific lncRNAs using t-distributed stochastic neighbor embedding (t-SNE) (Fig. 5c). The results showed that samples from different cancer types could be clearly distinguished, with those of similar tissue origin or from the same cancer subtype clustering together. This included core digestive system cancers (ESCA, STAD, COAD, and READ) and squamous cell carcinomas (LUSC and HNSC), indicating shared expression patterns among specific lncRNAs. Overall, these findings suggest that stemness-related lncRNAs exhibit both common and specific regulatory characteristics, potentially contributing to tumorigenesis and modulation of the immune microenvironment in diverse manners.

### Association between stemness-related lncRNAs and immune cell infiltration

An increasing body of evidence suggests a broad correlation between cancer stem cells and immune cell responses [5]. Therefore, if stemness-related lncRNAs play critical roles in immune regulation, their expression should be associated with immune cell infiltration in tumors. To investigate this, we estimated the infiltration levels of five immune cell types, including B cells, CD4+ T cells, CD8+ T cells, macrophages, and neutrophils, based on gene expression data from each cancer type using the TIMER algorithm. The results revealed that the top five common lncRNAs were significantly correlated with immune cell infiltration (Fig. 5d). Compared to other stemness-related lncRNAs, these common lncRNAs showed stronger correlations with immune infiltration. Furthermore, IHC and IF staining demonstrated that certain immune genes within STEM-LncCRTs, such as IGF2R, exhibited higher protein expression levels in tumor tissues than in normal tissues (Supplementary Fig. 6). Taken together, these findings support a significant association between stemness-related lncRNAs and immune cell infiltration, further validating the effectiveness and rationality of our proposed computational framework.

### STEM-LncCRTs as predictive biomarkers for cancer prognosis

**Figure 6:**
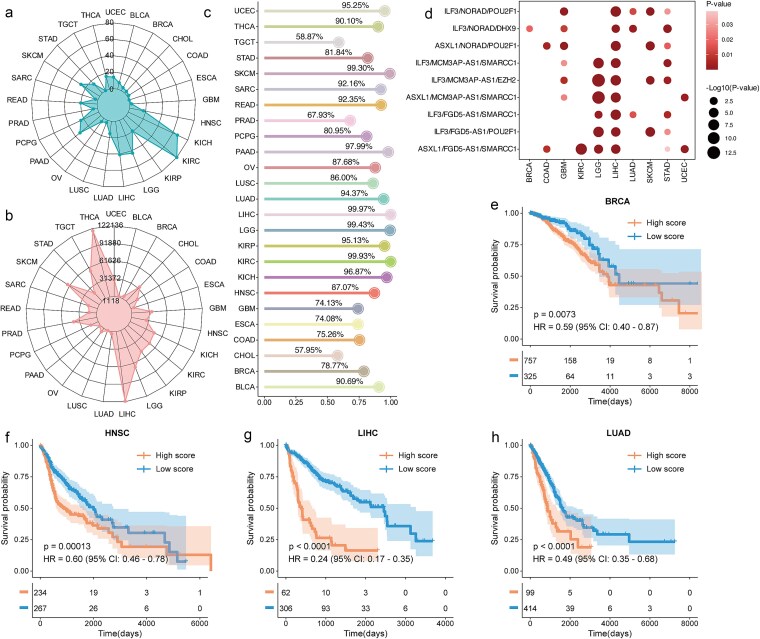
Prediction of cancer prognosis. (a) Radar plot showing the number of stemness-related prognostic lncRNAs across various cancer types; (b) radar plot showing the number of prognostic STEM-LncCRTs across various cancer types; (c) percentage of prognostic STEM-LncCRTs in each cancer type; (d) prognostic significance of 9 STEM-LncCRTs across 10 types of cancer; (e–h) Kaplan–Meier survival curves for the ATAD5/PRR11-AS1/SKP2 in BRCA, HNSC, LIHC, and LUAD.

Previous studies have demonstrated that stemness-related biomarkers play pivotal roles in cancer initiation and progression, highlighting their prognostic potential [30–32]. Therefore, we investigated whether these stemness-related lncRNAs, particularly STEM-LncCRTs, are associated with patient survival across cancer types. Our analysis revealed that a large number of stemness-related lncRNAs and STEM-LncCRTs were significantly correlated with overall survival in various cancers (Fig. 6a and b). Notably, in LIHC, 99.97% of the identified STEM-LncCRTs were associated with patient overall survival (Fig. 6c). Moreover, certain STEM-LncCRTs may serve as prognostic biomarkers within the same cancer type (Fig. 6d). Among these, NORAD, MCM3AP-AS1, and FGD5-AS1 participated in prognostically relevant STEM-LncCRTs across different cancers. In previous studies, NORAD has been reported to be involved in multiple cancer-related processes, including cell proliferation, apoptosis, invasion, and metastasis [33]. Abnormal expression of MCM3AP-AS1 has been associated with the progression of several malignancies, such as colorectal cancer, lung cancer, ovarian cancer, hepatocellular carcinoma, and breast cancer [34]. Moreover, FGD5-AS1 was found to be highly expressed in multiple tumor tissues and strongly associated with lymph node metastasis, tumor invasion, survival, and recurrence in various cancers [35].

Based on the STEM-LncCRT score calculation (as described in the Materials and Methods), ATAD5/PRR11-AS1/SKP2 was significantly associated with patient prognosis across 16 cancer types (Supplementary Table 8). Therefore, we selected BRCA, HNSC, LIHC, and LUAD as representative cancer types to further explore its prognostic potential. Our results showed that a lower ATAD5/PRR11-AS1/SKP2 STEM-LncCRT score was significantly associated with improved survival outcomes in cancer patients, based on the optimal cutoff determined using the “survminer” package (Fig. 6e–h). Compared with individual genes, this STEM-LncCRT showed better prognostic performance for patient survival (Supplementary Fig. 7). Moreover, in terms of survival prediction, the STEM-LncCRT consistently exhibited superior prognostic performance compared with any of its individual genes (Supplementary Fig. 8). The STEM-LncCRT score also outperformed individual genes in predicting 1-, 3-, and 5-year survival outcomes (Supplementary Fig. 9). Multivariate Cox regression analysis demonstrated that ATAD5/PRR11-AS1/SKP2 could serve as an independent prognostic factor for overall survival (Supplementary Fig. 10). Collectively, these results indicate that ATAD5/PRR11-AS1/SKP2 may serve as a robust prognostic biomarker across multiple cancer types.

### STEM-LncCRTs enhance the predictive power for ICI response

**Figure 7:**
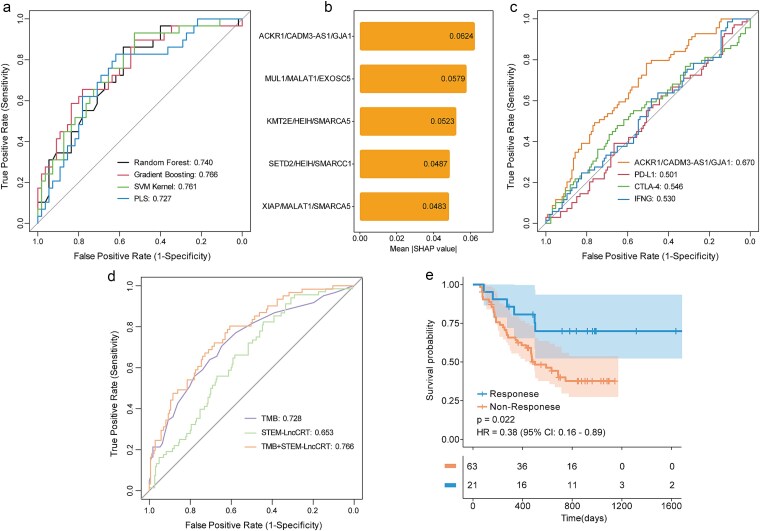
STEM-LncCRTs can predict ICI response. (a) ROC curves of four machine learning algorithms constructed using STEM-LncCRTs to predict ICI responses; (b) SHAP value ranking of features in the optimal predictive model; (c) ROC curves of the ability of ACKR1/CADM3-AS1/GJA1 to predict ICI response with that of other immune checkpoint markers; (d) ROC curves for predicting treatment response using STEM-LncCRTs, TMB, and their combination; (e) Kaplan–Meier survival curves for melanoma patients stratified by model-predicted response to ICI therapy.

To evaluate the potential of STEM-LncCRTs in predicting ICI responses, we integrated three anti-PD-1-treated melanoma datasets. Based on our computational framework, a total of 648 prognostic STEM-LncCRTs were identified, among which 33 were further selected using the LASSO regression model. Four machine learning algorithms, including random forest, gradient boosting machines, kernel support vector machine, and partial least squares, were employed to construct predictive models for ICI response. All models demonstrated robust performance with area under the ROC curve (AUC) values exceeding 0.7, with gradient boosting achieving the best performance (AUC = 0.766; Fig. 7a). SHAP analysis of the optimal model revealed that the triplet ACKR1/CADM3-AS1/GJA1 had the highest predictive contribution (Fig. 7b). Notably, when compared with classical immune checkpoint markers such as PD-L1, CTLA-4, and IFNG, the ACKR1/CADM3-AS1/GJA1 triplet exhibited the highest AUC (Fig. 7c).

Patients with high TMB are typically considered more responsive to ICI therapy; however, TMB alone has limited predictive capacity. We therefore investigated the predictive efficacy of combining STEM-LncCRTs with TMB in the IMvigor210 immunotherapy cohort. When used individually, STEM-LncCRTs and TMB yielded AUCs of 0.653 and 0.728, respectively. In contrast, the combined model significantly improved the predictive accuracy with an AUC of 0.766 (Fig. 7d). Kaplan–Meier survival analysis further confirmed that patients predicted to respond to treatment had significantly longer overall survival compared to nonresponders (Fig. 7e). Collectively, these findings suggest that STEM-LncCRTs are effective predictors of ICI treatment response and that their combination with TMB markedly enhances predictive performance.

## Discussion

CSCs, also referred to as tumor-initiating cells, possess the abilities of self-renewal and multi-lineage differentiation. CSCs represent a core cellular subpopulation that drives tumor progression, heterogeneity, and metastatic dissemination [36]. An increasing body of evidence suggests that these cells can evade immune surveillance through specific immune escape mechanisms [37, 38]. For instance, SSGs such as SOX2 and OCT4 are downregulated during differentiation, leading to impaired antigen presentation and a consequent reduction in immune memory responses [25]. lncRNAs can regulate SSGs through both direct and indirect mechanisms. Therefore, comprehensive characterization of the core regulatory circuitry composed of lncRNAs, immune genes, and SSGs across cancers is of great significance.

In this study, we performed an integrative analysis involving 9420 patients across 25 different cancer types. We developed a complex computational framework to identify stemness-related lncRNAs and their regulatory patterns across multiple cancers (Fig. 1a). While previous studies have identified immune-related lncRNAs [39], our work specifically focuses on stemness-related lncRNAs and explores their complex regulatory roles within the tumor microenvironment. We further investigated the causal relationships between SSGs and lncRNAs. Additionally, the predictive performance of the proposed STEM-LncCRT was validated across multiple cohorts, and it also showed potential for predicting cancer patients’ responses to immunotherapy. Collectively, our findings reveal a complex regulatory landscape involving SSGs, lncRNAs, and immune genes, providing feasible targets and novel insights for further exploration and therapeutic development.

To ensure the accuracy of the entire computational framework, we first performed a precise identification of SSGs. During this process, we integrated stem cell-related datasets, public databases, and the RWR algorithm for initial screening (Fig. 2a). Specifically, the RWR algorithm was conducted based on the PPI network from the STRING database, thereby constraining the selection of SSGs from a biological perspective. Subsequently, experimentally supported SSGs were identified through relevant literature. These curated SSGs were then used as the background gene set to calculate the stemness index (SI) for each cancer type using GSVA (Fig. 2b). Consistent with previous studies, TGCT exhibited the highest SI, and the SI distribution across other cancer types also showed a high degree of concordance with published results [25]. These findings confirm the accuracy and biological relevance of the identified SSGs.

To further elucidate the regulatory effects of lncRNAs on SSGs mediated by immune genes, as well as their roles in tumor immune regulation, we identified stemness-related lncRNAs and constructed core regulatory triplets composed of lncRNAs, immune genes, and SSGs. During this process, we applied the PageRank algorithm within the constructed co-expression network to prioritize lncRNAs and immune genes that are closely associated with SSGs. Next, we proposed potential regulatory patterns dominated by stemness-related lncRNAs and inferred the most likely regulatory mode for each STEM-LncCRT using Bayesian network modeling and maximum likelihood estimation. Among the four hypothesized regulatory patterns analyzed, the CR pattern accounted for the largest proportion across cancer types, whereas the LSI pattern was the least represented. It is important to note that this study focuses on four fundamental regulatory patterns, though the mechanisms by which lncRNAs regulate SSGs are likely more diverse and complex. While these hypothesized models may have limitations, such as the reliance of Bayesian network inference on prior knowledge, they nonetheless provide valuable mechanistic insights for exploring immune regulation in the context of cancer stemness. The inferred causal relationships warrant further experimental validation *in vitro* and *in vivo*.

In complex regulatory systems, associations inferred from high-dimensional expression data are often influenced by sampling variability and noise. Direct MI between two genes may reflect apparent dependencies driven by shared regulatory contexts rather than true causality. To ensure robustness, we implemented a bootstrap-based stability framework that quantifies the reproducibility of information-theoretic dependencies under repeated resampling. For each triplet, both unconditional MI and conditional MI were estimated across multiple bootstrap replicates, and the stability probability was defined as the proportion of resampled datasets in which a dependency remained significant. Comparing triplet and lncRNA-SSG gene pair stability provides a quantitative measure of whether the inclusion of an immune gene enhances or weakens the regulatory link between lncRNA and SSG. A higher triplet stability indicates that immune genes act as stabilizing mediators that reinforce reproducible regulatory dependencies. Methodologically, this framework extends beyond single-point MI or CMI estimation, offering a probabilistic assessment of reproducibility that distinguishes genuine biological regulation from spurious correlation. Conceptually, it highlights the contribution of immune context in shaping robust transcriptional dependencies, an essential feature for deciphering reliable regulatory circuits within heterogeneous tumor microenvironments.

In the era of immunotherapy-driven precision medicine, the development of effective biomarkers for predicting patient prognosis and response to ICIs remains a central challenge. Although several biomarkers, such as PD-L1 expression, TMB, and specific genes, have been approved or validated by the US Food and Drug Administration (FDA), they still suffer from two major limitations: insufficient cross-cancer applicability and suboptimal predictive specificity. Therefore, there is an urgent need to identify novel, high-precision biomarkers to improve ICI response prediction. Based on our analyses, the ATAD5/PRR11-AS1/SKP2 triplet demonstrated superior prognostic performance compared with individual genes across multiple cancer types, showing improved prediction of 1-, 3-, and 5-year overall survival. Notably, the ATAD5/PRR11-AS1/SKP2 triplet served as an independent prognostic factor across multiple cancer types. In integrated melanoma cohorts, STEM-LncCRTs outperformed several conventional immune checkpoint biomarkers in predictive accuracy, as assessed by multiple machine learning algorithms. Moreover, combining STEM-LncCRTs with TMB further enhanced the predictive power for ICI treatment response. In future studies, the prognostic utility of additional STEM-LncCRTs should be validated across more cancer types. Furthermore, ICI-related cohorts should be expanded, and the computational algorithms used for prediction should be continuously optimized.

Although the computational framework developed in this study was primarily based on large-scale bulk RNA-seq datasets, we plan to extend its application to single-cell RNA-seq (scRNA-seq) data in future research to further validate the robustness and effectiveness of our approach. As scRNA-seq datasets continue to grow in sample size, cancer type coverage, and associated clinical information on immune cells, they will provide valuable opportunities for obtaining and dissecting more precise regulatory relationships between SSGs and lncRNAs.

In summary, by integrating bulk RNA-seq data, clinical information, and immunotherapy datasets, we further uncovered the regulatory relationships among SSGs, lncRNAs, and immune genes in cancer. The identified STEM-LncCRT ATAD5/PRR11-AS1/SKP2 exhibited superior performance over its individual molecular markers in prognostic evaluation and in predicting 1-, 3-, and 5-year overall survival, and further demonstrated favorable potential across multiple cancer types. We also validated that multiple STEM-LncCRTs possess robust predictive power for ICI response. Notably, combining STEM-LncCRTs with TMB significantly enhanced the ability to predict treatment responses in ICI-treated cancer patients. These findings offer promising candidate regulatory axes for further investigation into the immunological functions and mechanisms of stemness-related lncRNAs and may provide valuable insights for future therapeutic strategies.

**Key Points**

- This study provides a strategy based on transcriptomic data and Bayesian network inference models to identify lncRNA-stemness-immune gene regulatory triplets, termed STEM-LncCRT, and further delineates distinct regulatory patterns.
- Mutual information-based stability analyses demonstrated that triplets consistently showed greater stability than gene pairs across all cancer types.
- Specific stemness-related lncRNAs distinguish cancer subtypes, whereas common stemness-related lncRNAs are associated with immune cell infiltration.
- Comprehensive characterization and analysis of STEM-LncCRT revealed the distinct regulatory patterns mediated by lncRNAs across different cancers and highlighted the potential of STEM-LncCRT as a prognostic biomarker in clinical applications.

## Supplementary Material

Supplementary_File_bbag287

## Data Availability

The TCGA datasets were downloaded from the UCSC Xena platform (http://xena.ucsc.edu/). Immunotherapy response data was obtained from Tumor Immunotherapy Gene Expression Resource (http://tiger.canceromics.org/). The source code is publicly available at https://github.com/Sincere-qzp/STEM-LncCRTs.
