# Supplementary material for: Identification and characterization of lncRNA-stemness-immune regulatory patterns
Source: Brief Bioinform. 2026 Jun 4;27(3):bbag287. doi: 10.1093/bib/bbag287 (PMC13235729; doi:10.1093/bib/bbag287)
Supplement: Supplementary_File_bbag287 [file supplementary_file_bbag287.docx]

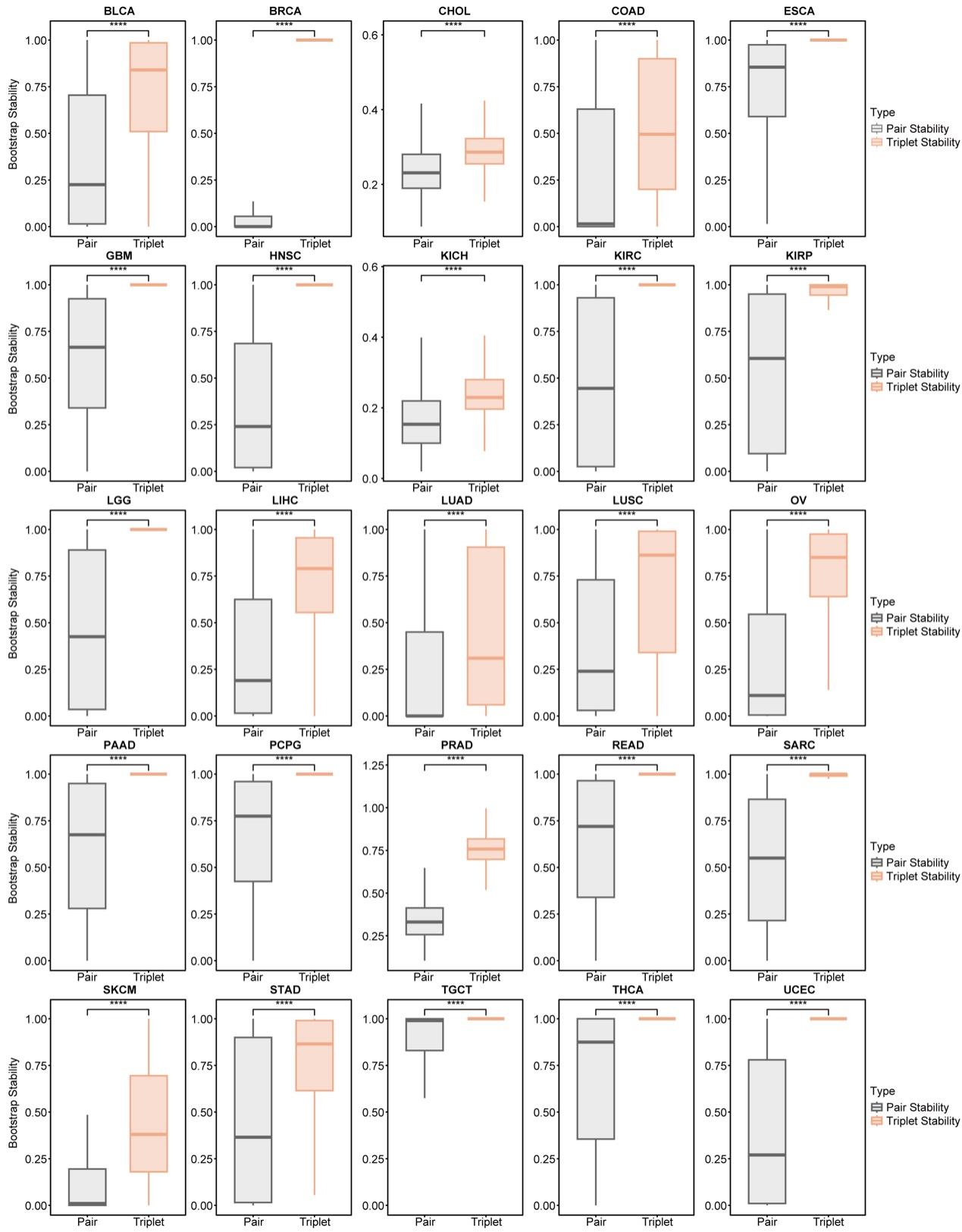


**Supplementary Figure 1.** Bootstrap-based stability analysis of pairwise and triplet.


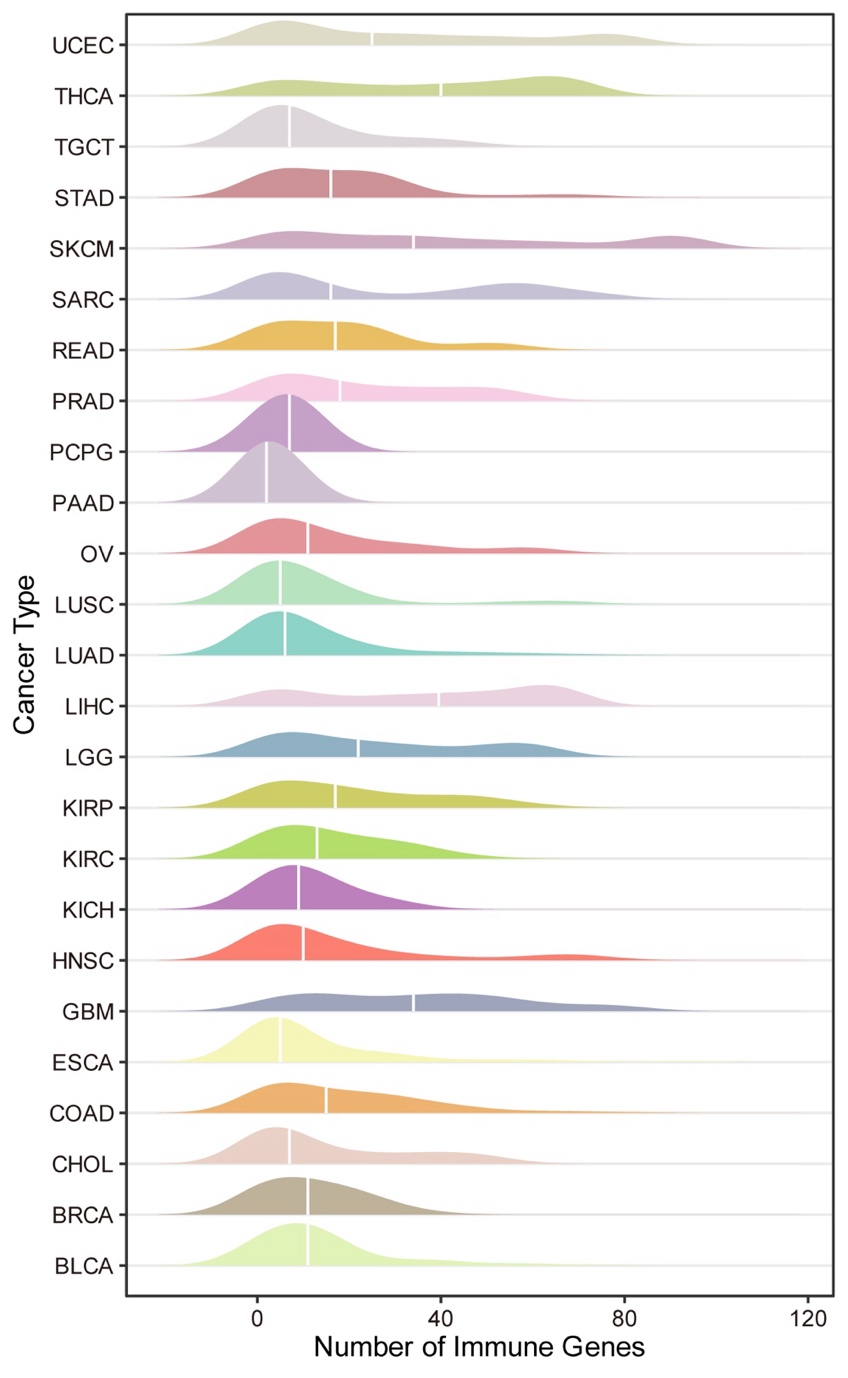


**Supplementary Figure 2.** Distribution of immune genes in STEM-LncCRTs across all cancers.


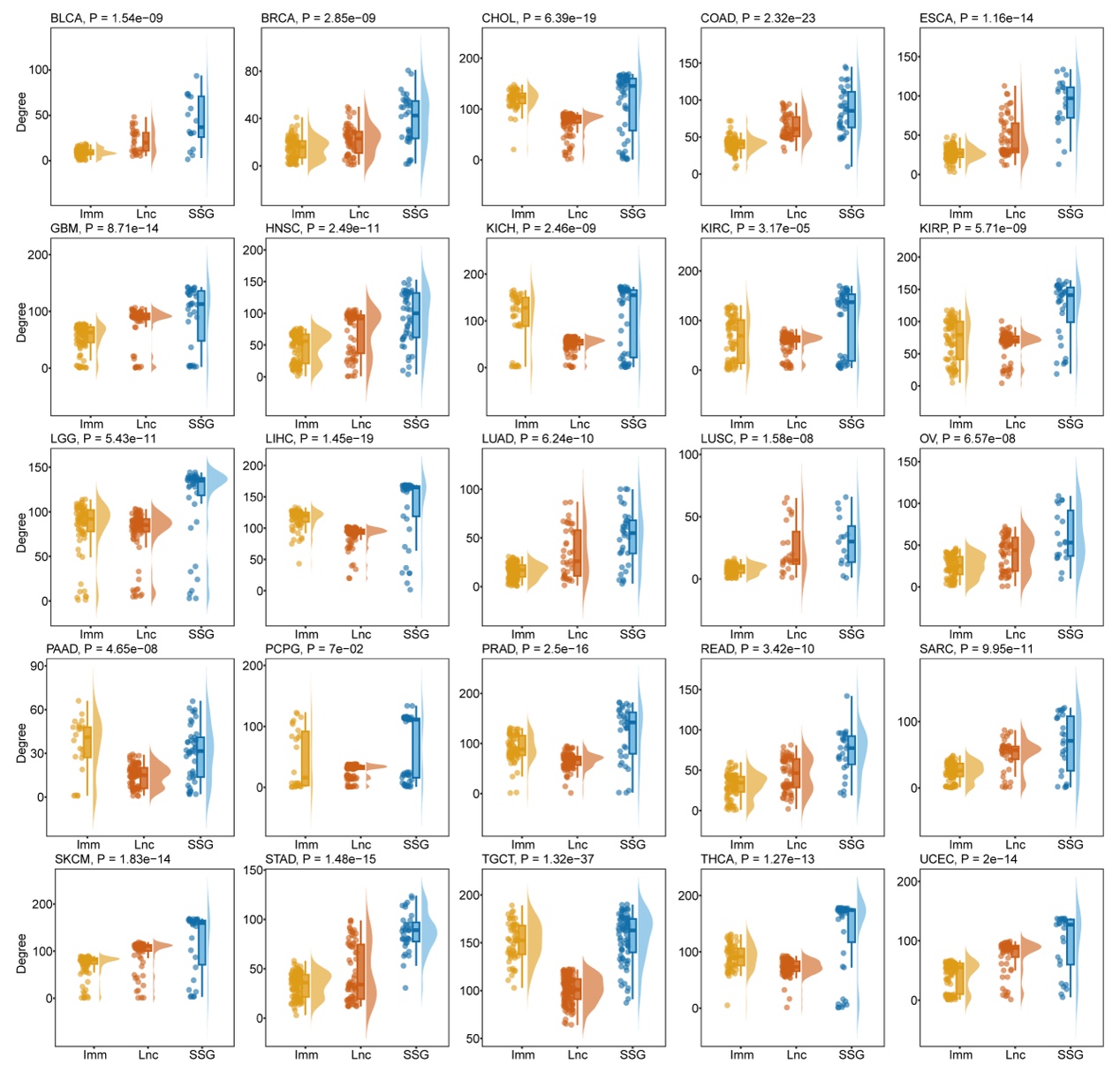


**Supplementary Figure 3.** Degree distributions of lncRNAs, SSGs, and immune genes in the STEM-LncCRT network based on their respective regulatory modes.


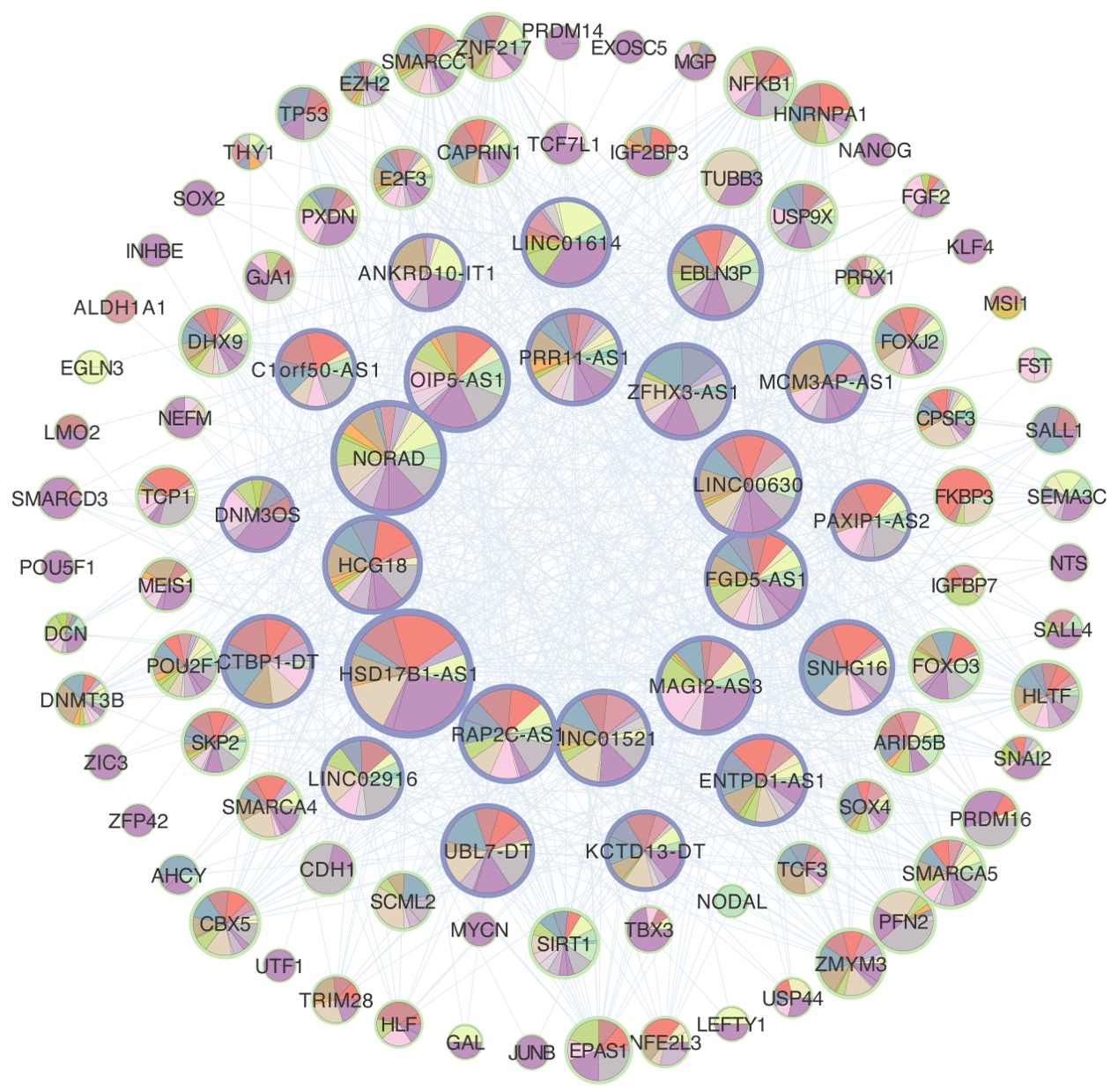


**Supplementary Figure 4.** Network of common lncRNAs. Green and purple nodes represent SSGs and common lncRNAs, respectively. The different colors within pie chart nodes indicate the presence of genes across various cancer types. The size of each node reflects the number of its connected edges.


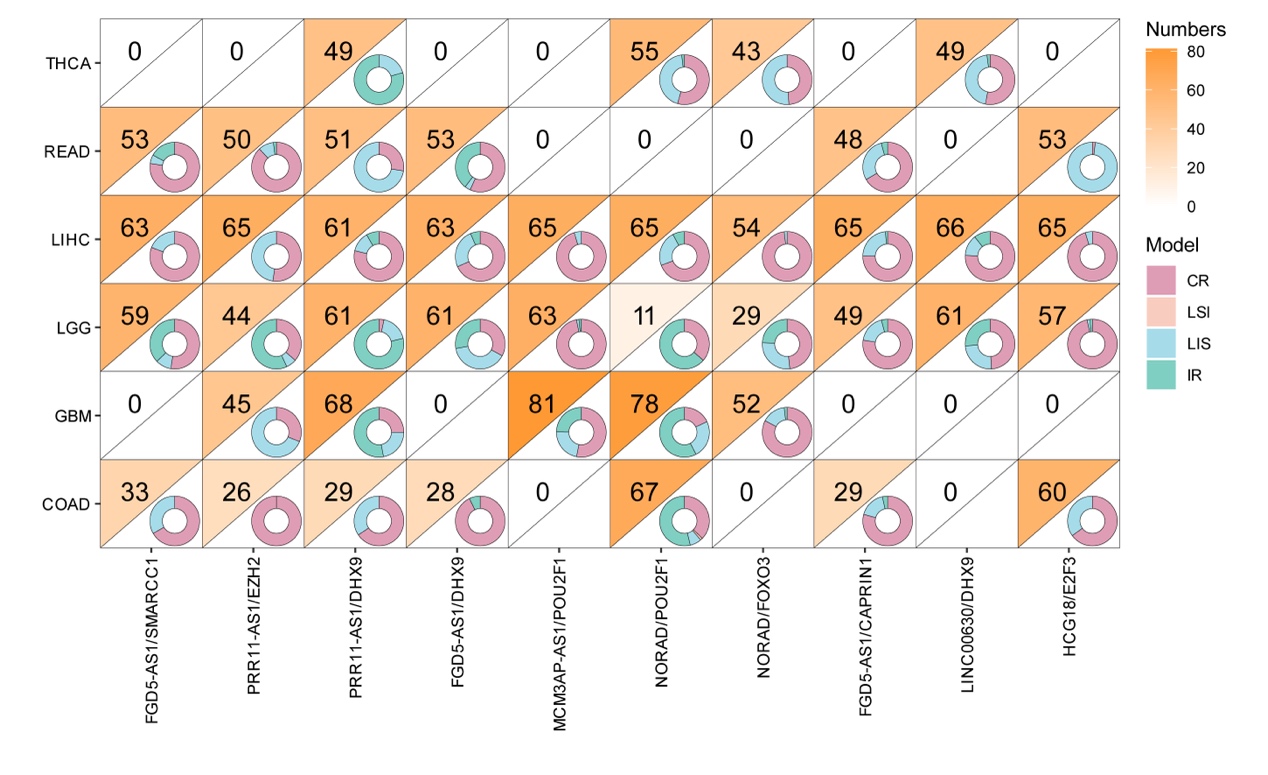
**Supplementary Figure 5.** The number of regulated immune genes and the corresponding regulatory patterns within variable-pattern lncRNA-SSG gene pairs across different cancer types.


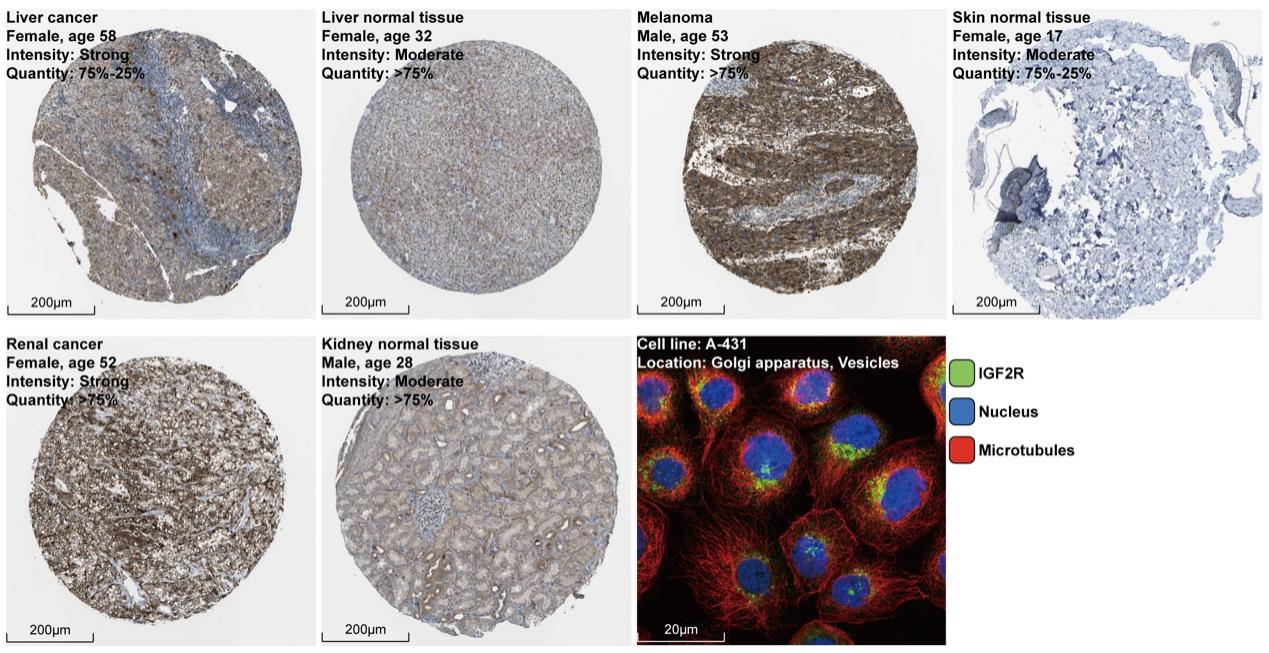


**Supplementary Figure 6.** Immunohistochemistry (IHC) staining of IGF2R in liver, skin, and renal tissues. Immunofluorescence (IF) staining of IGF2R in A-431 cells.


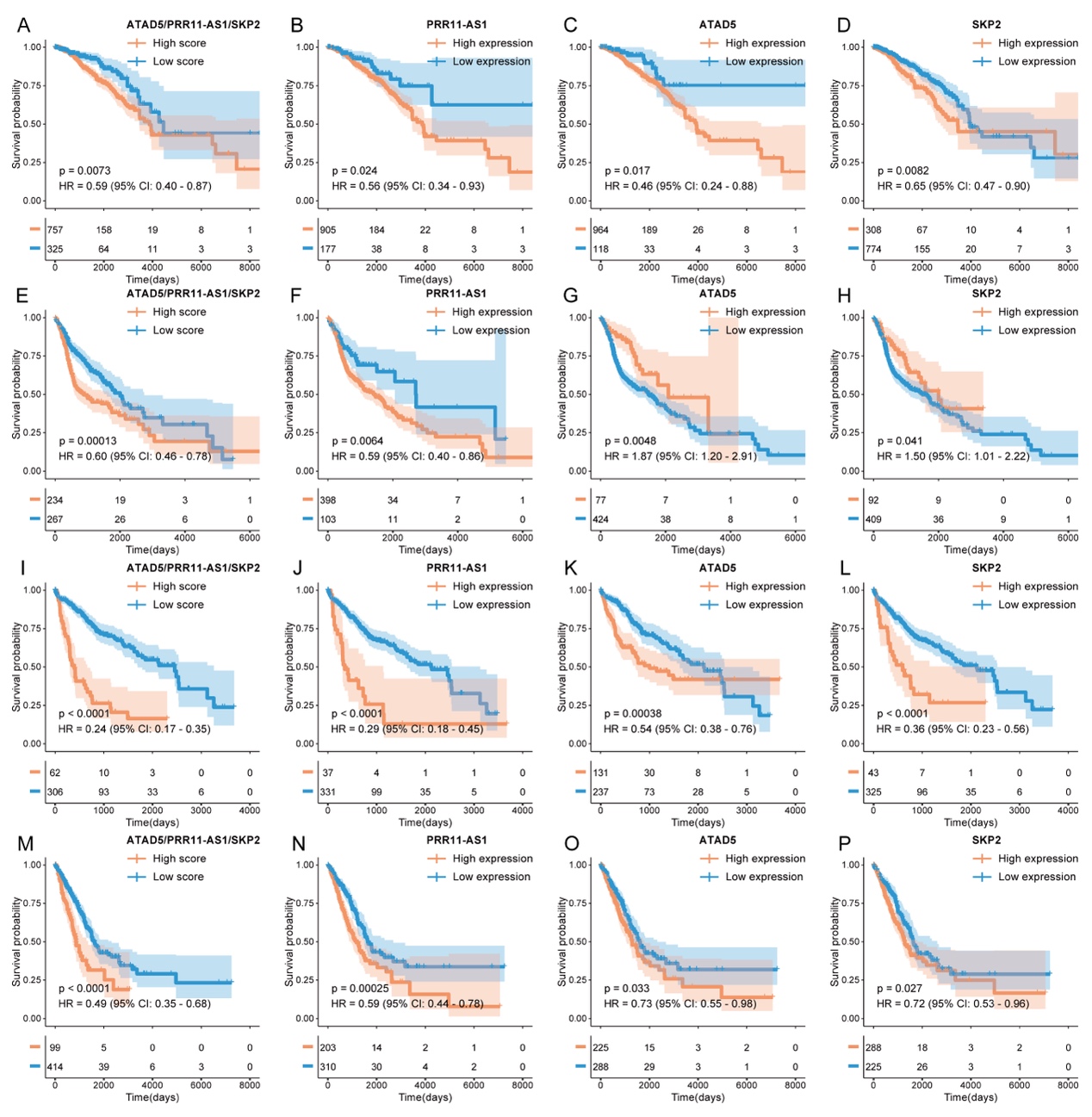


**Supplementary Figure 7.** Kaplan-Meier survival curves of ATAD5/PRR11-AS1/SKP2 and each individual gene in (A-D) BRCA, (E-H) HNSC, (I-L) LIHC, and (M-P) LUAD.


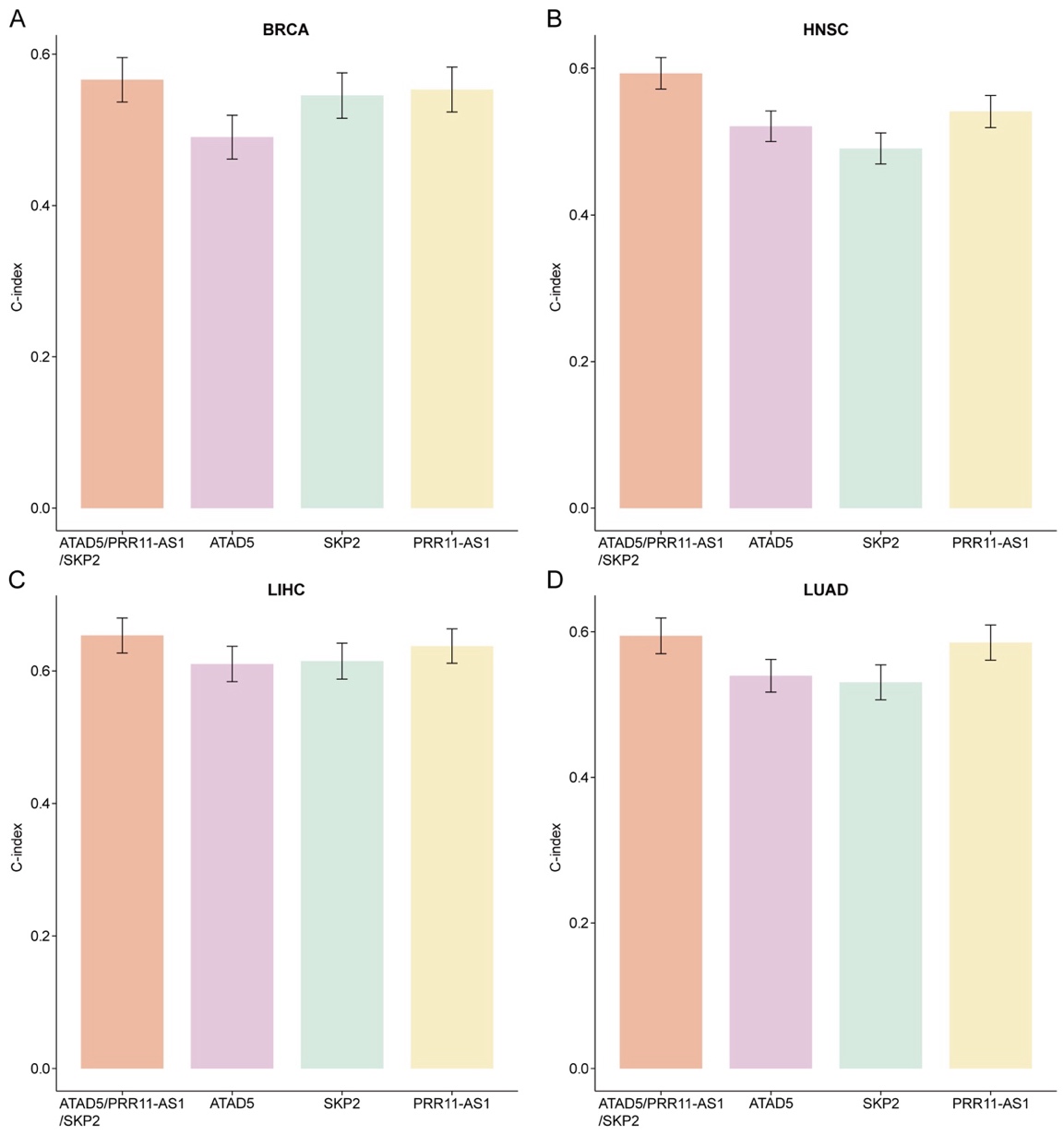


**Supplementary Figure 8.** C-index of ATAD5/PRR11-AS1/SKP2 and each individual gene in (A) BRCA, (B) HNSC, (C) LIHC, and (D) LUAD.


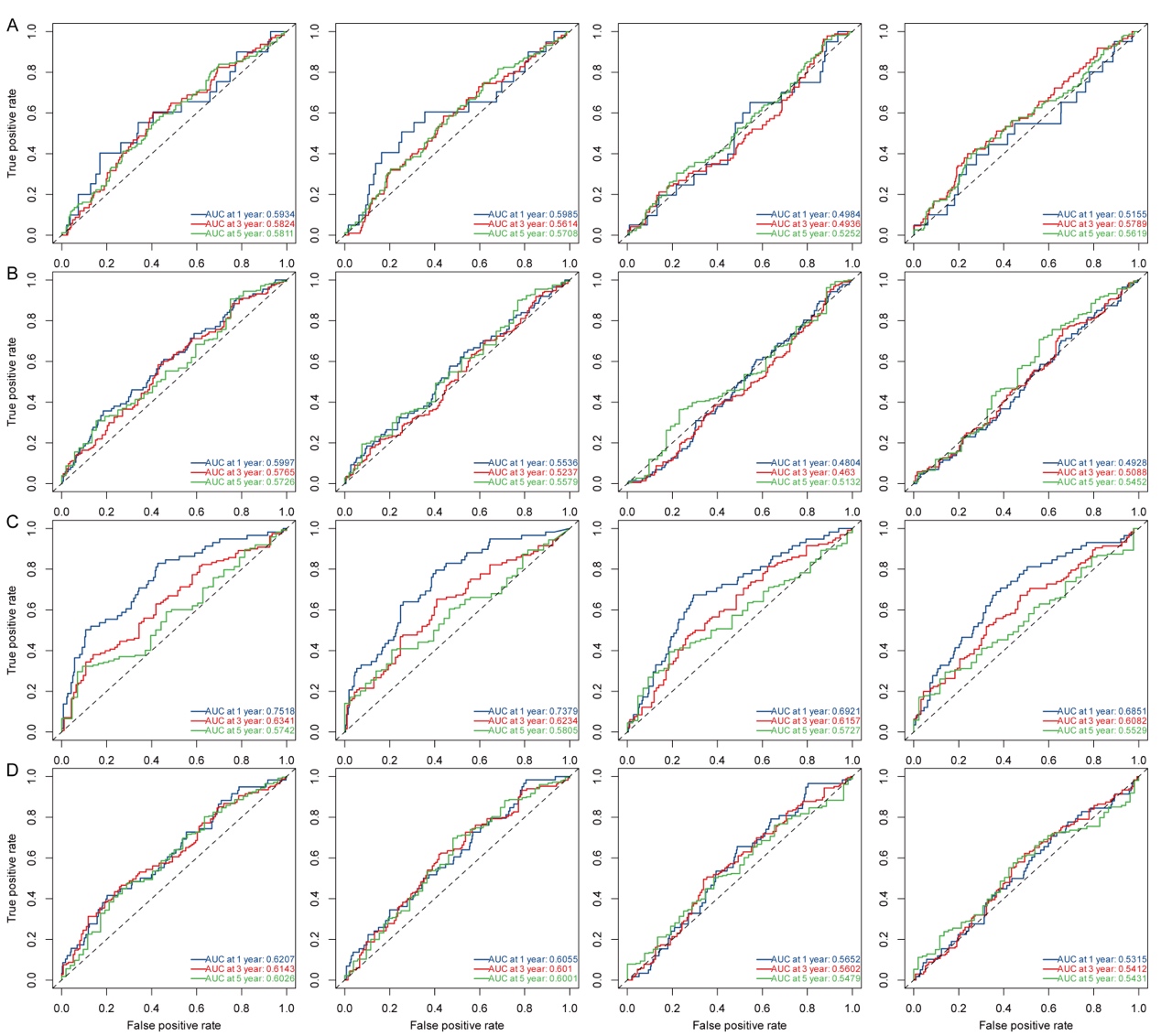


**Supplementary Figure 9.** Time-dependent ROC curves for 1-, 3-, and 5-year overall survival prediction based on ATAD5/PRR11-AS1/SKP2 and each individual gene (PRR11-AS1, ATAD5, and SKP2) in (A) BRCA, (B) HNSC, (C) LIHC, and (D) LUAD.


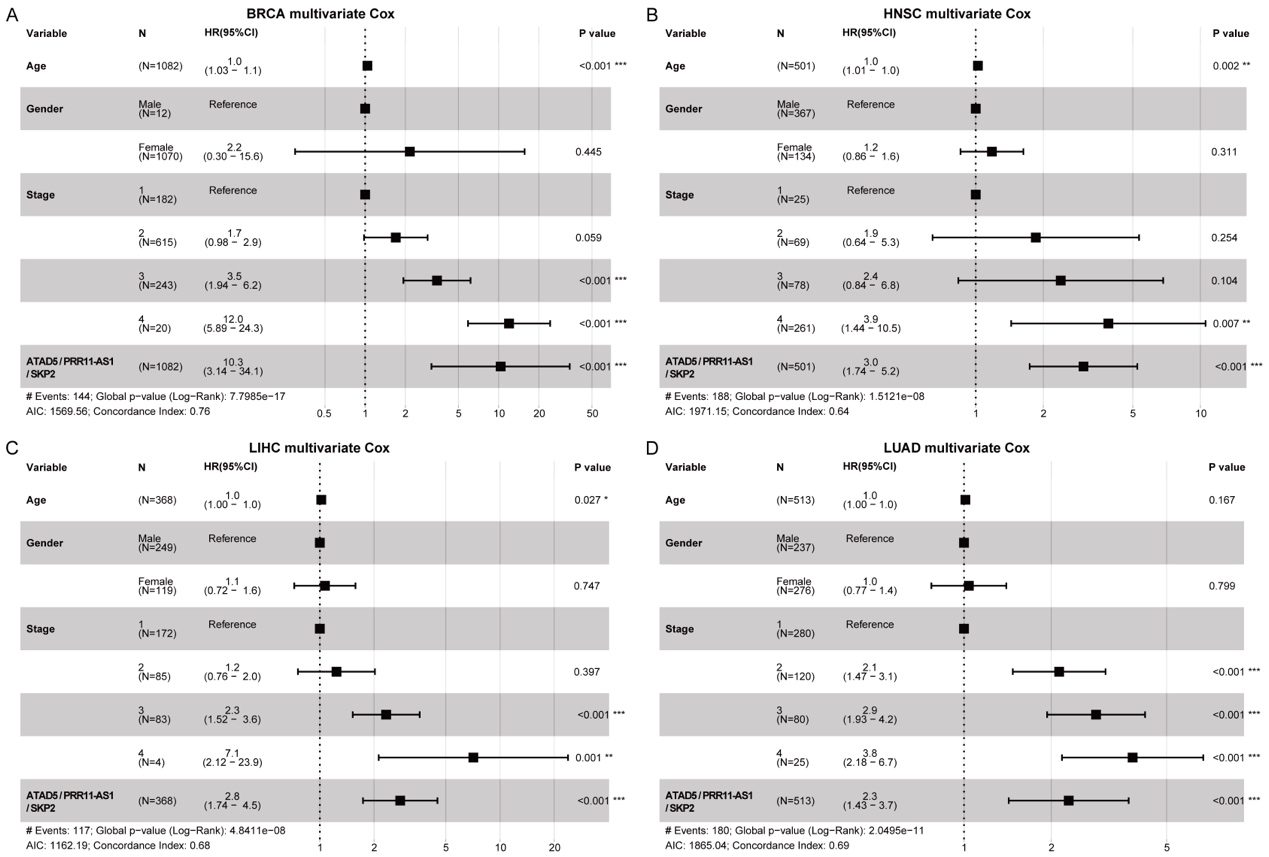


**Supplementary Figure 10.** Forest plot of the ATAD5/PRR11-AS1/SKP2 STEM-LncCRT score in (A) BRCA, (B) HNSC, (C) LIHC, and (D) LUAD.

**Supplementary Table 1.** Dataset sources and sample size statistics.

| Cancer Type | Detail | TCGA Tumor (N) | TCGA Normal (N) | GTEx (N) | Total | GTEx Normal Tissue |
| --- | --- | --- | --- | --- | --- | --- |
| BLCA | Bladder Urothelial Carcinoma | 411 | 19 | - | 430 | - |
| BRCA | Breast Invasive Carcinoma | 1104 | 113 | - | 1217 | - |
| CHOL | Cholangiocarcinoma | 36 | 9 | - | 45 | - |
| COAD | Colon Adenocarcinoma | 471 | 41 | - | 512 | - |
| ESCA | Esophageal Carcinoma | 162 | 11 | - | 173 | - |
| GBM | Glioblastoma Multiforme | 168 | 5 | 105 | 278 | Brain |
| HNSC | Head and Neck Squamous Cell Carcinoma | 502 | 44 | - | 546 | - |
| KICH | Kidney Chromophobe | 65 | 24 | - | 89 | - |
| KIRC | Kidney Renal Clear Cell Carcinoma | 535 | 72 | - | 607 | - |
| KIRP | Kidney Renal Papillary Cell Carcinoma | 289 | 32 | - | 321 | - |
| LGG | Brain Lower Grade Glioma | 529 | 0 | 105 | 634 | Brain |
| LIHC | Liver Hepatocellular Carcinoma | 374 | 50 | - | 424 | - |
| LUAD | Lung Adenocarcinoma | 526 | 59 | - | 585 | - |
| LUSC | Lung Squamous Cell Carcinoma | 501 | 49 | - | 550 | - |
| OV | Ovarian Serous Cystadenocarcinoma | 379 | 0 | 88 | 467 | Ovary |
| PAAD | Pancreatic Adenocarcinoma | 178 | 4 | 167 | 349 | Pancreas |
| PCPG | Pheochromocytoma and Paraganglioma | 183 | 3 | 128 | 314 | Adrenal Gland |
| PRAD | Prostate Adenocarcinoma | 499 | 52 | - | 551 | - |
| READ | Rectum Adenocarcinoma | 167 | 10 | - | 177 | - |
| SARC | Sarcoma | 263 | 2 | 396 | 661 | Muscle |
| SKCM | Skin Cutaneous Melanoma | 471 | 1 | 323 | 795 | Skin |
| STAD | Stomach Adenocarcinoma | 375 | 32 | - | 407 | - |
| TGCT | Testicular Germ Cell Tumors | 156 | 0 | 165 | 321 | Testis |
| THCA | Thyroid Carcinoma | 510 | 58 | - | 568 | - |
| UCEC | Uterine Corpus Endometrial Carcinoma | 548 | 35 | - | 583 | - |

**Supplementary Table 2.** The stemness-associated gene signatures curated in the StemChecker database.

| AASS | MAP7D3 | CER1 | RANBP5 | TBL1XR1 | MIF | LOC100190986 | DCTN5 | UTP14A | LOC284402 |
| --- | --- | --- | --- | --- | --- | --- | --- | --- | --- |
| ACTG1 | MAPK1 | CGI-115 | RARRES2 | TIMP3 | MLC1 | LOC149478 | DDX20 | UXS1 | LOC284551 |
| ADFP | MBD2 | CGI-30 | RBBP6 | TMEM5 | MMRN1 | LOC200030 | DDX23 | VAPB | LOC340515 |
| ADORA2B | MBL2 | CGI-48 | RBBP8 | TMEM9 | MN1 | LOC203427 | DDX39 | VIM | LOC386597 |
| APEH | MCAM | CHAF1A | RBM13 | TP53BP1 | MPDZ | LOC284184 | DDX41 | VLDLR | LOC388780 |
| ASH2L | MCM2 | CHAF1B | RBM14 | TPR | MPG | LOC645745 | DDX49 | VPS35 | LOC389257 |
| ATG4B | MCM3 | CHD1 | RBM28 | TRO | MPP5 | LOC646195 | DDX5 | VPS52 | LOC391405 |
| ATIC | MCM4 | CHEK1 | RBM39 | TSC22D1 | MPV17 | LOC728849 | DHCR7 | VRK3 | LOC400604 |
| ATP12A | MDK | CHGA | RBPSUH | TSPYL5 | MPZL1 | MAML2 | DHDDS | WDR20 | LOC400743 |
| ATP5B | MDN1 | CHORDC1 | RECQL | WHSC1 | MPZL2 | MANBA | DHRS3 | WDR23 | LPAR2 |
| ATP5C1 | MED22 | CHST4 | REPRIMO | ZAK | MREG | MAP1LC3B | DHX38 | WDR36 | LPAR5 |
| BCAT1 | MED28 | CHST7 | RET | ZMYM4 | MRPL57 | MAPBPIP | DIRC2 | WDR6 | LPPR2 |
| BFSP2 | METTL13 | CHST8 | RFC2 | ZNF135 | MRPS18A | MGP | DKK1 | WDR62 | LRRC33 |
| BFSP2-AS1 | MFAP2 | CISD1 | RFC4 | ZNF326 | MRPS18C | MMP14 | DMXL1 | WDR81 | LTA4H |
| C12orf5 | MGC10911 | CKAP2 | RFC5 | ZNF334 | MRPS27 | MSCP | DNAJB14 | WDTC1 | LVRN |
| C14orf15 | MGC24665 | CKAP5 | RGMA | ZNF410 | MRPS33 | NACA | DNAJC16 | WHSC1L1 | LY6D |
| C6orf211 | MGC3101 | CKS2 | RGS17 | ZNF470 | MSRA | NBPF14 | DNAJC8 | WWC3 | MAGEB4 |
| CALB1 | MIAT | CLDN6 | RIF1 | ZNF667 | MSRB2 | NDP | DNAJC9 | XAB1 | MAGEB5 |
| CAMP | MLEC | CLIC4 | RMI1 | ZNF70 | MTHFD2L | NDUFA6 | DNM2 | XAB2 | MAGEH1 |
| CAP1 | MLF2 | CNOT7 | RNF125 | ZNF85 | MTMR2 | NDUFB2 | DPAGT1 | ZCCHC3 | MAGT1 |
| CBR3 | MLLT11 | COBL | RNF138 | ABCA13 | MYCT1 | NEAT1 | DPM1 | ZCRB1 | MALL |
| CBS | MON1B | COCH | RNMT | AGBL3 | MYL6B | NFIB | DPYSL2 | ZEB2 | MAP2K7 |
| CCDC80 | MOSC1 | COL18A1 | ROBO1 | ANKRD28 | MYO15B | NID1 | DTNA | ZFAND6 | MAP3K1 |
| CCL26 | MRPL11 | COX6A1 | ROR1 | AREG | MYO18A | NKTR | DTX2 | ZFP36L1 | MASP1 |
| CD9 | MRPL12 | CPSF5 | RPL12 | ATP8B4 | MYOZ3 | NOPE | DUSP12 | ZNF174 | MBTD1 |
| CDC16 | MRPS16 | CRKL | RPL24 | B4GALT6 | NAALAD2 | NSMAF | DVL2 | ZNF185 | MCRS1 |
| CNBP | MRPS21 | CRLF1 | RPL39L | BEND4 | NBEA | P4HB | DYM | ZNF202 | MED13 |
| COQ3 | MRPS26 | CROP | RPL4 | BSPRY | NBL1 | PALM2-AKAP2 | ECOP | ZNF226 | MED13L |
| COX11 | MRPS34 | CSE1L | RPL7 | C1QTNF4 | NDUFAF1 | PANK3 | EIF2A | ZNF228 | MED19 |
| CR1 | MSH2 | CST1 | RPLP0 | CD34 | NDUFS4 | PCDH18 | EIF2AK3 | ZNF300 | MED24 |
| CSNK2B | MSL1 | CTBP2 | RPS2 | CDK6 | NEDD4 | PCDHB10 | EIF3D | ZNF434 | MGC10981 |
| CYTH1 | MT1M | CTCF | RPS24 | CHRDL1 | NEK3 | PCM1 | EIF3F | ZNF516 | MGC33407 |
| DMD | MTA3 | CTPS2 | RRM1 | CHST13 | NF1 | PDIA3 | EIF4ENIF1 | ZNF521 | MGC39821 |
| DNMT3B | MTF2 | CTSC | RRM2 | COL24A1 | NGFRAP1 | PERP | EIF4G2 | ZNF551 | MIRHG1 |
| DNMT3L | MTHFD2 | CTSL2 | RRS1 | CRHBP | NME7 | PET100 | ELL2 | ZNF664 | MMP15 |
| DPPA4 | MTL5 | CXCL12 | RUVBL1 | DDX17 | NOTCH1 | PKP1 | ENSA | ZNF701 | MMP24 |
| EEF2 | MTMR9 | CXXC6 | SACS | DEPTOR | NQO1 | PURB | ENTPD1 | ZNRF2 | MOCS1 |
| EIF3C | MYBL2 | CXorf15 | SALL1 | DLK1 | NR1H3 | RAB2B | EPS15L1 | ABTB2 | MR1 |
| EIF4A1 | MYCL1 | CYP26A1 | SALL3 | DPY19L2 | NREP | RABEP1 | ERH | ACD | MRPL51 |
| EPPK1 | MYCN | DARS2 | SALL4 | ETV6 | NT5C2 | RASA1 | ETNK1 | ARMCX5 | MS4A2 |
| ERVH-4 | MYH10 | DCC1 | SCLY | FAM65C | NUDT11 | RC3H2 | EXOC4 | ARPC5 | MS4A6E |
| ERVH48-1 | MYO10 | DDX18 | SEC22C | FLJ13197 | NUP62CL | REXO2 | EXPH5 | AUH | MVP |
| FABP5 | MYST1 | DDX21 | SEC5L1 | FLJ38379 | OCRL | RNF146 | FAM100A | C9orf97 | MYBPC2 |
| FBP1 | N4BP3 | DDX25 | SEMA3A | FLT3 | OR5T2 | RPL13A | FAM100B | CBY1 | MYBPHL |
| FKBP4 | NAP1L1 | DDX46 | SEMA5B | GATA2 | ORAI3 | RPL15 | FAM128A | CHST5 | MYO6 |
| FLVCR1 | NASP | DEK | SENP3 | GNA15 | OSBPL1A | RPL23A | FAM134C | DBR1 | NALCN |
| FRAT2 | NAV2 | DEPDC1B | SF3A1 | GPR126 | P15RS | RPL35A | FAM20C | DDX31 | NBEAL1 |
| GAL | NBPF15 | DHFR | SFRP1 | GUCY1A3 | P2RX1 | RPL36AL | FAM33A | ELAVL2 | NDUFA4L2 |
| GAPDH | NDUFA1 | DHFRL1 | SFRP2 | HBG1 | PAFAH1B3 | RPL39 | FAM35A | GADD45G | NDUFV1 |
| GDF3 | NDUFA13 | DHX29 | SFRS1 | HLF | PAIP1 | RPL7A | FAM54A | GPS1 | NELL2 |
| GLDC | NDUFA4 | DHX9 | SFRS11 | HOXA9 | PAQR3 | RPL8 | FAM63B | GRID2 | NEUROD2 |
| GZMB | NDUFS5 | DIAPH2 | SFRS18 | HTR1F | PCCA | RPLP1 | FAM92A1 | HHLA3 | NFKB1 |
| HESX1 | NEDD4L | DKC1 | SIL | IGLL1 | PDGFC | RPLP2 | FAM96A | HYPE | NFRKB |
| HLA-B | NEFL | DKFZP586L0724 | SIP | ITGA9 | PDGFD | RPP14 | FANCA | JUND | NHLRC1 |
| HLA-F | NELFB | DLAT | SIRT1 | KIAA0125 | PEX11A | RPS13 | FAT | KCNMB4 | NMUR2 |
| HM13 | NES | DLG7 | SKIL | KIT | PEX12 | RPS14 | FBLN1 | KIAA0174 | NPEPL1 |
| HMGN4 | NFE2L3 | DNA2L | SKP2 | LMO2 | PFN2 | RPS18 | FBXL11 | KIAA1279 | NRSN1 |
| HSD17B4 | NLE1 | DPP3 | SLC13A3 | LUC7L2 | PGAP1 | RPS19BP1 | FBXL14 | LSG1 | NUDT8 |
| HUS1 | NLGN4X | DPYSL3 | SLC1A5 | MBOAT1 | PGAP2 | RPS20 | FBXO31 | MAP3K3 | NXF1 |
| IFITM1 | NLN | DPYSL5 | SLC29A2 | MCTP2 | PGRMC1 | RPS25 | FBXW11 | MED11 | ODF2 |
| IFITM3 | NME2 | DRIM | SLC38A1 | MLLT3 | PHF16 | RPS29 | FEM1A | MEIS2 | ODF3L1 |
| INA | NOLA3 | DTYMK | SLC4A11 | MPL | PHLPP1 | RPS7 | FEM1C | NCBP1 | OMD |
| ITGB4BP | NOTCH3 | DUT | SLC5A6 | MPO | PHTF1 | RUFY3 | FHIT | NEFM | OPN1SW |
| JARID2 | NR2F6 | ECT2 | SLC6A8 | MPPED2 | PIGN | RUSC1 | FKSG24 | NPLOC4 | OR1D2 |
| LDHA | NRBP1 | EEF1A1 | SLC7A3 | MSI2 | PIGP | S100A2 | FLJ10769 | NR2F2 | OR2H1 |
| LRRC47 | NTHL1 | EEF1E1 | SLC7A8 | MSRB3 | PIK3R3 | SAT1 | FLJ25801 | PARD3 | OR5K4 |
| LST1 | NTS | EFCBP1 | SLD5 | NPR3 | PINLYP | SDCCAG1 | FLJ45455 | PIP5K1C | OR7D4 |
| MAN1C1 | NUAK1 | EFS | SMARCA5 | P2RY1 | PIP4K2B | SEC22B | FOXJ2 | PRKRIP1 | P2RX2 |
| MAP2K1 | NUCKS1 | EFTUD1 | SMC2L1 | PAN3 | PLA2G4A | SEC61G | FOXO3 | RAD51C | P2RY11 |
| MCL1 | NUDT1 | EGFL4 | SMC6L1 | PDZD2 | PLAG1 | SERPINA3 | FUS | RDH10 | P2RY12 |
| MEOX2 | NUDT15 | EGLN3 | SNRPA | PGDS | PLAGL1 | SERPINB5 | FXYD5 | RFNG | PARP3 |
| MGST1 | NXN | EIF1AX | SNRPA1 | PTPRD | PLCB1 | SFPQ | FZD10 | SFXN1 | PCDHA1 |
| MID1 | OGDHL | EIF2S1 | SNRPD2 | RAPGEF2 | PLCB4 | SFRS15 | GALK2 | STMN2 | PDE2A |
| MRPL15 | OIP5-AS1 | EIF3S10 | SNRPD3 | RRAGD | PLCH1 | SHQ1 | GANC | TBC1D22 | PDZD11 |
| MRPL18 | OLFM1 | EIF3S3 | SNTB1 | SAMD13 | PLK2 | SLC24A2 | GAS2L1 | TCF4 | PITX1 |
| MRPL37 | ORC1L | EIF3S6 | SORD | SCD5 | PLOD2 | SLC30A7 | GATAD1 | TJP3 | PITX3 |
| MRPS23 | OSBPL6 | EIF3S9 | SOX11 | SCN3A | PLSCR4 | SLC41A2 | GBF1 | TMEM30A | PKLR |
| MRS2L | OTX2 | EIF4E | SOX3 | SLC22A16 | PLXND1 | SLC7A6 | GGA1 | TRA2A | PLCL1 |
| MT1A | PAI-RBP1 | EIF5B | SPAG5 | SLC25A27 | PML | SMARCA4 | GIYD2 | TRPS1 | PNPO |
| MT1E | PAIP2B | ELOVL6 | SPRY1 | SLC2A5 | POLD2 | SNED1 | GLA | WDFY3 | PODNL1 |
| MT1F | PAK1 | ENAH | SRP72 | SLITRK4 | POLR3D | SNHG6 | GLG1 | WDR70 | POLH |
| MT1G | PARP1 | ENPP1 | SRRM1 | SOCS2 | PON2 | SNRPE | GLT8D3 | ZHX2 | POLR2E |
| MT1H | PBX1 | EOMES | SS18L2 | SPINK2 | PPFIBP1 | SOCS3 | GNA13 | ZKSCAN5 | PPAPDC2 |
| MT1X | PBX3 | EPB41L4B | SSB | SSBP2 | PPIP5K1 | SOD2 | GNG10 | ZMPSTE2 | PPOX |
| MT2A | PCDH1 | EPRS | SSBP1 | STARD9 | PPM1F | SPTBN1 | GNG12 | ABLIM1 | PPP1R15B |
| MTHFD1 | PCNA | ERBP | STC1 | STT3B | PPP1R26 | STX2 | GOLGA4 | ACIN1 | PRDM13 |
| NALP2 | PCTK1 | EXOSC2 | STRBP | TARDBP | PPP2R3A | TBCA | GOLIM4 | ACOX1 | PRDM9 |
| NANOG | PDGFA | EXOSC5 | SUPT16H | TCTEX1D1 | PRDX4 | TBRG1 | GORASP2 | AKT1S1 | PRDX6 |
| NARS | PDPN | EXOSC9 | SWAP70 | VWDE | PRKCSH | TICAM2 | GPC6 | ARID4B | PRELP |
| NCEH1 | PDZK4 | EXTL2 | SYNCOILIN | WDR49 | PRMT5 | TIMM23 | GPR108 | ARL4D | PRG3 |
| NDUFA9 | PELI1 | FAM131B | SYNGR3 | ZNF711 | PRRG1 | TM9SF2 | GRHL2 | ARRDC3 | PROP1 |
| NDUFAB1 | PFAS | FAM29A | Spc25 | ZNRF1 | PRSS2 | TMEM49 | GRIPAP1 | ASNA1 | PSMD2 |
| NDUFB11 | PFKFB4 | FAM72A | TA-LRRP | AAR2 | PRSS3 | TMSB10 | GRPEL2 | ATF3 | PSPH |
| NLRP7 | PGD | FANCG | TA-PP2C | ABCC1 | PSMA6 | TNRC6A | GSK3A | AZI1 | PSTPIP2 |
| NUDC | PGM5 | FANCL | TACC3 | ABCG1 | PTPLA | TPM4 | GSTCD | BDH2 | PXN |
| PAICS | PHB | FBXO5 | TAF4B | ABHD10 | PTRF | TRAPPC5 | GSTT2 | BTG1 | RAD17 |
| PDCL3 | PHB2 | FCHO1 | TAF9L | ACACA | PUS7L | TRIM27 | GTF3C4 | C14orf108 | RAD9B |
| PFN1 | PHF17 | FEN1 | TARS | ACADM | QARS | TRIO | GTPBP1 | C14orf159 | RALGDS |
| PGK1 | PIF1 | FEZ1 | TDGF1 | ACN9 | RAB13 | TROVE2 | GTPBP3 | C16orf80 | RAP1GAP |
| PIM2 | PIM1 | FGF13 | TEBP | ACOT13 | RAB38 | TRPC1 | GYPC | C19orf43 | RASEF |
| PITPNC1 | PIN4 | FIGNL1 | TFAM | ACP6 | RAB40B | TRPM7 | H2AFZ | C1orf174 | RBM10 |
| PLS3 | PIPOX | FKBP1B | TFRC | ACTR8 | RABGAP1 | TUBB2C | HBP1 | C1orf55 | RBM17 |
| PNRC2 | PKD1-like | FKBP3 | TGIF | ADA | RALA | TUBB3 | HCFC1R1 | C21orf66 | REXO1 |
| POU5F1 | PKIB | FKBP5 | THOC1 | ADCY3 | RBM12B | TUG1 | HECTD2 | C6orf130 | RHOA |
| PRDM14 | PLCXD1 | FKSG14 | THRAP6 | ADO | RBM7 | TXNIP | HEXIM2 | CCDC123 | RICTOR |
| PRDX1 | PLD2 | FLJ10036 | THY28 | ADPRM | RCBTB1 | UBA52 | HIGD2A | CLP1 | RIN1 |
| PRDX3 | PLN | FLJ10330 | TIA1 | AHCY | RCN1 | UBE2K | HIST3H2A | COPB1 | RNF216 |
| PRIM2A | PMAIP1 | FLJ10378 | TIMP4 | AHCYL2 | RDX | UBL3 | HMBOX1 | COPS7A | RNF40 |
| PROM1 | PNPLA4 | FLJ10407 | TIPIN | AK2 | REC8 | UBL5 | HMG20A | CREB3L4 | RPESP |
| PRPS2 | POLR2H | FLJ10534 | TK1 | AKR1A1 | REPIN1 | UBR5 | HNRNPA2B1 | CRKRS | RRAS |
| PSMB3 | POLR3G | FLJ10808 | TKT | AKR1C3 | RERE | VCAM1 | HNRPH2 | CXCL5 | RUFY4 |
| PSMD10 | POLR3K | FLJ11184 | TMSL8 | AKR7A2 | RHOBTB1 | WASF3 | HNRPUL1 | CYB5R2 | SAMD7 |
| PSME3 | POP7 | FLJ12519 | TNNT1 | ALCAM | RHPN1-AS1 | WFS1 | HSPA5 | EAPP | SCGB1A1 |
| RAD51AP1 | POSTN | FLJ12650 | TNPO1 | ALDH1A1 | RICS | XRN2 | HSPBAP1 | EIF2C2 | SENP6 |
| RBM3 | PPAT | FLJ13842 | TNPO3 | ALG3 | RIPK2 | YAF2 | HSPC171 | ETHE1 | SERPINB10 |
| RPL18A | PPIA | FLJ13909 | TNRC9 | ALG9 | RMND1 | YEATS2 | ID2 | EXOC5 | SERPINB2 |
| RPL28 | PPIH | FLJ20485 | TOP2A | AMACR | RNF144A | ZBTB1 | IDH3A | FAM76B | SERTAD2 |
| RPL6 | PPM1H | FLJ20641 | TPD52 | ANAPC13 | RNF2 | ZCCHC9 | IER5L | FANCC | SETD1B |
| RRAS2 | PPP1R1A | FLJ20674 | TPM3 | ANGEL1 | RNF8 | ZDHHC6 | IFI16 | FBXL19 | SF3A3 |
| RTCB | PPP2R1B | FLJ21148 | TPT | ANKMY2 | ROBO3 | ADAMTS3 | IFT52 | FBXO16 | SFXN3 |
| SEMA6A | PPT2 | FLJ21901 | TPX2 | ANKRD15 | RPGRIP1L | AMER2 | IL1RAPL1 | FIP1L1 | SLC16A3 |
| SFRS7 | PRDX2 | FLJ21908 | TRA16 | ANKRD46 | RPL10L | AQP4 | ILF2 | FLJ20309 | SLC17A9 |
| SLC25A16 | PRDX5 | FLJ21918 | TRERF1 | ANKRD6 | RPL41 | ARX | INF2 | FLJ40125 | SLC25A23 |
| SMS | PRELID1 | FLJ21924 | TRIM14 | ANKS1A | RSL1D1 | ASCL1 | ING1 | FOXP1 | SLC25A42 |
| SRSF3 | PRICKLE1 | FLJ22662 | TRIM2 | AP4M1 | RUNX1-IT1 | C1orf61 | INPP4A | FTL | SLC2A12 |
| SUCLA2 | PRKAB2 | FLJ30046 | TRIM22 | APIP | SATL1 | COLGALT2 | INTS12 | FZD1 | SLC2A9 |
| TBRG4 | PRKX | FLJ31434 | TRIM24 | APTX | SCARB2 | CPE | IQCK | FZD3 | SLC46A3 |
| TEAD2 | PRMT1 | FLJ40432 | TRIM36 | ARHGAP22 | SCARF1 | CSPG5 | ISCU | GDAP1 | SMCR5 |
| TEAD4 | PRPS1 | FNBP3 | TRIP8 | ARHGAP29 | SCCPDH | CTNND2 | ISOC2 | GGPS1 | SMPD1 |
| TERF1 | PRR5 | FOXN3 | TSC | ARHGAP33 | SCD | DCX | IWS1 | GRK6 | SMU1 |
| TMPO | PRSS8 | FOXO1 | TTF2 | ARHGAP5 | SCMH1 | DNER | JMJD1A | GSK3B | SNAP23 |
| TPI1 | PSMA2 | FRAS1 | TTK | ARHGEF17 | SCML2 | EDNRB | JOSD1 | H3F3B | SOAT2 |
| TPST2 | PSMB2 | FRSB | TUBA1 | ARL3 | SCN9A | FABP7 | JUB | HDAC9 | SORDL |
| UBE2C | PSMB6 | FSD1 | TUBB | ARMCX1 | SDCCAG8 | FAM107A | JUN | HDGF2 | SOX14 |
| UPP1 | PSRC1 | FXR1 | TUBB6 | ASAP2 | SEC63 | FOXG1 | KATNB1 | HEPH | SPI1 |
| UQCRH | PTAR1 | G3BP1 | TXNDC | ASB13 | SEPT11 | GAS1 | KCTD15 | HIF1AN | SPINK7 |
| UTP6 | PTBP1 | G3BP2 | UACA | ASB9 | SERF1B | GPM6A | KCTD2 | HMOX1 | STK10 |
| VSNL1 | PTK7 | GABRA5 | UBA2 | ASPH | SERPINB6 | GRIA2 | KDELR3 | HNRNPC | STK4 |
| WARS | PTMA | GAD1 | UBE1C | ATP1B1 | SERPINE2 | HS6ST2 | KDR | HUWE1 | SUB1 |
| WBP11 | PTMS | GAJ | UGCGL1 | ATP5G2 | SERPINI2 | ITGB8 | KHDRBS1 | IDH3G | SUV39H2 |
| WDHD1 | PTPRF | GALNT1 | UGT8 | ATP5SL | SH3BGR | LHX2 | KHSRP | IK | SVIL |
| XPO1 | PTPRN | GALNT12 | UNC5D | ATP6V0A2 | SH3BP4 | LRRC3B | KIAA0241 | INHBA | SYF2 |
| YARS2 | PTPRS | GART | UNG | ATP7B | SHFM1 | MAP2 | KIAA0368 | KIAA0247 | SYNCRIP |
| ZBTB2 | PTPRU | GCLC | USO1 | ATP9A | SIRT5 | MEGF10 | KIAA0391 | KIAA0280 | SYTL4 |
| ZNF138 | PTPRZ1 | GEMIN4 | USP1 | ATXN7L3B | SLC19A2 | NRXN1 | KIAA0652 | KIAA0319L | TAAR5 |
| ZNF195 | PTTG1 | GJA7 | USP28 | AVEN | SLC25A13 | PAX6 | KIAA1143 | KIAA0495 | TADA2B |
| ZNF432 | PUNC | GLMN | USP32 | B3GNT1 | SLC35F2 | PTN | KIAA1217 | LASP1 | TAF2 |
| ZNF588 | PVRL2 | GLS2 | USP44 | B3GNT2 | SLC35G2 | RFX4 | KIF9 | LPHN1 | TAF7 |
| ZNF589 | PXDN | GMFB | USP46 | BACE1 | SLC39A14 | SEMA6D | KLF5 | MAPRE2 | TAS2R45 |
| ZNF684 | RAB34 | GNAL | USP9X | BAHCC1 | SLC39A8 | SLC10A4 | KLF7 | MAST1 | TAX1BP1 |
| ZNF79 | RAB3B | GNG4 | USP9Y | BBS10 | SMAD1 | SLC1A2 | KLHL18 | MDC1 | TBC1D10A |
| ZNF816 | RALY | GNL2 | UTF1 | BCKDHB | SMARCA1 | SLC1A3 | KLHL4 | MED17 | TCL1A |
| ABCB7 | RAMP | GNL3 | VANGL2 | BCL11A | SMARCA2 | SP8 | KLHL5 | MEX3C | TCP1 |
| ACHE | RAMP2 | GNPTAB | VBP1 | BCL2L2 | SMARCC1 | SPARCL1 | KPNA3 | MOSPD3 | TFDP2 |
| ACSS3 | RASGEF1A | GPR | VENTX2 | BCORL1 | SMYD3 | ST6GALNAC5 | LAMA4 | MPHOSPH1 | TIFA |
| ACVR2B | RASL11B | GPR160 | VRK1 | BEX4 | SNHG20 | ZIC1 | LARGE | MPND | TIGD3 |
| ADD2 | RBM47 | GPR23 | WBSCR16 | BHLHB9 | SOCS5 | ACTRT3 | LCMT2 | MRPL43 | TMEM14B |
| AK3L1 | RBMX | GPR64 | WBSCR17 | BLMH | SOX12 | AFTPH | LEPROTL1 | MRPL47 | TMEM14C |
| AKAP2 | RBPMS | GRB14 | WDR12 | BTBD3 | SPAG16 | ALX1 | LHPP | MRPS11 | TMEM204 |
| ALDH7A1 | RBPMS2 | GRPR | WDR3 | BZW2 | SPATS2 | APLP1 | LIN37 | MRPS31 | TOB2 |
| ALKBH7 | REST | GRTP1 | WDR4 | C10orf95 | SPATS2L | ARHGDIB | LMCD1 | MTSS1 | TRAIP |
| ANKS6 | RFC3 | GSG2 | WDSOF1 | C11orf67 | SPG20 | ARMC9 | LRAT | MYNN | TRAM2 |
| AP1M2 | RHNO1 | GTF2E1 | XRCC5 | C11orf71 | SPHAR | ARMCX2 | LRFN3 | MYO9A | TRPA1 |
| AP1S2 | RNASEH2A | GULP1 | YBX2 | C11orf95 | SPIDR | ARRB2 | LRIG3 | NAG | TRYX3 |
| APOB | RNF12 | GYG2 | YTHDF2 | C14orf1 | SPIN1 | ASCC3 | LRP2 | NCDN | TSPAN4 |
| APOC1 | RNF149 | GYLTL1B | ZBTB3 | C15orf29 | SPIN2B | ATP11C | LRP3 | NDUFA2 | TTC23L |
| APOE | RNF175 | H2AFX | ZFP42 | C16orf62 | SPP1 | BBS9 | LRRC49 | NDUFB5 | TTC39B |
| ARHGEF16 | RNF41 | HA-1 | ZIC3 | C1QTNF3 | SPRED2 | BOD1 | LSM3 | NIT1 | TTC4 |
| ARID3B | RNU3IP2 | HAS3 | ZNF117 | C1orf216 | SPTLC2 | C10orf128 | LSMD1 | NKIRAS1 | UBE2V1 |
| ARL2 | RPA3 | HDAC2 | ZNF146 | C1orf54 | SRBD1 | C14orf119 | LYPD1 | NUDT4 | UBXN8 |
| ARL6IP | RPL22 | HIST1H2BG | ZNF198 | C21orf59 | SRI | C14orf166 | MAN2C1 | NUFIP2 | UFSP1 |
| ATP5G3 | RPS10 | HIST1H4K | ZNF257 | C2CD2 | ST3GAL6 | C4orf52 | MAP3K11 | OGT | ULK2 |
| ATP5H | RPS19 | HLTF | ZNF267 | C2orf44 | STAC | C5orf56 | MAP3K12 | PCF11 | UPP2 |
| ATPBD4 | RPS21 | HMBS | ZNF273 | C2orf68 | STAT5A | CCDC90B | MAPK14 | PCSK5 | VARS |
| ATPIF1 | RPS3 | HMGB2 | ZNF331 | C4orf27 | STAU2 | CCDC99 | MAPK8 | PER2 | VWF |
| ATXN10 | RPS6KA1 | HMGN2 | ZNF43 | C5orf42 | STK32B | CCR1 | MCC | PGM2L1 | WAC |
| AURKB | RPSA | HMMR | ZNF447 | C6orf108 | STMN1 | CD53 | MCTS1 | POLDIP3 | WBP2 |
| AUTS2 | RYBP | HNRNPA1 | ZNF505 | C6orf162 | STON1 | CDC123 | MDH1 | POLE3 | WDR53 |
| AXIN2 | SALL2 | HNRNPL | ZNF518 | CACNB2 | STX3 | CENPI | MED12 | POLR3E | WDR82 |
| B3GAT1 | SAPCD2 | HOMER1 | ZNF553 | CALCRL | SUOX | CMBL | MED23 | PPAPDC1B | XRCC1 |
| B4GALNT3 | SBK1 | HPCL2 | ZNF567 | CAND2 | SYNGR1 | CPSF3 | MED25 | PPP1R10 | YIPF7 |
| B4GALNT4 | SC5DL | HPRP8BP | ZNF614 | CANT1 | SYPL1 | CTSH | MEF2A | PPP1R11 | ZC3H18 |
| BAK1 | SCAMP5 | HPS3 | ZNF638 | CAPRIN1 | TAOK3 | CTSS | METTL7A | PPP1R15A | ZDHHC15 |
| BASP1 | SCG3 | HRMT1L3 | ZNF770 | CASP2 | TARBP1 | DACT1 | MICA | PRCC | ZDHHC20 |
| BAX | SCG5 | HSD11B2 | ZRF1 | CBFA2T3 | TBC1D8B | DCUN1D5 | MIR16 | PRKAR1A | ZFP36 |
| BBC3 | SCGB3A2 | HSP90B1 | ZSCAN10 | CCDC121 | TCEAL1 | DDX1 | MKKS | PRPSAP1 | ZFP64 |
| BCL9 | SCHIP1 | HSPA14 | GSH1 | CCDC6 | TCEAL4 | DHX15 | MKRN1 | PTPN3 | ZIC4 |
| BICD1 | SCNN1A | HSPA4 | IMP2 | CCNB1IP1 | TCF12 | DIAPH3 | MLH1 | RAB4B | ZNF136 |
| BIRC5 | SCOTIN | HSPA8 | LDHB | CCNG1 | TCF7L2 | DIMT1 | MMP2 | RBBP9 | ZNF197 |
| BMP7 | SDC4 | HSPA9B | TUBB4 | CCT2 | TEK | DPH3 | MMP9 | RBM23 | ZNF35 |
| BMPR1A | SEC61A2 | HSPC111 | DUSP6 | CCT6B | TEX30 | DTD1 | MOBKL2B | RCOR3 | ZNF354A |
| BOLA2 | SEMA4C | HSPD1 | FAM162A | CD164 | TFEC | EIF2AK4 | MOBP | RDH11 | ZNF513 |
| BTC | SEPHS1 | HT017 | ANKRD10-IT1 | CD99 | TFPI | EIF2B3 | MORF4L1 | RNF14 | ZNF546 |
| BTF3L4 | SEPN1 | HTR1D | B3GALT5 | CDC42BPA | THG1L | ERCC2 | MORF4L2 | RNUXA | ZNF786 |
| C10orf35 | SERPINB8 | Hs.105196 | C12orf35 | CDCP1 | THSD7A | FAM118B | MOV10 | RPL30 | ZNHIT3 |
| C12orf45 | SERPINB9 | Hs.173497 | CBL | CDH2 | TIE1 | FANCB | MPP6 | RPL36A | ACVR1 |
| C16orf34 | SERPINH1 | Hs.178761 | ESRG | CDK2AP1 | TIMM9 | FBXO22 | MRPL27 | RPL9 | ACVR1B |
| C1GALT1 | SESN2 | Hs.40154 | FAM199X | CENPO | TM4SF1 | FKSG49 | MSC | RSF1 | ACVR1C |
| C1orf106 | SET | Hs.60548 | FOXH1 | CEP170 | TM7SF3 | FYB | MSL3L1 | RTN2 | ACVR2A |
| C1orf108 | SEZ6L2 | ID1 | G2E3 | CEP83 | TMED3 | FZD2 | MST150 | SAP30 | AKT1 |
| C20orf129 | SFTPC | IDH1 | GATA6 | CERS6 | TMEM135 | GIMAP1 | MTM1 | SGTA | AKT2 |
| C8orf42 | SH2D2A | IFI30 | HAND1 | CETN3 | TMEM159 | GIMAP5 | MTMR1 | SH3GL3 | AKT3 |
| CACHD1 | SHF | IGF2BP3 | IL6ST | CFH | TMEM220 | GIMAP6 | MUS81 | SLC38A2 | AXIN1 |
| CACNA2D2 | SIAT1 | IGFBP2 | LIFR | CLCC1 | TMEM254 | GIMAP7 | MYO3A | SMG7 | BMI1 |
| CALU | SILV | IL17RD | LINC00545 | CLNS1A | TMEM38B | GIMAP8 | MYST3 | SNAPC1 | BMP4 |
| CAP2 | SKA3 | ILF3 | MUM1 | COA3 | TMEM45A | GPX8 | NADSYN1 | SNAPC3 | BMPR1B |
| CAPG | SLC16A1 | IMPA1 | NANOS1 | COL4A5 | TMEM97 | HDX | NAT12 | SORT1 | BMPR2 |
| CAPZA2 | SLC25A11 | IMPDH2 | NT5DC1 | COL5A1 | TNFRSF1A | HIST1H4C | NBR1 | SPCS2 | CTNNB1 |
| CASP3 | SLC25A29 | INDO | PUM1 | COPG2IT1 | TNFRSF21 | HLA-DMB | NCOR1 | SPIRE1 | DLX5 |
| CCBL1 | SLC25A6 | IPO7 | PUM2 | COQ7 | TNFSF4 | HLA-DPA1 | NDNL2 | STAP2 | DUSP9 |
| CCL2 | SLC29A1 | IPO9 | RFWD3 | COX15 | TNRC18 | HLA-DQA1 | NDUFB3 | STCH | DVL1 |
| CCNC | SLC2A1 | IQGAP2 | SOX1 | CPT1A | TNS3 | HLA-DRB5 | NEBL | TAF15 | ESRRB |
| CCND2 | SLC2A3 | ITGA6 | SOX17 | CRELD1 | TOM1L1 | HTR7 | NFAT5 | TBC1D17 | ESX1 |
| CCNE1 | SLC39A1 | ITGB1BP3 | STAT3 | CRIM1 | TOMM34 | IL10RA | NFS1 | TNFAIP2 | FZD4 |
| CCNF | SLC39A10 | ITGB5 | TCF3 | CRYGD | TOX | INHBE | NIF3L1 | TNFSF5IP1 | FZD9 |
| CD200 | SLC3A2 | ITPR3 | TRIM71 | CSTF2T | TP53I3 | JUNB | NIP30 | TRIT1 | GRB2 |
| CD24 | SLC6A6 | ITSN1 | TRMT1 | CTDSPL | TPD52L1 | KIF7 | NMT1 | TXNDC12 | HNF1A |
| CD276 | SLCO1A2 | JMJD1C | TRMT13 | CTNNA2 | TPRKB | LOC400931 | NOL6 | UBR1 | HRAS |
| CDC14B | SNHG16 | JMY | UFM1 | CXCL10 | TRAF3IP2 | LOC90768 | NPAS2 | USP25 | ID3 |
| CDC25A | SNORD22 | KAL1 | ACAD8 | CXCL3 | TRAPPC6A | MALAT1 | NSUN3 | USP49 | ID4 |
| CDC6 | SNRPB | KCNK5 | ADH5 | CXCR7 | TRIM16 | MLL3 | NVL | VASP | IGF1 |
| CDCA5 | SNRPF | KCNN2 | ALDOC | CYFIP1 | TRIM32 | MLLT6 | ODF2L | VAT1 | INHBB |
| CDCA7 | SNRPN | KCNS3 | AMOTL2 | CYP51A1 | TRIM5 | MMADHC | ORC6L | WDFY2 | JAK2 |
| CDH1 | SNX24 | KCTD14 | ANP32E | CYTL1 | TRIM68 | MRPL3 | OSGEP | XRRA1 | JAK3 |
| CDH3 | SNX27 | KIAA0007 | ATP5J | DACH1 | TRMT112 | MS4A7 | OSR1 | ZCCHC14 | KLF4 |
| CDIP1 | SOCS1 | KIAA0020 | ATP5O | DAPK1 | TRPV1 | MTHFD1L | OTUB1 | ZFYVE19 | LHX5 |
| CDR1 | SORL1 | KIAA0101 | AURKA | DBN1 | TSGA14 | MXI1 | OTUD7B | ZNF140 | MAP2K2 |
| CECR1 | SOX13 | KIAA0114 | BANF1 | DCTPP1 | TSPAN2 | NMNAT2 | OXA1L | ZNF335 | MAPK11 |
| CHCHD1 | SOX2 | KIAA0153 | BTF3 | DDAH1 | TSPAN3 | OSTC | P4HA1 | ZNF428 | MAPK12 |
| CHEK2 | SOX4 | KIAA0179 | C11orf48 | DDAH2 | TSPYL4 | PDE4C | PALB2 | ZNF646 | MAPK3 |
| CIB2 | SPINT1 | KIAA0483 | C2orf47 | DDT | TTC28 | PDHB | PAPSS2 | ZNF668 | MYF5 |
| CITED4 | SPINT2 | KIAA0523 | CCNB2 | DENR | TTC30A | PECAM1 | PARG | ZNF677 | NEUROG1 |
| CKMT1 | SPON1 | KIAA0650 | CCND1 | DHCR24 | TUBGCP4 | PILRB | PARP8 | ZSCAN2 | NRAS |
| CKS1B | SPRY2 | KIAA0922 | CDC34 | DHRS4 | TXN | PLAA | PCBP1 | AADACL2 | ONECUT1 |
| CLDN10 | SPRY4 | KIAA1274 | CDCA3 | DKFZP434C153 | TXN2 | PLRG1 | PCNXL3 | ABCG8 | OTX1 |
| CLDN7 | SRSF10 | KIAA1287 | CDCA8 | DKFZP564C152 | TXNRD3 | PTPRC | PCTK2 | ABP1 | PCGF1 |
| CNKSR1 | SSBP3 | KIAA1553 | CDK4 | DLG4 | UBE2E1 | RAD1 | PDCL | ABTB1 | PCGF2 |
| CNTNAP2 | SSR4 | KIAA1712 | CDKN1C | DNAJC12 | UBE2E3 | RARS | PEA15 | ACADS | PCGF3 |
| COA1 | ST8SIA1 | KIAA1982 | CDKN3 | DNAL4 | UCK2 | RARS2 | PERLD1 | ACBD3 | PCGF5 |
| COX6B1 | STC2 | KIF1A | CLPP | DOCK1 | UFC1 | RCSD1 | PEX1 | ACPL2 | PCGF6 |
| COX7C | STK17A | KIF20A | COX4NB | DOK4 | UNC119B | RGCC | PFTK1 | ACSS1 | PIK3CA |
| CP | STK26 | KIF23 | COX5B | DPF2 | UNC13B | RIOK2 | PHF23 | ACTL6B | PIK3CD |
| CPA3 | STK35 | KIF2C | CYCS | DPY19L4 | UQCRB | RSAD2 | PHF8 | ACTR6 | PIK3CG |
| CPXM1 | SUV420H1 | KIF4A | DAP3 | DRAM1 | USE1 | SAMM50 | PHTF2 | ADAM7 | PIK3R1 |
| CRABP1 | SYT6 | KIF5C | DBF4 | DST | USP22 | SEC11A | PICALM | ADAMTSL1 | PIK3R5 |
| CRABP2 | TAC3 | KIFC2 | DNMT1 | DUSP10 | USP39 | SKIV2L2 | PIGL | ADIPOR1 | POU5F1B |
| CRISPLD1 | TACSTD1 | KLHL7 | E2F3 | DUSP14 | VAV3 | SLC24A1 | PIH1D1 | ADRBK1 | RAF1 |
| CRMP1 | TALDO1 | KLKB1 | EBNA1BP2 | DYNC2LI1 | VGLL4 | SLC25A3 | PIK3R2 | AHCTF1 | SETDB1 |
| CRSP2 | TBC1D16 | KNTC1 | ECHS1 | E2F6 | VPS8 | SMARCC2 | PKIG | AIPL1 | SMAD2 |
| CRYM | TBCD | KNTC2 | EIF2S2 | ECI2 | VRK2 | SNX8 | PKMYT1 | ALDH1L2 | SMAD4 |
| CSRP2 | TCEA1 | KRR1 | EIF3S2 | EFHC2 | WASF1 | SOHLH2 | PLEKHA3 | ALDH5A1 | SMAD9 |
| CTH | TCF7L1 | KRT18 | EIF3S6IP | EGR3 | WBP5 | TBCE | PLEKHG3 | ALKBH8 | TBX3 |
| CTNNA1 | TD-60 | LARP7 | EIF4B | EHD2 | WDR5B | TEX10 | PNMA1 | ANGPT4 | WNT1 |
| CTNNBIP1 | TDG | LCK | ENO1 | EHHADH | WDR60 | TM2D2 | POLL | ANGPTL6 | WNT10A |
| CXADR | TFAP2C | LDB2 | ERP29 | EI24 | WWOX | TNFSF10 | POLR1A | ANKRD31 | WNT10B |
| CYP2S1 | TGFB2 | LECT1 | ETFA | EID1 | XBP1 | TRNT1 | POU4F1 | ANXA4 | WNT11 |
| DBC1 | TGIF2 | LEFTY1 | EXO1 | EIF2D | YWHAE | TRPC4 | PPIL3 | APC | WNT16 |
| DCAMKL1 | THOP1 | LGALS1 | FAM136A | ELK3 | ZBED4 | TSHZ2 | PPP1R2 | APLF | WNT2 |
| DCP2 | THY1 | LGALS8 | FARSA | ELMO1 | ZBED8 | UBXN2A | PPP2R1A | ARFIP2 | WNT3 |
| DHX33 | TIMM17B | LGR4 | FBL | EMCN | ZDHHC4 | UGGT2 | PPP2R3C | ARL4C | WNT4 |
| DIDO1 | TLE1 | LIG1 | FDPS | EMID1 | ZFAND1 | XPOT | PPP2R5C | ARL5C | WNT5B |
| DLG3 | TMEFF1 | LIN28 | FH | EMP1 | ZKSCAN1 | XXYLT1 | PRCP | ARS2 | WNT6 |
| DNAH11 | TMEM132A | LINGO1 | GARS | EPHB4 | ZKSCAN4 | ZBTB20 | PREPL | ASAP1 | WNT7A |
| DNAJB6 | TMEM169 | LISCH7 | GEMIN6 | ERCC1 | ZMIZ1 | ATAD2 | PRKCDBP | ASTN2 | WNT7B |
| DNMT3A | TMEM261 | LNK | GLO1 | ERCC8 | ZNF167 | CEP55 | PRPF19 | ATOH8 | WNT8A |
| DPY30 | TMEM50B | LOC112885 | GNA14 | EREG | ZNF184 | ESPL1 | PRPF31 | ATP1A3 | WNT8B |
| DSG2 | TMSB15B | LOC134492 | GSPT2 | ERG | ZNF189 | FOXM1 | PRPF38A | BAIAP2L1 | WNT9A |
| DSP | TNC | LOC138255 | GTSE1 | ERGIC3 | ZNF193 | GTPBP4 | PRPF39 | BAT4 | WNT9B |
| DUSP16 | TNFRSF8 | LOC144097 | HADH | ERLIN1 | ZNF219 | ISG20L2 | PRR11 | BCL6B | ZFHX3 |
| E2F5 | TNNI3 | LOC157627 | HAT1 | ERLIN2 | ZNF248 | KIF14 | PSEN2 | BDP1 | EPAS1 |
| EDIL3 | TOMM40 | LOC90806 | HDAC1 | ERMP1 | ZNF444 | KIF15 | PSENEN | BEST1 | GSC |
| EFNA2 | TPM1 | LPAAT-e | HN1 | ESD | ZNF529 | MELK | PSMA1 | C10orf40 | HIF3A |
| EFR3B | TRAF4 | LRIG1 | HNRPK | ESR1 | ZNF606 | NCAPG | PSMB1 | C12orf68 | NR5A1 |
| EHD3 | TRNP1 | LRP8 | IARS | ESRRG | ZNF609 | PBK | PSMB4 | C13orf35 | ACTL6A |
| EIF1 | TSPAN33 | LSM5 | KIF11 | ETS2 | ZNF629 | PDSS1 | PSMC2 | C16orf13 | ARID1A |
| EIF2S3 | TSPAN6 | LSM6 | KIF22 | ETV5 | ZNF654 | PRC1 | PSMD9 | C16orf81 | ATAD3B |
| EIF3S12 | TSR2 | LUC7L | LSM10 | EXDL2 | ZNF671 | TTC13 | PTCD3 | C17orf74 | ATG7 |
| EIF4EBP1 | TTC19 | LYAR | LSM2 | EXOSC4 | ZNF710 | WDR67 | PTPN1 | C19orf24 | BATF |
| ENO2 | TUBB2 | MAD2L2 | LSM4 | EXT2 | ZNF835 | ZWINT | PWP1 | C19orf68 | CDC73 |
| EPB41 | UCHL1 | MAL2 | MAD2L1 | F2R | ZSCAN31 | ACAA2 | PXMP3 | C1orf157 | CDCA4 |
| EPHA1 | UGP2 | MAN2A1 | MAPK13 | FADS1 | ADAM28 | ALDH3B1 | R3HDM1 | C20orf95 | CDK2 |
| ERBB2 | UNC13A | MAP4K1 | MID1IP1 | FAHD2A | EFHC1 | AMT | RAB15 | C21orf128 | CDKN1A |
| ERBB3 | UNC5B | MAP7 | MRPL13 | FAIM | EGFL7 | ATP6V0E | RAB17 | C22orf15 | CRTAC1 |
| ERRFI1 | UNQ470 | MARS | MRPL16 | FAM115A | MGC4655 | ATP6V1D | RAB3GAP2 | C2orf69 | CTR9 |
| ETV1 | VASH2 | MARVELD2 | MRPL39 | FAM117A | NPDC1 | CDO1 | RAB5A | C6orf163 | CXCR4 |
| ETV4 | VAV2 | MARVELD3 | MRPL4 | FAM124B | THSD1 | CLN5 | RAB5B | C6orf195 | DCSTAMP |
| EXOSC7 | VCAN | MASTL | MRPS17 | FAM179B | ADAM12 | CUGBP1 | RABL3 | CA9 | DGCR8 |
| EYA4 | VPS25 | MAT2A | MRPS18B | FAM216A | ADAMTS1 | GCH1 | RABL4 | CAPN2 | DPF1 |
| F2RL1 | WBP1 | MAT2B | MRPS2 | FANCF | ADAMTS5 | HFE | RAD23A | CASC1 | DPF3 |
| FALZ | WDR72 | MATK | MRPS28 | FARP1 | ALDH1A3 | LPP | RAI1 | CCDC103 | EIF5A |
| FAM124A | WDR9 | MATR3 | MRPS36 | FAT4 | CDH11 | NUP210 | RANBP10 | CCDC130 | EIF5A2 |
| FAM126A | WDR92 | MCM10 | MRTO4 | FBN1 | CEMIP | OVOL2 | RANGAP1 | CCDC33 | EIF5AL1 |
| FAM46B | WFDC2 | MCM5 | NCAPD2 | FBXO11 | COL12A1 | PPM1E | RAP1A | CCDC60 | ELAVL1 |
| FAM60A | WNK2 | MCM6 | NCBP2 | FGGY | COL1A1 | PRNP | RARB | CCDC74A | ELL3 |
| FAM72B | WRNIP1 | MCM7 | NDUFA11 | FHL1 | COL1A2 | RAB25 | RASGRF2 | CCDC74B | ERAS |
| FAM83H | YARS | MEST | NDUFB10 | FKBP9 | COL3A1 | RND2 | RBBP4 | CCNK | ERF |
| FAM84B | YWHAG | METAP1 | NDUFB7 | FLJ11710 | COL6A1 | RNF44 | RBM22 | CD59 | EZH1 |
| FAPP2 | ZBTB39 | METTL3 | NDUFB8 | FLNB | COL6A2 | SEMG1 | RBM4 | CDC37L1 | EZH2 |
| FAXC | ZD52F10 | METTL8 | NDUFS2 | FLT1 | COL6A3 | TXNRD1 | RBM9 | CDC42 | FES |
| FBXL10 | ZDHHC18 | MFAP3L | NEK2 | FOCAD | CYP1B1 | AASDH | RBP1 | CDH18 | FOXO4 |
| FBXO2 | ZDHHC5 | MFGE8 | NHP2 | FPGS | DCN | ABCF2 | RFX1 | CDH19 | HMCES |
| FCER1G | ZFP82 | MGC10993 | NIP7 | FRMD4B | ENPP2 | ABHD11 | RGS10 | CDH23 | HMHB1 |
| FGF12 | ZFR2 | MGC13096 | NME4 | FSCN1 | FOXD1 | ABHD2 | RIC8B | CDX2 | HMSD |
| FGF18 | ZIC2 | MGC19531 | NONO | FSTL1 | FST | ACAT2 | RIPK1 | CERCAM | IL11 |
| FGF2 | ZIK1 | MGC2574 | NOP2 | FXYD6 | GALNT5 | ACO2 | RNF121 | CGGBP1 | IL3 |
| FGFR1 | ZMYM3 | MGC2603 | NSBP1 | FZD6 | GLT8D2 | ACOT8 | RNF24 | CHCHD10 | KMT2E |
| FGFR2 | ZMYND8 | MGC42530 | NT5DC2 | G6PC3 | GREM1 | ACSL4 | RNF25 | CHRNE | LEO1 |
| FGFR3 | ZNF217 | MGC45866 | PA2G4 | GAB2 | HAS1 | ACTR1A | RNF31 | CHST1 | LGR5 |
| FGFR4 | ZNF232 | MGC72075 | PABPC1 | GABPB1 | HAS2 | ACTR1B | RP11-529I10 | CLASP1 | LGR6 |
| FLAD1 | ZNF281 | MGC8407 | PDHA1 | GALNT2 | HMCN1 | ADAL | RPL17 | CLCN1 | MACF1 |
| FLJ10156 | ZNF286A | MGC8685 | PDIA4 | GALNT7 | HOXC6 | ADAR | RPL21 | CLRN3 | METTL14 |
| FLJ10700 | ZNF398 | MGEA5 | PHC1 | GANAB | IGFBP3 | ADD3 | RPL32 | CLTA | MSI1 |
| FLJ10884 | ZNF423 | MICB | PHF5A | GAR1 | IL1R1 | ADRBK2 | RPP25 | CNOT1 | MST1R |
| FLJ12484 | ZNF473 | MIZ1 | PLK1 | GATM | ISLR | AHSA1 | RPS26 | COL11A1 | MYO1G |
| FLJ12644 | ZNF496 | MKI67IP | POLR2F | GBE1 | ITGBL1 | ALKBH1 | RPS27A | COL4A4 | NANOS2 |
| FLJ20105 | ZNF578 | MLF1IP | PPM1G | GCNT1 | LOXL1 | AMIGO2 | RPS3A | COPS2 | NLK |
| FLJ20171 | ZNF697 | MLLT10 | PPP4C | GFRA1 | NID2 | AMOTL1 | RRN3 | COPS4 | NR0B1 |
| FLJ21168 | ZNF829 | MLSTD1 | PSMA5 | GGCT | OLFML3 | ANKRD1 | RSPRY1 | CORT | NR2C1 |
| FLJ30594 | ZNRF3 | MMP1 | PSMA7 | GGH | PAPPA | ANKRD49 | RSRC2 | COX6A2 | NR2C2 |
| FLJ34633 | ZSCAN26 | MMP25 | PSMB5 | GLCE | PDGFRA | ANP32A | RUNX1T1 | CPT2 | PADI4 |
| FLVCR1-AS1 | AARS | MRE11A | PSMD14 | GMPR2 | PDZRN3 | ANP32B | RYR3 | CREBL2 | PAF1 |
| FNBP1L | ABCE1 | MRPL1 | RACGAP1 | GNAI1 | PENK | ANXA1 | S100A11 | CTDP1 | PHF10 |
| FOXA3 | ABHD9 | MRPL42 | RAD18 | GNG11 | PPAP2B | AP1G1 | SART3 | CTNNA3 | PHF19 |
| FOXD3 | ACTA1 | MRPS30 | RAD23B | GPATCH2L | PPP4R2 | AP2A1 | SASH1 | CTSE | PIWIL2 |
| FUBP1 | ACTC | MRPS9 | RCC1 | GPATCH4 | PRR16 | AP3B1 | SAT2 | CXorf59 | PRMT7 |
| FZD7 | ACTN3 | MSH6 | RCN2 | GPR124 | PRRX1 | APEX2 | SAV1 | CYBA | PSCA |
| FZD8 | ADAM19 | MTB | RNPS1 | GPR56 | RARRES1 | APH1A | SCAF1 | CYP11B2 | PSMD11 |
| GABARAPL1 | ADAM23 | MTIF2 | RPA2 | GPSM2 | SLC16A4 | APLP2 | SCNM1 | CYP21A2 | PTPRQ |
| GABRA1 | ADCY2 | MYB | RPL10A | GRB10 | SNAI2 | APOA2 | SEC24A | DARS | RNF43 |
| GABRB3 | ADM | MYC | RPL13 | GRHPR | SOX9 | APOM | SECISBP2 | DBX2 | RTF1 |
| GAP43 | ADNP | MYEF2 | RPL27A | GSTM5 | SULF1 | AQP2 | SEMA3C | DCTN2 | RUNX2 |
| GARNL4 | ADSL | MYL7 | RPP40 | GTF2H5 | TAGLN | ARF4 | SEMA4F | DDIT3 | SMARCB1 |
| GCHFR | AER61 | MYO5C | RPS12 | GTF2I | THBS2 | ARHGAP1 | SENP2 | DEFB126 | SMARCD1 |
| GCSAML-AS1 | AF020591 | MYOHD1 | RPS16 | GUCY1B3 | TRHDE | ARHGAP11A | SERINC3 | DERL2 | SMARCD3 |
| GCSH | AFP | MYRIP | RPS23 | H1FX | TWIST1 | ARID1B | SERP1 | DFFA | SMARCE1 |
| GFPT2 | AGL | MYST2 | RPS27 | H2AFJ | VGLL3 | ARID5B | SERPINA1 | DLGAP1 | STK3 |
| GGT5 | AGPS | NAP1L2 | RPS5 | H2AFV | WNT5A | ARIH1 | SF3B1 | DNAH8 | TBX6 |
| GGT7 | AK3 | NAP1L3 | RPS8 | H2AFY | ABHD6 | ARMC6 | SF3B5 | DNAJC10 | TMEM18 |
| GID8 | AKAP1 | NARG1 | RUVBL2 | HCG8 | ACER3 | ASAH1 | SFI1 | DOC2A | TMSB4X |
| GJA1 | ALPL | NARG2 | SARS | HDGFRP3 | AK1 | ASAM | SFRS14 | DRG2 | TP63 |
| GLI1 | AMD1 | NBR2 | SDHC | HHAT | AKR1C1 | ASB1 | SFRS4 | E4F1 | USP36 |
| GLI2 | AMMECR1 | NCL | SDHD | HHEX | AKR1C2 | ASCC2 | SFT2D3 | ECE2 | WDR61 |
| GLI3 | ANAPC1 | NCOA6IP | SEPHS2 | HIBCH | ANKH | ASXL1 | SGK | EFCAB4A | WWP2 |
| GLT1D1 | ANK2 | NCRMS | SIP1 | HIST1H1D | APP | ATF4 | SGK3 | EIF2B1 | WWTR1 |
| GMNN | ANKHD1 | NFYB | SLC25A5 | HIST1H2AE | ARHGEF2 | ATG4C | SGMS1 | EIF2B2 | YLPM1 |
| GNPDA1 | ANKRD10 | NIPBL | SMC4 | HIST1H2AG | ARMC8 | ATP5F1 | SIN3B | EIF2B4 | ZHX3 |
| GPC3 | APBB2 | NIPSNAP1 | SNRPD1 | HIST1H2BD | ATP5E | ATP6V1A | SLAIN2 | EMP3 | AATF |
| GPC4 | APEX1 | NLGN4Y | SNX5 | HIST1H2BF | ATP5I | ATP6V1G | SLC1A1 | EMX1 | AIMP2 |
| GPM6B | API5 | NME1 | SQLE | HIST1H2BI | ATP5L | B2M | SLC36A4 | ENPP7 | ANXA2 |
| GPR19 | APPBP1 | NMU | SS18 | HIST1H2BK | BAG3 | B3GALT4 | SLC39A9 | EP300 | ANXA3 |
| GPR27 | ARF3 | NOC3L | STIP1 | HIST1H3D | BGN | BAG5 | SLC40A1 | ETF1 | ARL4A |
| GPRC5B | ARFGEF1 | NODAL | STOML2 | HIST1H4E | C11orf31 | BAMBI | SLC44A1 | ETFDH | BCL2 |
| GRAMD1A | ARHGAP19 | NOL5A | SUMO1 | HIST2H2 | C16orf69 | BAT3 | SLC4A1A | EYA3 | BIK |
| GRB7 | ARHGAP8 | NOLC1 | TCF19 | HIST2H2AA3 | C17orf85 | BCKDHA | SLC7A11 | FAM114A1 | BYSL |
| GSTP1 | ARL8 | NOVA1 | TCOF1 | HIST2H2AA4 | C19orf2 | BCL9L | SLC7A5 | FAM120B | CASP6 |
| GUCA1A | ARNTL2 | NP | THOC3 | HMGA2 | C20orf30 | BCLAF1 | SMAD3 | FAM127B | CDC25C |
| H19 | ASC | NPM1 | TIMM13 | HMGCS1 | C22orf13 | BLCAP | SMARCA | FAM169B | CDK7 |
| H3F3A | ASE-1 | NPM3 | TIMM44 | HOPX | C2orf24 | BMP2 | SMG5 | FAM19A1 | COIL |
| HAPLN4 | ASML3B | NPTX2 | TIMM8A | HOXA10 | C8orf59 | BNIP1 | SNRP70 | FAM45A | COPS3 |
| HDHD1 | ASPM | NR6A1 | TIMM8B | HOXA2 | C9orf16 | BSCL2 | SNUPN | FAM82A1 | COPS6 |
| HELLS | ASS | NSD1 | TP53 | HOXA4 | C9orf64 | C10orf26 | SNX1 | FBXL6 | DAXX |
| HEY2 | ATE1 | NUBP1 | TRIP13 | HOXA5 | CA12 | C11orf1 | SON | FBXW2 | DAZAP1 |
| HIC2 | ATP1A2 | NUDT5 | TRIP6 | HOXB5 | CALD1 | C12orf60 | SORBS1 | FGL1 | DDX11 |
| HINT1 | ATP1B3 | NUFIP1 | U2AF1 | HPGD | CALM1 | C14orf122 | SP2 | FIBIN | DIAPH1 |
| HIP1 | ATXN7L1 | NUP107 | UBE2G1 | HPRT1 | CCDC72 | C14orf133 | SPARC | FIG4 | DPPA2 |
| HK1 | B3GNT7 | NUP160 | UBE2V2 | HSD17B12 | CCL5 | C14orf138 | SPIRE2 | FLJ10404 | ELAC2 |
| HMGA1 | BAT1 | NUP205 | UGDH | HSD17B8 | CCNL1 | C15orf24 | SPRED1 | FLJ22447 | ERCC5 |
| HMGB3 | BCCIP | NUP35 | UQCR | HSDL2 | CDC42SE2 | C16orf63 | ST3GAL2 | FLJ41047 | EWSR1 |
| HMGCR | BCL2L12 | NUP37 | WDR77 | HSP90AB1 | CDC5L | C16orf72 | STK11IP | FLJ44450 | EXOSC3 |
| HNRPAB | BCOR | NUP54 | WEE1 | HSPB1 | CEBPD | C18orf37 | STK36 | FLJ44881 | EXOSC8 |
| HNRPU | BLM | NUP62 | YAP1 | HYI | CHN1 | C19orf42 | STRN | FLJ45244 | GADD45A |
| HOOK1 | BM039 | NUP88 | YY1 | HYMAI | CLTB | C19orf54 | STXBP2 | FOXJ3 | GDF9 |
| HRASLS3 | BMP2K | NUSAP1 | ZNF22 | ICA1 | CLU | C19orf58 | SUFU | FTSJ1 | GEMIN7 |
| HSBP1 | BOMB | OAZ2 | AIG1 | IFT46 | CNOT8 | C19orf6 | SUPT4H1 | FXYD3 | GMPS |
| HSD17B2 | BOP1 | OAZIN | BEX1 | IGF1R | CNP | C1orf21 | SUPT7L | FXYD7 | GOT2 |
| HSPC121 | BRAF | OCLN | CCDC34 | IGFBP7 | COG6 | C1orf211 | TAF12 | GAB3 | GPRIN2 |
| HSPC132 | BRIX | OIP5 | CEP70 | IL12RB2 | COX4I1 | C1orf213 | TAL1 | GABRP | HIST1H2BC |
| HSPC163 | BRRN1 | ORC2L | CLGN | IL18 | COX6C | C1orf83 | TARBP2 | GAD2 | HMGB1 |
| HSPE1 | BST2 | ORC3L | EPDR1 | IL1B | COX7A2 | C20orf194 | TBC1D10B | GALNTL4 | HSPA2 |
| HTR2A | BUB1 | OSBPL9 | GPR125 | IMPACT | CRIPAK | C20orf42 | TBCC | GBP2 | HSPH1 |
| ICMT | BUB1B | PAK1IP1 | IFT81 | ING4 | CSTB | C20orf52 | TBK1 | GC | ITGB3BP |
| IGFBPL1 | BUB3 | PANK2 | KDM5B | ISOC1 | CTSO | C20orf96 | TBL1X | GFER | LRIF1 |
| IGJ | BXDC1 | PASK | LRRC16 | ISYNA1 | CTSZ | C22orf32 | TBP | GJA8 | LSM1 |
| IGSF3 | BZW1 | PAWR | MBOAT2 | ITGAV | CUL3 | C3orf10 | TCEAL8 | GJB1 | MNAT1 |
| IMP-1 | C10orf18 | PAXIP1L | ACY1 | JAKMIP2 | DCTD | C3orf19 | TCERG1 | GK5 | MRPS12 |
| IMP-2 | C10orf89 | PBEF1 | AHI1 | JRKL | DDR1 | C5orf4 | TCF20 | GLRB | MSH3 |
| IMPA2 | C11orf23 | PCNT1 | ALDH18A1 | JUP | DDX24 | C6orf117 | TFB2M | GLTSCR1 | MUTYH |
| INPP5F | C11orf73 | PDCD2 | ANGPT1 | KCNJ16 | DDX3X | C6orf166 | TFPT | GPR119 | MYBBP1A |
| INTU | C11orf82 | PDE6G | ATP2C1 | KCTD3 | DEGS1 | C8orf70 | THAP1 | GPR172B | NACC1 |
| IRX2 | C12orf11 | PDE9A | B3GALNT1 | KDM1A | DENND4C | C9orf19 | THAP8 | GSPT1 | NFKBIB |
| ISX | C13orf7 | PDK1 | BAALC | KIAA0087 | DERL1 | C9orf5 | TIAL1 | GTF2H3 | NOC2L |
| ITM2C | C14orf104 | PFDN4 | BBX | KIAA0485 | DLL1 | C9orf82 | TIAM1 | GUSB | NOP58 |
| KALRN | C14orf106 | PGM1 | BIVM | KIAA0753 | DNM1 | CA2 | TLCD1 | GYS1 | NPPB |
| KCNQ2 | C14orf115 | PHIP | CHCHD3 | KIAA1107 | DNM3 | CA4 | TLE2 | HADHA | NUP153 |
| KDELC1 | C14orf94 | PIG8 | CMTM4 | KIAA1305 | DUSP7 | CACNA1A | TLE3 | HCFC1 | NUP50 |
| KDM4A | C15orf15 | PIGW | CUTA | KIAA1462 | DYNLL1 | CACNA2D | TMED10 | HELZ | PAK3 |
| KIAA0522 | C1QBP | PIK3CB | CXXC5 | KIAA1467 | EBF1 | CALM2 | TMEM103 | HEMK1 | PCYT1B |
| KIAA0888 | C1QDC1 | PITX2 | DPY19L3 | KLHL23 | ECHDC1 | CALR | TMEM108 | HES6 | PELP1 |
| KIAA1166 | C1orf121 | PKD2 | DVL3 | KLHL3 | EDF1 | CAND1 | TMEM109 | HIST1H3B | PFDN6 |
| KIAA1576 | C1orf163 | PKP4 | EFCAB2 | KLHL9 | EEF1D | CARS2 | TMEM123 | HIST1H4G | PLSCR1 |
| KIAA1727 | C1orf38 | PLA2G3 | EFHA2 | LAMC1 | EHD4 | CASP9 | TMEM149 | HIVEP3 | PMF1 |
| KIF26A | C20orf160 | PLCB3 | EHBP1 | LAMP2 | EPB41L3 | CAV1 | TMEM160 | HMCN2 | POLG2 |
| KIF3C | C20orf6 | PLCL2 | ELN | LDLR | EPS15 | CCDC104 | TMEM16H | HMOX2 | POLQ |
| KLK8 | C20orf72 | PLK4 | EPM2AIP1 | LDLRAD4 | EPS8L2 | CCDC12 | TMEM170 | HNRNPD | POLR1C |
| KNOP1 | C21orf45 | PLP1 | FAM98A | LGALS9 | F11R | CCDC45 | TMEM43 | HS3ST3B1 | POLR1D |
| KPNA2 | C2orf29 | PLS1 | GOLGB1 | LHFP | FAM91A2 | CCDC93 | TMEM55B | HSPB7 | POP1 |
| KPNA6 | C2orf31 | PNMT | GOPC | LIMA1 | FAP | CCDC94 | TMEM59 | IBSP | POP5 |
| KRAS | C2orf56 | PNN | GPAM | LIMCH1 | FAT3 | CCT7 | TMEM60 | ICT1 | PPAN |
| KRT19 | C6orf115 | PODXL | GSTA4 | LMAN2L | FAU | CDC42EP4 | TMEM63A | IGFBP6 | PPID |
| KRT8 | C6orf139 | POLA | GSTM1 | LOC339229 | FGFR1OP2 | CDC45L | TMEM66 | IL1F10 | PTPN6 |
| KRTCAP3 | C6orf66 | POLD1 | GTF2H2 | LOC441528 | FN1 | CDS2 | TMEM77 | IMPG2 | RAD54L |
| LAGE3 | C9orf77 | POLE2 | LDOC1L | LOH11CR2A | GABBR1 | CEPT1 | TMEM87A | INCA1 | RAD9A |
| LAPTM4B | CA11 | POLR1B | LGALS3BP | LOX | GAS5 | CFL1 | TNFRSF12A | INO80E | RCHY1 |
| LARS | CABLES1 | POLR2D | LOC81691 | LRBA | GDI2 | CHCHD5 | TOMM40L | ISL1 | RECQL4 |
| LARS2 | CACNA2D3 | POU2F1 | LRP6 | LRP12 | GLTSCR2 | CHD2 | TRAF7 | ITPKA | RMND5B |
| LBH | CADPS2 | PPAP2A | MEIS1 | LRRC1 | GLUD1 | CITED2 | TRIP10 | ITSN2 | RND1 |
| LDB3 | CARHSP1 | PPAP2C | MNS1 | LRRC41 | GNG2 | CLIC1 | TRIP4 | JARID1C | RNMTL1 |
| LDHD | CBX3 | PPM1B | MUT | LUZP1 | GOLGA8B | CLN3 | TRPC4AP | JMJD2B | RPA1 |
| LEFTY2 | CBX5 | PPP1CC | NAP1L5 | LXN | GPRASP1 | CNN2 | TSC22D2 | KCTD18 | SIGLEC12 |
| LEPREL2 | CCAR1 | PPP1R16B | NDN | LYRM1 | HEY1 | CNN3 | TSSC1 | KEAP1 | SLC19A1 |
| LIMD2 | CCDC5 | PPP2R2B | NEK1 | LYRM4 | HINT3 | CNTNAP3 | TTC31 | KIAA0195 | SMN1 |
| LIN28B | CCKBR | PRIM1 | NENF | LZTFL1 | IDI1 | COG7 | TTF1 | KIAA1853 | SMNDC1 |
| LINC01139 | CCNA2 | PRKAA2 | NRIP1 | MAB21L2 | IL4R | COL4A6 | TUBG1 | KIF19 | SNRPC |
| LITAF | CCNB1 | PRKCB1 | OBSL1 | MAGED2 | IMPAD1 | COL7A1 | TXNDC5 | KIR3DL1 | SNURF |
| LMNB1 | CCRN4L | PRKCI | PAPSS1 | MAGEF1 | INHBC | COMMD3 | TYW3 | KIRREL2 | SP1 |
| LMNB2 | CCT5 | PRKCQ | PDE1A | MAGI2 | ITGB1 | COMMD7 | UBAP2 | KLC3 | SRSF2 |
| LOC389906 | CCT8 | PRKDC | PHGDH | MANEA | JAG1 | COPE | UBC | KLK5 | STXBP3 |
| LOC400451 | CD2AP | PRODH | PHYH | MANSC1 | JAK1 | COTL1 | UBE2D3 | KLRG1 | SUPT3H |
| LOXL2 | CDA | PRR6 | PIGF | MAP4K3 | KARS | CRYZ | UBE2S | KLRG2 | TDP2 |
| LPCAT1 | CDC2 | PSIP1 | PLEKHA5 | MBOAT7 | KCNQ1OT1 | CS | UBE2T | KPNA5 | TMSB4Y |
| LPHN2 | CDC20 | PSMA3 | PPIL4 | MCFD2 | KIAA0543 | CSNK1E | UBE2W | KREMEN1 | TOE1 |
| LRRN1 | CDC7 | PTPN2 | PRDM16 | MCM3AP-AS1 | KIAA1718 | CSTF1 | UBP1 | KRT39 | TRIM28 |
| LSM12 | CDH6 | PTPRG | PTPN11 | MCTP1 | KIFAP3 | CSTF3 | UBQLN4 | KRTAP22-1 | TUBA3C |
| LSM7 | CDT1 | PUS1 | RAB7B | MDH2 | KITLG | CTGF | UCRC | LCE1E | UNC119 |
| LUM | CDYL | PVRL3 | RHOBTB3 | ME3 | KLF12 | CUGBP2 | UFD1L | LIF | VAMP8 |
| LYPLA1 | CEB1 | PWP2H | SEH1L | MECOM | KLHL28 | CXorf26 | UIMC1 | LMO1 | WDR33 |
| LYPLA2 | CEBPZ | PYCR2 | SEPP1 | MED20 | KPNB1 | CYB5B | UNQ501 | LOC150759 | WDYHV1 |
| M6PR | CECR6 | Pfs2 | SERPING1 | MERTK | KRT14 | CYLD | URM1 | LOC151658 | WRAP73 |
| MACC1 | CENPA | RABGAP1L | SLC2A10 | METTL21B | KRT16 | CYP2R1 | USP10 | LOC154092 | WRN |
| MAGED4 | CENPE | RAD51 | SMAD5 | MFAP1 | KRT17 | CYR61 | USP16 | LOC220906 | ZNF165 |
| MALSU1 | CENPF | RAD54B | SPAG9 | MGMT | KRT6B | DCAKD | USP3 | LOC283440 | ZNF593 |
| MAP3K5 | CENPH | RAM2 | SV2A | MGST2 | LAMB3 | DCLRE1C | USP7 | LOC283951 |  |

**Supplementary Table 3.** The datasets of stem cell and normal adult tissue samples.

| Dataset | Type | Stem cells | Normal adult tisues |
| --- | --- | --- | --- |
| PCBC | RNA-seq | 81 | - |
| GSE90749 | RNA-seq | 98 | 20 |
| GSE30652 | Microarray | 159 | 32 |
| GTEx | RNA-seq | - | 1770 |

**Supplementary Table 4.** Experimentally validated stemness signature genes (SSGs).

| **SSGs** | **Article** | **Journal** | **Years** |
| --- | --- | --- | --- |
| TCF3 | Tcf3 governs stem cell features and represses cell fate determination in skin | Cell | 2006 |
| LIN28A | Induced Pluripotent Stem Cell Lines Derived from Human Somatic Cells | Science | 2007 |
| POU5F1 | Induced Pluripotent Stem Cell Lines Derived from Human Somatic Cells | Science | 2007 |
| SOX2 | Induced Pluripotent Stem Cell Lines Derived from Human Somatic Cells | Science | 2007 |
| NANOG | Induced Pluripotent Stem Cell Lines Derived from Human Somatic Cells | Science | 2007 |
| UTF1 | A UTF1-based selection system for stable homogeneously pluripotent human embryonic stem cell cultures | Nucleic Acids Research | 2007 |
| KLF4 | Generation of germline-competent induced pluripotent stem cells | Nature | 2007 |
| NFKB1 | A polymorphism in NFKB1 is associated with improved effect of interferon-{alpha} maintenance treatment of patients with multiple myeloma after high-dose treatment with stem cell support | Haematologica | 2009 |
| GDF3 | Testicular mixed germ cell tumors: a morphological and immunohistochemical study using stem cell markers, OCT3/4, SOX2 and GDF3, with emphasis on morphologically difficult-to-classify areas | Modern Pathology | 2009 |
| USP9X | USP9X enhances the polarity and self-renewal of embryonic stem cell-derived neural progenitors | Molecular Biology of the Cell | 2009 |
| JUNB | JunB protects against myeloid malignancies by limiting hematopoietic stem cell proliferation and differentiation without affecting self-renewal | Cancer Cell | 2009 |
| SKP2 | The role of Skp2 in hematopoietic stem cell quiescence, pool size, and self-renewal | Blood | 2011 |
| PRDM16 | Prdm16 is a physiologic regulator of hematopoietic stem cells | Blood | 2011 |
| USP44 | RNF20 and USP44 regulate stem cell differentiation by modulating H2B monoubiquitylation | Molecular Cell | 2012 |
| MEIS1 | Meis1 preserves hematopoietic stem cells in mice by limiting oxidative stress | Blood | 2012 |
| ZNF217 | The transcription factor ZNF217 is a prognostic biomarker and therapeutic target during breast cancer progression | Cancer Discovery | 2012 |
| CBX5 | Network biology of tumor stem-like cells identified a regulatory role of CBX5 in lung cancer | Scientific Reports | 2012 |
| ZFP42 | Expression of endogenous retroviruses is negatively regulated by the pluripotency marker Rex1/Zfp42 | Nucleic Acids Research | 2012 |
| GAL | Galanin promotes neuronal differentiation from neural progenitor cells in vitro and contributes to the generation of new olfactory neurons in the adult mouse brain | Experimental Neurology | 2014 |
| SIRT1 | Role of SIRT1 and AMPK in mesenchymal stem cells differentiation | Ageing Research Reviews | 2014 |
| NTS | Neurotensin signaling regulates stem-like traits of glioblastoma stem cells through activation of IL-8/CXCR1/STAT3 pathway | Cellular Signalling | 2014 |
| SEMA3C | Sema3C promotes the survival and tumorigenicity of glioma stem cells through Rac1 activation | Cell Reports | 2014 |
| NFE2L3 | Regulation and function of the NFE2 transcription factor in hematopoietic and non-hematopoietic cells | Cellular and Molecular Life Sciences | 2015 |
| LMO2 | The LIM-only transcription factor LMO2 determines tumorigenic and angiogenic traits in glioma stem cells | Cell Death Differentiation | 2015 |
| SOX4 | Sox4 Expression Confers Bladder Cancer Stem Cell Properties and Predicts for Poor Patient Outcome | International Journal of Biological Sciences | 2015 |
| NEFM | Identification of Global DNA Methylation Signatures in Glioblastoma-Derived Cancer Stem Cells | Journal of Genetics and Genomics | 2015 |
| FST | Human bone marrow mesenchymal stem cell-derived hepatocytes express tissue inhibitor of metalloproteinases 4 and follistatin | Liver International | 2015 |
| MGP | Matrix Gla protein regulates differentiation of endothelial cells derived from mouse embryonic stem cells | Angiogenesis | 2016 |
| NMU | Neuromedin U alters bioenergetics and expands the cancer stem cell phenotype in HER2-positive breast cancer | International Journal of Cancer | 2017 |
| ARID5B | ARID5B as a critical downstream target of the TAL1 complex that activates the oncogenic transcriptional program and promotes T-cell leukemogenesis | Genes & Development | 2017 |
| NODAL | Optimizing bone morphogenic protein 4-mediated human embryonic stem cell differentiation into trophoblast-like cells using fibroblast growth factor 2 and transforming growth factor-β/activin/nodal signalling inhibition | Reproductive BioMedicine Online | 2017 |
| TBX3 | Tbx3-dependent amplifying stem cell progeny drives interfollicular epidermal expansion during pregnancy and regeneration | Nature Communications | 2017 |
| ZMYM3 | Gene knockout of Zmym3 in mice arrests spermatogenesis at meiotic metaphase with defects in spindle assembly checkpoint | Cell Death Discovery | 2017 |
| SALL4 | SALL4 as a transcriptional and epigenetic regulator in normal and leukemic hematopoiesis | Biomarker Research | 2018 |
| TCF7L1 | Tcf7l1 directly regulates cardiomyocyte differentiation in embryonic stem cells | Stem Cell Research & Therapy | 2018 |
| MSI1 | Pyrin Inflammasome Regulates Tight Junction Integrity to Restrict Colitis and Tumorigenesis | Gastroenterology | 2018 |
| ZIC3 | ZIC3 Controls the Transition from Naive to Primed Pluripotency | Cell Reports | 2019 |
| PRDM14 | Off to a Bad Start: Cancer Initiation by Pluripotency Regulator PRDM14 | Trends In Genetics | 2019 |
| E2F3 | Chromatin remodeler HELLS maintains glioma stem cells through E2F3 and MYC | JCI Insight | 2019 |
| POU2F1 | Oct1/Pou2f1 is selectively required for colon regeneration and regulates colon malignancy | PLoS Genetics | 2019 |
| AHCY | Chromatin capture links the metabolic enzyme AHCY to stem cell proliferation | Science Advances | 2019 |
| INHBE | Human iPSC-derived MSCs (iMSCs) from aged individuals acquire a rejuvenation signature | Stem Cell Research & Therapy | 2019 |
| SALL1 | Generation of pluripotent stem cell-derived mouse kidneys in Sall1-targeted anephric rats | Nature Communications | 2019 |
| HLTF | A germline HLTF mutation in familial MDS induces DNA damage accumulation through impaired PCNA polyubiquitination | Leukemia | 2019 |
| ALDH1A1 | ALDH1A1 maintains the cancer stem-like cells properties of esophageal squamous cell carcinoma by activating the AKT signal pathway and interacting with β-catenin | Biomedicine & Pharmacotherapy | 2020 |
| TP53 | Recruiting TP53 to target chronic myeloid leukemia stem cells | Haematologica | 2020 |
| LEFTY1 | LEFTY1 Is a Dual-SMAD Inhibitor that Promotes Mammary Progenitor Growth and Tumorigenesis | Cell Stem Cell | 2020 |
| IGFBP7 | IGFBP7 activates retinoid acid-induced responses in acute myeloid leukemia stem and progenitor cells | Blood Advances | 2020 |
| CDH1 | Cdh1 functions as an oncogene by inducing self-renewal of lung cancer stem-like cells via oncogenic pathways | International Journal of Biological Sciences | 2020 |
| PFN2 | MicroRNA-dependent inhibition of PFN2 orchestrates ERK activation and pluripotent state transitions by regulating endocytosis | PNAS | 2020 |
| TCP1 | TCP1 regulates Wnt7b/β-catenin pathway through P53 to influence the proliferation and migration of hepatocellular carcinoma cells | Signal Transduction and Targeted Therapy | 2020 |
| MYCN | The MYCN oncogene and differentiation in neuroblastoma | Seminars in Cancer Biology | 2021 |
| HLF | HLF expression defines the human hematopoietic stem cell state | Blood | 2021 |
| SMARCA5 | Smarca5-mediated epigenetic programming facilitates fetal HSPC development in vertebrates | Blood | 2021 |
| BATF | BATF regulates progenitor to cytolytic effector CD8+ T cell transition during chronic viral infection | Nature Immunology | 2021 |
| CAPRIN1 | RNA degradation eliminates developmental transcripts during murine embryonic stem cell differentiation via CAPRIN1-XRN2 | Developmental Cell | 2022 |
| FOXJ2 | Germline FOXJ2 overexpression causes male infertility via aberrant autophagy activation by LAMP2A upregulation | Cell Death Discovery | 2022 |
| FGF2 | Role of TGFβ1 and WNT6 in FGF2 and BMP4-driven endothelial differentiation of murine embryonic stem cells | Angiogenesis | 2022 |
| SMARCA4 | Epigenetic Rewiring Underlies SMARCA4-Dependent Maintenance of Progenitor State in Pediatric H3K27M Diffuse Midline Glioma | Cancer Discovery | 2022 |
| EZH2 | Role of EZH2 in bone marrow mesenchymal stem cells and immune-cancer interactions | Critical Reviews in Oncology Hematology | 2022 |
| THY1 | Thy1 marks a distinct population of slow-cycling stem cells in the mouse epidermis | Nature Communications | 2022 |
| SNAI2 | Transcription factor SNAI2 exerts pro-tumorigenic effects on glioma stem cells via PHLPP2-mediated Akt pathway | Cell Death Discovery | 2022 |
| PXDN | The Value of the Stemness Index in Ovarian Cancer Prognosis | Genes | 2022 |
| PRRX1 | Prrx1 marks stem cells for bone, white adipose tissue and dermis in adult mice | Nature Genetics | 2022 |
| DNMT3B | DNMT3B supports meso-endoderm differentiation from mouse embryonic stem cells | Nature Communications | 2023 |
| HNRNPA1 | Cooperative regulation of Zhx1 and hnRNPA1 drives the cardiac progenitor-specific transcriptional activation during cardiomyocyte differentiation | Cell Death Discovery | 2023 |
| SMARCD3 | Smarcd3 is an epigenetic modulator of the metabolic landscape in pancreatic ductal adenocarcinoma | Nature Communications | 2023 |
| TUBB3 | Autologous olfactory mucosa mesenchymal stem cells treatment improves the neural network in chronic refractory epilepsy | Stem Cell Research & Therapy | 2023 |
| EPAS1 | The hypoxia-inducible factor EPAS1 is required for spermatogonial stem cell function in regenerative conditions | iScience | 2023 |
| TRIM28 | Trim28 citrullination maintains mouse embryonic stem cell pluripotency via regulating Nanog and Klf4 transcription | Science China-Life Sciences | 2023 |
| EXOSC7 | ZNF692 organizes a hub specialized in 40S ribosomal subunit maturation enhancing translation in rapidly proliferating cells | Cell Reports | 2023 |
| SPI1 | SPI1-mediated MIR222HG transcription promotes proneural-to-mesenchymal transition of glioma stem cells and immunosuppressive polarization of macrophages | Theranostics | 2023 |
| SCML2 | Discordant interactions between YAP1 and polycomb group protein SCML2 determine cell fate | iScience | 2023 |
| FOXO3 | FoxO3 Modulates Circadian Rhythms in Neural Stem Cells | International Journal of Molecular Sciences | 2023 |
| DPPA4 | DPPA4 increases aggressiveness of pituitary neuroendocrine tumors by enhancing cell stemness | Neuro-oncology | 2024 |
| SMARCC1 | A novel SMARCC1 BAFopathy implicates neural progenitor epigenetic dysregulation in human hydrocephalus | Brain | 2024 |
| EXOSC5 | EXOSC5 maintains cancer stem cell activity in endometrial cancer by regulating the NTN4/integrin β1 signalling axis | International Journal of Biological Sciences | 2024 |
| IGF2BP3 | WTAP/IGF2BP3 mediated m6A modification of the EGR1/PTEN axis regulates the malignant phenotypes of endometrial cancer stem cells | Journal of Experimental & Clinical Cancer Research | 2024 |
| GJA1 | KLF2 controls proliferation and apoptosis of human spermatogonial stem cells via targeting GJA1 | iScience | 2024 |
| CPSF3 | RBBP6 maintains glioblastoma stem cells through CPSF3-dependent alternative polyadenylation | Cell Discovery | 2024 |
| DCN | The fibro-adipogenic progenitor APOD+DCN+LUM+ cell population in aggressive carcinomas | Cancer and Metastasis Reviews | 2024 |
| DHX9 | DHX9 maintains epithelial homeostasis by restraining R-loop-mediated genomic instability in intestinal stem cells | Nature Communications | 2024 |
| EGLN3 | Identification of key genes of diabetic cardiomyopathy in hiPSCs-CMs based on bioinformatics analysis | Molecular and Cellular Biochemistry | 2024 |
| FKBP3 | FKBP3 aggravates the malignant phenotype of diffuse large B‐cell lymphoma by PARK7‐mediated activation of Wnt/β‐catenin signalling | Journal of Cellular and Molecular Medicine | 2024 |

**Supplementary Table 5.** Detailed list of the 18 immune-related pathways and their corresponding immune-related genes.

| **Antigen_Processing_and_Presentation** | AZGP1 | B2M | CALR | CANX | CD1A | CD1B | CD1C | CD1D | CD1E | CD4 |
| --- | --- | --- | --- | --- | --- | --- | --- | --- | --- | --- |
|  | CD74 | CD8A | CD8B | CREB1 | CTSB | CTSE | CTSL | CTSS | FCER1G | FCGRT |
|  | HFE | HLA-A | HLA-B | HLA-C | HLA-DMA | HLA-DMB | HLA-DOA | HLA-DOB | HLA-DPA1 | HLA-DPB1 |
|  | HLA-DQA1 | HLA-DQA2 | HLA-DQB1 | HLA-DRA | HLA-DRB1 | HLA-DRB3 | HLA-DRB4 | HLA-DRB5 | HLA-E | HLA-F |
|  | HLA-G | HLA-H | HSPA1A | HSPA1B | HSPA1L | HSPA2 | HSPA4 | HSPA5 | MR1 | PDIA3 |
| **Antimicrobials** | ABCC4 | ACKR2 | ACKR4 | ACO1 | ACTA1 | ACTG1 | ADAR | ADIPOQ | AEN | AGER |
|  | AHNAK | ALB | ANXA6 | APOBEC3A | APOBEC3C | APOBEC3F | APOBEC3H | APOH | APOM | AQP9 |
|  | ARG2 | ARRB1 | B2M | BACH2 | BCL3 | BECN1 | BIRC5 | BPHL | BPIFA2 | BST2 |
|  | CACYBP | CCL1 | CCL11 | CCL13 | CCL14 | CCL15 | CCL15-CCL14 | CCL16 | CCL17 | CCL18 |
|  | CCL19 | CCL2 | CCL20 | CCL21 | CCL22 | CCL23 | CCL24 | CCL25 | CCL26 | CCL27 |
|  | CCL28 | CCL3 | CCL3L1 | CCL3L3 | CCL4 | CCL4L1 | CCL4L2 | CCL5 | CCL7 | CCL8 |
|  | CCR1 | CCR10 | CCR3 | CCR4 | CCR5 | CCR6 | CCR7 | CCR8 | CD14 | CD4 |
|  | CD40 | CD40LG | CD81 | CD86 | CD8A | CDH1 | CHIT1 | CLDN4 | CREB1 | CRP |
|  | CSK | CSRP1 | CST4 | CTSG | CXCL1 | CXCL10 | CXCL11 | CXCL12 | CXCL2 | CXCR1 |
|  | CXCR4 | CXCR6 | CYBB | CYLD | DAXX | DCK | DDX17 | DEFB103B | DEFB4A | DES |
|  | DHX58 | DLL4 | DMBT1 | DUOX2 | ECD | EED | EIF2AK2 | ELAVL1 | ELN | EPPIN |
|  | F2R | F2RL1 | FASLG | FCN2 | FGA | FGF2 | FGR | FURIN | GBP2 | GDF15 |
|  | GFAP | GNAI1 | GNLY | GRK2 | GRN | HCK | HDAC1 | HFE | HGF | HLA-B |
|  | HMGB1 | HMOX1 | HRG | HSPA6 | HTR1A | IDO1 | IFIH1 | IFN1@ | IFNA1 | IFNAR2 |
|  | IFNL2 | IFNLR1 | IKBKE | IL10 | IL12B | IL13 | IL15 | IL17A | IL18 | IL1A |
|  | IL1B | IL2 | IL22 | IL27 | IL4 | IL6 | IL7R | ILK | IREB2 | IRF1 |
|  | IRF5 | IRF7 | IRF9 | ISG15 | ISG20 | ISG20L2 | ITGAV | JAK1 | JAK2 | JUN |
|  | JUND | KCNH2 | KLKB1 | KLRK1 | KNG1 | LALBA | LANCL1 | LEP | LIMS1 | LMBR1 |
|  | LRP1 | LTA | LTB4R | LTBP1 | LYZ | MAP2K1 | MAP2K2 | MAPK1 | MAPK14 | MAPK3 |
|  | MAPK8 | MAPT | MARCO | MASP1 | MASP2 | MIF | MMP9 | MPO | MSR1 | MUC4 |
|  | MX2 | NDRG1 | NEDD4 | NEO1 | NEWENTRY | NFKBIZ | NLRX1 | NOD1 | NOS1 | NOS2 |
|  | NOX3 | NOX5 | OAS1 | OLR1 | ORM1 | PCSK1 | PCSK2 | PDCD1 | PDF | PDGFRA |
|  | PDGFRB | PDYN | PGC | PIK3CG | PLA2G2A | PLAAT4 | PLSCR1 | PML | PPARG | PPIA |
|  | PPP4C | PRDX1 | PRDX2 | PROC | PRTN3 | PTGDR | PTGS2 | PTK2 | PTK2B | PTX3 |
|  | RELA | RN7SL1 | RNASE2 | RNASE3 | RNASE7 | RNASEL | ROBO3 | RSAD2 | SEMG1 | SEMG2 |
|  | SEPTIN7 | SERPINA3 | SKIV2L | SLC11A1 | SLC29A3 | SOCS1 | SOCS3 | SOD1 | SP1 | SPINK5 |
|  | SRC | STAB2 | STAT1 | STAT3 | STING1 | SYTL1 | TAFA1 | TAFA2 | TAFA3 | TAFA4 |
|  | TAFA5 | TANK | TBK1 | TCF7L2 | TFR2 | TFRC | TK2 | TKFC | TLR1 | TLR3 |
|  | TLR7 | TLR8 | TMPRSS6 | TNF | TNFAIP3 | TNFRSF10A | TNFRSF10B | TNFSF10 | TNFSF11 | TNFSF4 |
|  | TPM2 | TPT1 | TRAF3 | TRIM22 | TRIM27 | TRIM5 | TXK | TYK2 | UNC93B1 | VCAM1 |
|  | VDR | VEGFA | VIM | VTN | WNT5A | XCL1 | XCL2 | ZYX |  |  |
| **BCRSignalingPathway** | BLNK | BTK | CD79A | CD79B | CHP1 | CHP2 | HRAS | IGH | IGHD3-16 | IGHD3-22 |
|  | IGHD3-3 | IGHD3-9 | IGHD4-11 | IGHD4-17 | IGHD4-23 | IGHD4-4 | IGHD5-12 | IGHD5-18 | IGHD5-24 | IGHD5-5 |
|  | IGHD6-13 | IGHD6-19 | IGHD6-25 | IGHD6-6 | IGHD7-27 | IGHE | IGHG1 | IGHG2 | IGHG3 | IGHG4 |
|  | IGHJ1 | IGHJ2 | IGHJ3 | IGHJ4 | IGHJ5 | IGHJ6 | IGHM | IGHV1-18 | IGHV1-2 | IGHV1-24 |
|  | IGHV1-3 | IGHV1-38-4 | IGHV1-45 | IGHV1-46 | IGHV1-58 | IGHV1-69 | IGHV1-69-2 | IGHV1-8 | IGHV2-26 | IGHV2-5 |
|  | IGHV2-70 | IGHV3-11 | IGHV3-13 | IGHV3-15 | IGHV3-16 | IGHV3-20 | IGHV3-21 | IGHV3-23 | IGHV3-30 | IGHV3-30-3 |
|  | IGHV3-30-5 | IGHV3-33 | IGHV3-35 | IGHV3-38 | IGHV3-38-3 | IGHV3-43 | IGHV3-48 | IGHV3-49 | IGHV3-53 | IGHV3-64 |
|  | IGHV3-66 | IGHV3-69-1 | IGHV3-7 | IGHV3-72 | IGHV3-73 | IGHV3-74 | IGHV3-9 | IGHV4-28 | IGHV4-30-1 | IGHV4-30-2 |
|  | IGHV4-30-4 | IGHV4-31 | IGHV4-34 | IGHV4-38-2 | IGHV4-39 | IGHV4-4 | IGHV4-59 | IGHV4-61 | IGHV5-10-1 | IGHV5-51 |
|  | IGHV6-1 | IGHV7-4-1 | IGHV7-81 | IGK | IGKC | IGKDEL | IGKJ | IGKJ1 | IGKJ2 | IGKJ3 |
|  | IGKJ4 | IGKJ5 | IGKV@ | IGKV1-12 | IGKV1-13 | IGKV1-16 | IGKV1-17 | IGKV1-27 | IGKV1-33 | IGKV1-37 |
|  | IGKV1-39 | IGKV1-5 | IGKV1-6 | IGKV1-8 | IGKV1-9 | IGKV1D-12 | IGKV1D-13 | IGKV1D-16 | IGKV1D-17 | IGKV1D-33 |
|  | IGKV1D-37 | IGKV1D-39 | IGKV1D-42 | IGKV1D-43 | IGKV1D-8 | IGKV2-24 | IGKV2-28 | IGKV2-30 | IGKV2-40 | IGKV2D-24 |
|  | IGKV2D-28 | IGKV2D-29 | IGKV2D-30 | IGKV2D-40 | IGKV3-11 | IGKV3-15 | IGKV3-20 | IGKV3-7 | IGKV3D-11 | IGKV3D-15 |
|  | IGKV3D-20 | IGKV3D-7 | IGKV4-1 | IGKV5-2 | IGKV6-21 | IGKV6D-21 | IGKV6D-41 | IGL | IGLC1 | IGLC2 |
|  | IGLC3 | IGLC6 | IGLC7 | IGLJ | IGLJ1 | IGLJ2 | IGLJ3 | IGLJ4 | IGLJ5 | IGLJ6 |
|  | IGLJ7 | IGLV@ | IGLV1-36 | IGLV1-40 | IGLV1-44 | IGLV1-47 | IGLV1-50 | IGLV1-51 | IGLV10-54 | IGLV11-55 |
|  | IGLV2-11 | IGLV2-14 | IGLV2-18 | IGLV2-23 | IGLV2-33 | IGLV2-8 | IGLV3-1 | IGLV3-10 | IGLV3-12 | IGLV3-16 |
|  | IGLV3-19 | IGLV3-21 | IGLV3-22 | IGLV3-25 | IGLV3-27 | IGLV3-32 | IGLV3-9 | IGLV4-3 | IGLV4-60 | IGLV4-69 |
|  | IGLV5-37 | IGLV5-39 | IGLV5-45 | IGLV5-48 | IGLV5-52 | IGLV6-57 | IGLV7-43 | IGLV7-46 | IGLV8-61 | IGLV9-49 |
|  | KRAS | LYN | NFAT5 | NFATC1 | NFATC2 | NFATC3 | NFATC4 | PPP3CA | PPP3CB | PPP3CC |
|  | PPP3R1 | PPP3R2 | RAC1 | RAC2 | RAC3 | SYK | VAV1 | VAV2 | VAV3 |  |
| **Chemokines** | C3 | C5 | CAMP | CCL1 | CCL11 | CCL13 | CCL14 | CCL15 | CCL15-CCL14 | CCL16 |
|  | CCL17 | CCL18 | CCL19 | CCL2 | CCL20 | CCL21 | CCL22 | CCL23 | CCL24 | CCL25 |
|  | CCL26 | CCL27 | CCL28 | CCL3 | CCL3L1 | CCL3L3 | CCL3P1 | CCL4 | CCL4L1 | CCL4L2 |
|  | CCL5 | CCL7 | CCL8 | CCN1 | CKLF | CMA1 | CTSG | CX3CL1 | CXCL1 | CXCL10 |
|  | CXCL11 | CXCL12 | CXCL13 | CXCL14 | CXCL16 | CXCL17 | CXCL2 | CXCL3 | CXCL5 | CXCL6 |
|  | CXCL8 | CXCL9 | DEFA1 | DEFA3 | DEFA5 | DEFB1 | DEFB103B | DEFB104A | DEFB4A | EDN1 |
|  | EDN2 | EDN3 | FGF10 | FGF2 | HTN3 | LECT2 | PF4 | PF4V1 | PLAU | PPBP |
|  | PPBPP1 | PROK2 | RNASE2 | SAA1 | SAA2 | SBDS | SEMA3A | SEMA3B | SEMA3C | SEMA3D |
|  | SEMA3E | SEMA3F | SEMA3G | SEMA4A | SEMA4B | SEMA4C | SEMA4D | SEMA4F | SEMA4G | SEMA5A |
|  | SEMA5B | SEMA6A | SEMA6B | SEMA6C | SEMA6D | SEMA7A | SLIT1 | SLIT2 | TNC | TYMP |
|  | XCL1 | XCL2 |  |  |  |  |  |  |  |  |
| **Chemokine_Receptors** | ACKR1 | ACKR2 | ACKR3 | ACKR4 | C5AR1 | C5AR2 | CCR1 | CCR10 | CCR3 | CCR4 |
|  | CCR5 | CCR6 | CCR7 | CCR8 | CCR9 | CCRL2 | CMKLR1 | CX3CR1 | CXCR1 | CXCR2 |
|  | CXCR3 | CXCR4 | CXCR5 | CXCR6 | CYSLTR1 | CYSLTR2 | EDNRA | EDNRB | FPR1 | FPR2 |
|  | GPR17 | GPR32 | GPR33 | LTB4R | LTB4R2 | PLAUR | PLXNA1 | PLXNA2 | PLXNA3 | PLXNA4 |
|  | PLXNB1 | PLXNB2 | PLXNB3 | PLXNC1 | PLXND1 | PTAFR | PTGDR2 | ROBO1 | ROBO2 | ROBO3 |
|  | RXFP3 | XCR1 |  |  |  |  |  |  |  |  |
| **Cytokines** | ADA2 | ADIPOQ | ADM | ADM2 | AGRP | AGT | AIMP1 | AMBN | AMELX | AMH |
|  | ANGPTL5 | ANGPTL7 | APLN | AREG | ARTN | AVP | AZU1 | BDNF | BMP1 | BMP10 |
|  | BMP15 | BMP2 | BMP3 | BMP4 | BMP5 | BMP6 | BMP7 | BMP8A | BMP8B | BTC |
|  | C3 | C5 | CALCA | CALCB | CAMP | CAT | CCK | CCL1 | CCL11 | CCL13 |
|  | CCL14 | CCL15 | CCL15-CCL14 | CCL16 | CCL17 | CCL18 | CCL19 | CCL2 | CCL20 | CCL21 |
|  | CCL22 | CCL23 | CCL24 | CCL25 | CCL26 | CCL27 | CCL28 | CCL3 | CCL3L1 | CCL3L3 |
|  | CCL3P1 | CCL4 | CCL4L1 | CCL4L2 | CCL5 | CCL7 | CCL8 | CCN1 | CCN2 | CCN3 |
|  | CD320 | CD40LG | CD70 | CDNF | CER1 | CGA | CGB1 | CGB2 | CGB3 | CGB5 |
|  | CGB7 | CGB8 | CHGA | CHGB | CKLF | CLCF1 | CLEC11A | CMA1 | CMTM1 | CMTM2 |
|  | CMTM3 | CMTM4 | CMTM5 | CMTM6 | CMTM7 | CMTM8 | CNTF | CORT | CRH | CSF1 |
|  | CSF2 | CSF3 | CSH1 | CSH2 | CSHL1 | CSPG5 | CTF1 | CTSG | CX3CL1 | CXCL1 |
|  | CXCL10 | CXCL11 | CXCL12 | CXCL13 | CXCL14 | CXCL16 | CXCL17 | CXCL2 | CXCL3 | CXCL5 |
|  | CXCL6 | CXCL8 | CXCL9 | DEFA1 | DEFA3 | DEFA5 | DEFB1 | DEFB103B | DEFB104A | DEFB4A |
|  | DKK1 | EBI3 | EDN1 | EDN2 | EDN3 | EGF | ENDOU | EPGN | EPO | EREG |
|  | ESM1 | FAM3B | FAM3C | FAM3D | FASLG | FGF1 | FGF10 | FGF11 | FGF12 | FGF13 |
|  | FGF14 | FGF16 | FGF17 | FGF18 | FGF19 | FGF2 | FGF20 | FGF21 | FGF22 | FGF23 |
|  | FGF3 | FGF4 | FGF5 | FGF6 | FGF7 | FGF7P3 | FGF7P6 | FGF8 | FGF9 | FIGNL2 |
|  | FLT3LG | FSHB | GAL | GALP | GAST | GCG | GDF1 | GDF10 | GDF11 | GDF15 |
|  | GDF2 | GDF3 | GDF5 | GDF6 | GDF7 | GDF9 | GDNF | GH1 | GH2 | GHRH |
|  | GHRL | GIP | GKN1 | GMFB | GMFG | GNRH1 | GNRH2 | GPHA2 | GPHB5 | GPI |
|  | GREM1 | GREM2 | GRN | GRP | GUCA2A | HAMP | HBEGF | HDGF | HDGFL3 | HGF |
|  | HTN3 | IAPP | IFNA1 | IFNA10 | IFNA13 | IFNA14 | IFNA16 | IFNA17 | IFNA2 | IFNA21 |
|  | IFNA4 | IFNA5 | IFNA6 | IFNA7 | IFNA8 | IFNB1 | IFNE | IFNG | IFNK | IFNL1 |
|  | IFNL2 | IFNL3 | IFNW1 | IGF1 | IGF2 | IL10 | IL11 | IL12A | IL12B | IL13 |
|  | IL15 | IL16 | IL17A | IL17B | IL17C | IL17D | IL17F | IL18 | IL19 | IL1A |
|  | IL1B | IL1F10 | IL1RN | IL2 | IL20 | IL21 | IL22 | IL23A | IL24 | IL25 |
|  | IL26 | IL27 | IL3 | IL31 | IL32 | IL33 | IL34 | IL36A | IL36B | IL36G |
|  | IL36RN | IL37 | IL4 | IL5 | IL6 | IL6ST | IL7 | IL9 | INHA | INHBA |
|  | INHBB | INHBC | INHBE | INS | INS-IGF2 | INSL3 | INSL4 | INSL5 | INSL6 | JAG1 |
|  | JAG2 | KITLG | KL | LACRT | LECT2 | LEFTY1 | LEFTY2 | LEP | LHB | LIF |
|  | LRSAM1 | LTA | LTB | LTBP1 | LTBP2 | LTBP3 | LTBP4 | MANF | MDK | MIA |
|  | MIF | MLN | MSTN | MYDGF | NAMPT | NDP | NENF | NGF | NMB | NODAL |
|  | NPFF | NPPA | NPPB | NPPC | NPY | NRG1 | NRG2 | NRG3 | NRG4 | NRTN |
|  | NTF3 | NTF4 | NTS | NUDT6 | OGN | OSGIN1 | OSM | OSTN | OXT | PDGFA |
|  | PDGFB | PDGFC | PDGFD | PDGFRA | PDGFRB | PDGFRL | PDYN | PENK | PF4 | PF4V1 |
|  | PGF | PLAU | PMCH | PNOC | POMC | PPBP | PPBPP1 | PPBPP2 | PPY | PRL |
|  | PRLH | PROK1 | PROK2 | PSPN | PTH | PTH2 | PTHLH | PTN | PYY | QRFP |
|  | RABEP1 | RABEP2 | REG1A | RETN | RETNLB | RLN1 | RLN2 | RLN3 | RNASE2 | S100A6 |
|  | SAA1 | SAA2 | SBDS | SCG2 | SCGB3A1 | SCT | SECTM1 | SEMA3A | SEMA3B | SEMA3C |
|  | SEMA3D | SEMA3E | SEMA3F | SEMA3G | SEMA4A | SEMA4B | SEMA4C | SEMA4D | SEMA4F | SEMA4G |
|  | SEMA5A | SEMA5B | SEMA6A | SEMA6B | SEMA6C | SEMA6D | SEMA7A | SLIT1 | SLIT2 | SLURP1 |
|  | SPP1 | SST | STC1 | STC2 | TAC1 | TDGF1 | TDGF1P3 | TG | TGFA | TGFB1 |
|  | TGFB2 | TGFB3 | THPO | TNC | TNF | TNFRSF11B | TNFSF10 | TNFSF11 | TNFSF12 | TNFSF13 |
|  | TNFSF13B | TNFSF14 | TNFSF15 | TNFSF18 | TNFSF4 | TNFSF8 | TNFSF9 | TOR2A | TRH | TSHB |
|  | TSLP | TXLNA | TYMP | UCN | UCN2 | UCN3 | UTS2 | UTS2B | VEGFA | VEGFB |
|  | VEGFC | VEGFD | VGF | VIP | XCL1 | XCL2 |  |  |  |  |
| **Cytokine_Receptors** | ACKR1 | ACKR2 | ACKR3 | ACKR4 | ACVR1B | ACVR1C | ACVR2A | ACVR2B | ACVRL1 | ADCYAP1R1 |
|  | ADIPOR1 | ADIPOR2 | ADRB1 | ADRB2 | AGTR1 | AGTR2 | AMHR2 | ANGPT1 | ANGPT4 | ANGPTL1 |
|  | ANGPTL2 | ANGPTL3 | ANGPTL4 | ANGPTL6 | APLNR | AR | AVPR1A | AVPR1B | AVPR2 | BMPR1A |
|  | BMPR1B | BMPR2 | BRD8 | C3AR1 | C5AR1 | C5AR2 | CALCR | CALCRL | CCR1 | CCR10 |
|  | CCR3 | CCR4 | CCR5 | CCR6 | CCR7 | CCR8 | CCR9 | CCRL2 | CD40 | CMKLR1 |
|  | CNTFR | CRHR1 | CRHR2 | CRIM1 | CRLF1 | CRLF2 | CRLF3 | CSF1R | CSF2RA | CSF2RB |
|  | CSF3R | CX3CR1 | CXCR1 | CXCR2 | CXCR3 | CXCR4 | CXCR5 | CXCR6 | CYSLTR1 | CYSLTR2 |
|  | EDNRA | EDNRB | EGFR | ENG | EPOR | ESR1 | ESR2 | ESRRA | ESRRB | ESRRG |
|  | FGFR1 | FGFR2 | FGFR3 | FGFR4 | FGFRL1 | FLT1 | FLT3 | FLT4 | FPR1 | FPR2 |
|  | FSHR | GALR2 | GALR3 | GCGR | GHR | GHRHR | GHSR | GIPR | GLP1R | GLP2R |
|  | GNRHR | GPER1 | GPR17 | GPR32 | GPR33 | HNF4A | HNF4G | HTR3A | HTR3B | HTR3C |
|  | HTR3D | HTR3E | IFNAR1 | IFNAR2 | IFNGR1 | IFNGR2 | IFNLR1 | IGF1R | IGF2R | IL10RA |
|  | IL10RB | IL11RA | IL12RB1 | IL12RB2 | IL13RA1 | IL13RA2 | IL15RA | IL17RA | IL17RB | IL17RC |
|  | IL17RD | IL17RE | IL18R1 | IL18RAP | IL1R1 | IL1R2 | IL1RAP | IL1RL1 | IL1RL2 | IL20RA |
|  | IL20RB | IL21R | IL22RA1 | IL22RA2 | IL23R | IL27RA | IL2RA | IL2RB | IL2RG | IL31RA |
|  | IL3RA | IL4R | IL5RA | IL6R | IL7R | IL9R | INSR | KDR | LEPR | LGR4 |
|  | LGR5 | LGR6 | LHCGR | LIFR | LTB4R | LTB4R2 | LTBR | MC1R | MC2R | MC3R |
|  | MC4R | MCHR1 | MCHR2 | MET | MLNR | MPL | MTNR1A | MTNR1B | NGFR | NMBR |
|  | NPR1 | NPR3 | NR0B1 | NR0B2 | NR1D1 | NR1D2 | NR1H2 | NR1H3 | NR1H4 | NR1I2 |
|  | NR1I3 | NR2C1 | NR2C2 | NR2E1 | NR2E3 | NR2F1 | NR2F2 | NR2F6 | NR3C1 | NR3C2 |
|  | NR4A1 | NR4A2 | NR4A3 | NR5A1 | NR5A2 | NR6A1 | NRP1 | NRP2 | OGFR | OPRD1 |
|  | OPRK1 | OPRL1 | OPRM1 | OSMR | OXTR | PGR | PGRMC2 | PLAUR | PLXNA1 | PLXNA2 |
|  | PLXNA3 | PLXNA4 | PLXNB1 | PLXNB2 | PLXNB3 | PLXNC1 | PLXND1 | PPARA | PPARD | PPARG |
|  | PRLHR | PRLR | PTAFR | PTGDR | PTGDR2 | PTGDS | PTGER1 | PTGER2 | PTGER3 | PTGER4 |
|  | PTGFR | PTH1R | PTH2R | RARA | RARB | RARG | ROBO1 | ROBO2 | ROBO3 | RORA |
|  | RORB | RORC | RXFP1 | RXFP2 | RXFP3 | RXRA | RXRB | RXRG | S1PR1 | S1PR2 |
|  | SCTR | SDC1 | SDC2 | SDC3 | SDC4 | SORT1 | SSTR1 | SSTR2 | SSTR5 | ST2 |
|  | TACR1 | TEK | TGFBR1 | TGFBR2 | TGFBR3 | THRA | THRB | TIE1 | TNFRSF10A | TNFRSF10B |
|  | TNFRSF10C | TNFRSF10D | TNFRSF11A | TNFRSF12A | TNFRSF13B | TNFRSF13C | TNFRSF14 | TNFRSF17 | TNFRSF18 | TNFRSF19 |
|  | TNFRSF1A | TNFRSF1B | TNFRSF21 | TNFRSF25 | TNFRSF4 | TNFRSF6B | TNFRSF8 | TNFRSF9 | TRHR | TSHR |
|  | TUBB3 | VDR | VIPR1 | VIPR2 | XCR1 |  |  |  |  |  |
| **Interferons** | IFNA10 | IFNA13 | IFNA14 | IFNA16 | IFNA17 | IFNA2 | IFNA21 | IFNA4 | IFNA5 | IFNA6 |
|  | IFNA7 | IFNA8 | IFNB1 | IFNE | IFNG | IFNK | IFNW1 |  |  |  |
| **Interferon_Receptor** | IFNAR2 | IFNGR1 | IFNGR2 |  |  |  |  |  |  |  |
| **Interleukins** | CXCL8 | IFNL1 | IFNL2 | IFNL3 | IL11 | IL12A | IL12B | IL13 | IL15 | IL16 |
|  | IL17A | IL17B | IL17C | IL17D | IL17F | IL18 | IL19 | IL1A | IL1B | IL1F10 |
|  | IL1RN | IL2 | IL20 | IL21 | IL22 | IL23A | IL24 | IL25 | IL26 | IL27 |
|  | IL3 | IL31 | IL32 | IL33 | IL34 | IL36A | IL36B | IL36G | IL36RN | IL37 |
|  | IL4 | IL5 | IL6 | IL6ST | IL7 | IL9 | TXLNA |  |  |  |
| **Interleukins_Receptor** | CXCR1 | CXCR2 | IFNLR1 | IL10RA | IL10RB | IL11RA | IL12RB1 | IL12RB2 | IL13RA1 | IL13RA2 |
|  | IL15RA | IL17RA | IL17RB | IL17RC | IL17RD | IL17RE | IL18R1 | IL18RAP | IL1R1 | IL1R2 |
|  | IL1RAP | IL1RL1 | IL1RL2 | IL20RA | IL20RB | IL21R | IL22RA1 | IL22RA2 | IL23R | IL27RA |
|  | IL2RA | IL2RB | IL2RG | IL31RA | IL3RA | IL4R | IL5RA | IL6R | IL7R | IL9R |
|  | ST2 |  |  |  |  |  |  |  |  |  |
| **NaturalKiller_Cell_Cytotoxicity** | CD247 | CSF2 | FCER1G | FCGR3A | FCGR3B | FYN | GRB2 | HLA-A | HLA-B | HLA-C |
|  | HLA-E | HLA-G | HRAS | ICAM1 | ICAM2 | IFNG | ITGAL | ITGB2 | KIR2DL1 | KIR2DL2 |
|  | KIR2DL3 | KIR2DL4 | KIR2DL5A | KIR2DS1 | KIR2DS3 | KIR2DS4 | KIR2DS5 | KIR3DL1 | KIR3DL2 | KLRC1 |
|  | KLRC2 | KLRC3 | KLRD1 | LAT | LCK | LCP2 | MAP2K1 | MAP2K2 | MAPK1 | MAPK3 |
|  | NCR1 | NCR2 | NCR3 | PAK1 | PIK3CA | PIK3CB | PIK3CD | PIK3CG | PIK3R1 | PIK3R2 |
|  | PIK3R3 | PIK3R5 | PLCG1 | PLCG2 | PTK2B | PTPN11 | PTPN6 | RAC1 | RAC2 | RAC3 |
|  | SH3BP2 | SHC1 | SHC2 | SHC3 | SHC4 | SOS1 | SOS2 | SYK | TNF | TYROBP |
|  | VAV1 | VAV2 | VAV3 | ZAP70 |  |  |  |  |  |  |
| **TCRsignalingPathway** | AKT1 | AKT2 | AKT3 | BCL10 | CARD11 | CBL | CBLB | CBLC | CD28 | CD40LG |
|  | CDK4 | CHUK | CSF2 | CTLA4 | FOS | ICOS | IFNG | IKBKB | IKBKG | IL10 |
|  | IL2 | IL4 | IL5 | JUN | KRAS | MALT1 | MAP3K14 | MAP3K8 | NFKB1 | NFKBIA |
|  | NFKBIB | NFKBIE | NRAS | PDCD1 | PDK1 | PIK3CA | PIK3CB | PIK3CD | PIK3CG | PIK3R1 |
|  | PIK3R2 | PIK3R3 | PIK3R5 | PLCG1 | PRKCQ | PTPN6 | RASGRP1 | RELA | TNF | TRAC |
|  | TRAJ1 | TRAJ10 | TRAJ11 | TRAJ12 | TRAJ13 | TRAJ14 | TRAJ15 | TRAJ16 | TRAJ17 | TRAJ18 |
|  | TRAJ19 | TRAJ2 | TRAJ20 | TRAJ21 | TRAJ22 | TRAJ23 | TRAJ24 | TRAJ25 | TRAJ26 | TRAJ27 |
|  | TRAJ28 | TRAJ29 | TRAJ3 | TRAJ30 | TRAJ31 | TRAJ32 | TRAJ33 | TRAJ34 | TRAJ35 | TRAJ36 |
|  | TRAJ37 | TRAJ38 | TRAJ39 | TRAJ4 | TRAJ40 | TRAJ41 | TRAJ42 | TRAJ43 | TRAJ44 | TRAJ45 |
|  | TRAJ46 | TRAJ47 | TRAJ48 | TRAJ49 | TRAJ5 | TRAJ50 | TRAJ52 | TRAJ53 | TRAJ54 | TRAJ56 |
|  | TRAJ57 | TRAJ58 | TRAJ59 | TRAJ6 | TRAJ61 | TRAJ7 | TRAJ8 | TRAJ9 | TRAV1-1 | TRAV1-2 |
|  | TRAV10 | TRAV12-1 | TRAV12-2 | TRAV12-3 | TRAV13-1 | TRAV13-2 | TRAV14DV4 | TRAV16 | TRAV17 | TRAV18 |
|  | TRAV19 | TRAV2 | TRAV20 | TRAV21 | TRAV22 | TRAV23DV6 | TRAV24 | TRAV25 | TRAV26-1 | TRAV26-2 |
|  | TRAV27 | TRAV29DV5 | TRAV3 | TRAV30 | TRAV34 | TRAV35 | TRAV36DV7 | TRAV38-1 | TRAV38-2DV8 | TRAV39 |
|  | TRAV4 | TRAV40 | TRAV41 | TRAV5 | TRAV6 | TRAV7 | TRAV8-1 | TRAV8-2 | TRAV8-3 | TRAV8-4 |
|  | TRAV8-6 | TRAV8-7 | TRAV9-1 | TRAV9-2 | TRBC1 | TRBC2 | TRBD1 | TRBD2 | TRBJ1-1 | TRBJ1-2 |
|  | TRBJ1-3 | TRBJ1-4 | TRBJ1-5 | TRBJ1-6 | TRBJ2-1 | TRBJ2-2 | TRBJ2-3 | TRBJ2-4 | TRBJ2-5 | TRBJ2-6 |
|  | TRBJ2-7 | TRBV10-1 | TRBV10-2 | TRBV10-3 | TRBV11-1 | TRBV11-2 | TRBV11-3 | TRBV12-3 | TRBV12-4 | TRBV12-5 |
|  | TRBV13 | TRBV14 | TRBV15 | TRBV16 | TRBV17 | TRBV18 | TRBV19 | TRBV2 | TRBV20-1 | TRBV24-1 |
|  | TRBV25-1 | TRBV27 | TRBV28 | TRBV29-1 | TRBV3-1 | TRBV30 | TRBV4-1 | TRBV4-2 | TRBV4-3 | TRBV5-1 |
|  | TRBV5-4 | TRBV5-5 | TRBV5-6 | TRBV5-7 | TRBV5-8 | TRBV6-1 | TRBV6-2 | TRBV6-3 | TRBV6-4 | TRBV6-5 |
|  | TRBV6-6 | TRBV6-7 | TRBV6-8 | TRBV6-9 | TRBV7-2 | TRBV7-3 | TRBV7-4 | TRBV7-6 | TRBV7-7 | TRBV7-8 |
|  | TRBV7-9 | TRBV9 | TRDC | TRDD1 | TRDD2 | TRDD3 | TRDJ1 | TRDJ2 | TRDJ3 | TRDJ4 |
|  | TRDV1 | TRDV2 | TRDV3 | TRGC1 | TRGC2 | TRGJ1 | TRGJ2 | TRGJP | TRGJP1 | TRGJP2 |
|  | TRGV2 | TRGV3 | TRGV4 | TRGV5 | TRGV8 | TRGV9 |  |  |  |  |
| **TGFb_Family_Member** | BMP1 | BMP10 | BMP15 | BMP2 | BMP3 | BMP4 | BMP5 | BMP6 | BMP7 | BMP8A |
|  | BMP8B | GDF1 | GDF10 | GDF11 | GDF15 | GDF2 | GDF3 | GDF5 | GDF6 | GDF7 |
|  | GDF9 | GDNF | INHA | INHBA | INHBB | INHBC | INHBE | LEFTY1 | LEFTY2 | NODAL |
|  | TGFB1 | TGFB2 | TGFB3 |  |  |  |  |  |  |  |
| **TGFb_Family_Member_Receptor** | ACVR1B | ACVR1C | ACVR2A | ACVR2B | ACVRL1 | AMHR2 | BMPR1A | BMPR1B | BMPR2 | TGFBR1 |
|  | TGFBR2 | TGFBR3 |  |  |  |  |  |  |  |  |
| **TNF_Family_Members** | TNFRSF11B | TNFSF10 | TNFSF11 | TNFSF12 | TNFSF13 | TNFSF13B | TNFSF14 | TNFSF15 | TNFSF18 | TNFSF4 |
|  | TNFSF8 | TNFSF9 |  |  |  |  |  |  |  |  |
| **TNF_Family_Members_Receptors** | TNFRSF10B | TNFRSF10C | TNFRSF10D | TNFRSF11A | TNFRSF12A | TNFRSF13B | TNFRSF13C | TNFRSF14 | TNFRSF17 | TNFRSF18 |
|  | TNFRSF19 | TNFRSF1A | TNFRSF1B | TNFRSF21 | TNFRSF25 | TNFRSF4 | TNFRSF6B | TNFRSF8 | TNFRSF9 |  |
| **GO** | A1BG | A2M | ABCA13 | ABCC9 | ABCE1 | ABCF3 | ABI1 | ABL1 | ABL2 | ABR |
|  | ACAA1 | ACE | ACIN1 | ACLY | ACOD1 | ACTB | ACTN1 | ACTR10 | ACTR1A | ACTR1B |
|  | ACTR2 | ACTR3 | ADA | ADAM10 | ADAM15 | ADAM17 | ADAM8 | ADAM9 | ADAMDEC1 | ADAMTS13 |
|  | ADARB1 | ADCYAP1 | ADD1 | ADD2 | ADGRB1 | ADGRE1 | ADGRE2 | ADGRE3 | ADGRF5 | ADGRG3 |
|  | ADORA1 | ADORA2B | AGA | AGBL4 | AGBL5 | AGL | AGO1 | AGO3 | AGO4 | AGPAT2 |
|  | AGPAT5 | AHCY | AHR | AHSG | AHSP | AICDA | AIF1 | AIM2 | AIRE | AKAP17A |
|  | AKAP8 | AKIRIN2 | ALAD | ALAS2 | ALCAM | ALDH3B1 | ALDOA | ALDOC | ALOX15 | ALOX5 |
|  | AMBP | AMPD3 | ANGPT2 | ANKHD1 | ANKHD1-EIF4EBP3 | ANKRD17 | ANKRD54 | ANLN | ANO6 | ANPEP |
|  | ANXA1 | ANXA2 | ANXA3 | AOC1 | AP1B1 | AP1G1 | AP1M1 | AP1M2 | AP1S1 | AP1S2 |
|  | AP1S3 | AP2A1 | AP2A2 | AP2B1 | AP2M1 | AP2S1 | AP3B1 | AP3D1 | APAF1 | APBB1IP |
|  | APCS | APEH | APLF | APOA1 | APOA2 | APOA4 | APOB | APOBEC1 | APOBEC3B | APOBEC3D |
|  | APOBEC3G | APOD | APOL1 | APP | APRT | AQP3 | AQP4 | ARF1 | ARHGAP45 | ARHGAP9 |
|  | ARHGEF2 | ARHGEF5 | ARHGEF7 | ARID4A | ARID5A | ARIH2 | ARL8A | ARMC6 | ARMC8 | ARNT |
|  | ARPC1A | ARPC1B | ARPC2 | ARPC3 | ARPC4 | ARPC5 | ARRB2 | ARSA | ARSB | ASAH1 |
|  | ASH2L | ASXL1 | ATAD3B | ATAD5 | ATG5 | ATG7 | ATM | ATOX1 | ATP11A | ATP11B |
|  | ATP11C | ATP1B1 | ATP1B2 | ATP1B3 | ATP6AP1 | ATP6AP2 | ATP6V0A1 | ATP6V0A2 | ATP6V0C | ATP6V1D |
|  | ATP7A | ATP8A1 | ATP8B4 | ATXN1L | AZI2 | B4GALT1 | BAD | BAG6 | BAIAP2 | BAK1 |
|  | BANK1 | BAP1 | BARX1 | BATF | BATF2 | BATF3 | BAX | BCAP31 | BCAR1 | BCL11B |
|  | BCL2 | BCL2L11 | BCL6 | BCL6B | BCR | BDKRB1 | BGLAP | BIN2 | BIRC2 | BIRC3 |
|  | BLK | BMX | BNIP3 | BNIP3L | BPGM | BPI | BPIFA1 | BPIFB1 | BPIFB2 | BPIFB3 |
|  | BRCA2 | BRI3 | BRK1 | BSG | BST1 | BTN2A3P | BTNL10 | BTNL3 | BTNL8 | BTNL9 |
|  | BTRC | BVES | C12orf29 | C12orf4 | C1QA | C1QB | C1QBP | C1QC | C1R | C1RL |
|  | C1S | C2 | C4A | C4B | C4BPA | C4BPB | C6 | C6orf120 | C7 | C8A |
|  | C8B | C8G | C9 | CA2 | CAB39 | CACNA1C | CACNA1F | CACNB3 | CADM1 | CADM3 |
|  | CALCOCO2 | CALM1 | CALML5 | CAMK1D | CAMK2A | CAMK2B | CAMK2D | CAMK2G | CAMK4 | CAND1 |
|  | CAP1 | CAPN1 | CAPZA1 | CAPZA2 | CAPZA3 | CAPZB | CARD9 | CARTPT | CASP1 | CASP3 |
|  | CASP4 | CASP8 | CASP9 | CAV1 | CBFA2T3 | CBFB | CCDC88B | CCNB2 | CCND3 | CCT2 |
|  | CCT8 | CD101 | CD151 | CD164 | CD177 | CD180 | CD19 | CD2 | CD207 | CD209 |
|  | CD24 | CD248 | CD300A | CD300C | CD300E | CD300LB | CD300LD | CD300LF | CD300LG | CD33 |
|  | CD34 | CD36 | CD3D | CD3E | CD3G | CD46 | CD5 | CD53 | CD55 | CD58 |
|  | CD59 | CD5L | CD6 | CD63 | CD68 | CD7 | CD83 | CD8B2 | CD93 | CD99 |
|  | CDA | CDC37 | CDC42 | CDC42EP2 | CDC42EP4 | CDC73 | CDH17 | CDK13 | CDKN1A | CDKN2B |
|  | CEACAM1 | CEACAM20 | CEACAM3 | CEACAM5 | CEACAM6 | CEACAM8 | CEBPA | CEBPB | CEBPE | CEBPG |
|  | CENPE | CEP290 | CFB | CFD | CFH | CFHR1 | CFHR2 | CFHR4 | CFHR5 | CFI |
|  | CFP | CGAS | CH25H | CHD2 | CHD7 | CHI3L1 | CHIA | CHID1 | CHRNA4 | CHRNB2 |
|  | CHRNB4 | CHST4 | CIITA | CITED1 | CKAP4 | CLDN1 | CLDN18 | CLEC10A | CLEC12A | CLEC12B |
|  | CLEC1B | CLEC2A | CLEC2B | CLEC2D | CLEC4A | CLEC4C | CLEC4D | CLEC4E | CLEC4G | CLEC4M |
|  | CLEC5A | CLEC6A | CLEC7A | CLECL1 | CLNK | CLPTM1 | CLTA | CLTC | CLU | CNIH1 |
|  | CNN2 | CNOT7 | CNPY3 | CNR1 | CNR2 | COCH | COL17A1 | COL1A1 | COL1A2 | COL24A1 |
|  | COL2A1 | COL3A1 | COLEC10 | COLEC11 | COLEC12 | COMMD3 | COMMD9 | COPB1 | CORO1A | COTL1 |
|  | CPB2 | CPLX2 | CPN1 | CPN2 | CPNE3 | CPPED1 | CR1 | CR2 | CRACR2A | CRCP |
|  | CREB3 | CREBBP | CREG1 | CRIP1 | CRIP2 | CRISP3 | CRISPLD2 | CRK | CRKL | CRTAM |
|  | CRTC3 | CSNK2B | CST3 | CST7 | CST9 | CSTB | CTC1 | CTNNB1 | CTNNBIP1 | CTNNBL1 |
|  | CTPS1 | CTR9 | CTSA | CTSC | CTSD | CTSF | CTSH | CTSK | CTSV | CTSW |
|  | CTSZ | CUEDC2 | CUL1 | CUL4A | CXADR | CYB5R3 | CYBA | CYFIP1 | CYFIP2 | CYP11B1 |
|  | CYP19A1 | CYP26B1 | CYP27B1 | CYP7B1 | CYSTM1 | DAB2IP | DAPK1 | DAPK2 | DAPK3 | DBH |
|  | DBNL | DCAF1 | DCD | DCLRE1C | DCST1 | DCSTAMP | DCTN1 | DCTN2 | DCTN3 | DCTN4 |
|  | DCTN5 | DCTN6 | DDIT4 | DDOST | DDT | DDX3X | DDX41 | DDX58 | DDX60 | DEFA4 |
|  | DEFA6 | DEFB103A | DEFB105A | DEFB106A | DEFB107A | DEFB108A | DEFB108B | DEFB110 | DEFB112 | DEFB113 |
|  | DEFB114 | DEFB115 | DEFB116 | DEFB118 | DEFB119 | DEFB123 | DEFB124 | DEFB125 | DEFB126 | DEFB127 |
|  | DEFB128 | DEFB129 | DEFB131A | DEFB131B | DEFB132 | DEFB133 | DEFB134 | DEFB135 | DEGS1 | DENND1B |
|  | DERA | DGAT1 | DHPS | DHRS2 | DHTKD1 | DHX9 | DIAPH1 | DLG1 | DLG5 | DLL1 |
|  | DMTN | DNAJA3 | DNAJB9 | DNAJC13 | DNAJC3 | DNAJC5 | DNASE1 | DNASE1L1 | DNASE1L3 | DNASE2 |
|  | DNM2 | DOCK1 | DOCK10 | DOCK11 | DOCK2 | DOCK8 | DOK2 | DOK3 | DPP4 | DPP7 |
|  | DPP8 | DPY30 | DRD2 | DROSHA | DSC1 | DSG1 | DSN1 | DSP | DTX1 | DTX3L |
|  | DUSP10 | DUSP22 | DUSP3 | DYNC1H1 | DYNC1I1 | DYNC1I2 | DYNC1LI1 | DYNC1LI2 | DYNLL1 | DYNLL2 |
|  | DYNLT1 | DYRK3 | EBP | ECM1 | ECSIT | EDA | EEF1A1 | EEF2 | EFNA2 | EFNA4 |
|  | EFNB1 | EFNB2 | EFNB3 | EGR1 | EIF2AK1 | EIF2AK4 | EIF2B1 | EIF2B2 | EIF2B3 | EIF2B4 |
|  | EIF2B5 | EIF6 | ELANE | ELF1 | ELF2 | ELF4 | ELMO1 | ELMO2 | ELMOD2 | ELP1 |
|  | EML1 | EMP2 | ENPP1 | ENPP2 | ENPP3 | ENPP4 | EOMES | EP300 | EPB42 | EPCAM |
|  | EPHA2 | EPHB1 | EPHB3 | EPS8 | EPX | ERAP1 | ERAP2 | ERBB2 | ERBIN | ERCC1 |
|  | ERCC2 | ERMAP | ERP44 | ESAM | ESCO2 | ETS1 | ETV2 | ETV6 | EVI2B | EVL |
|  | EXO1 | EXOC1 | EXOSC3 | EXOSC4 | EXOSC5 | EXOSC6 | EXOSC9 | EZR | F11R | F12 |
|  | F2 | F7 | FABP5 | FADD | FAF2 | FAM111A | FAM20C | FAM210B | FAM3A | FANCA |
|  | FANCC | FANCD2 | FARP2 | FAS | FBN1 | FBXO7 | FBXO9 | FBXW11 | FBXW7 | FCAMR |
|  | FCAR | FCER1A | FCGR1A | FCGR2A | FCGR2B | FCGR2C | FCMR | FCN3 | FCRL1 | FCRL3 |
|  | FCRL4 | FCRLB | FES | FFAR2 | FFAR3 | FGB | FGG | FGL2 | FKBP1A | FKBP1B |
|  | FLCN | FLG2 | FLI1 | FLNB | FLOT1 | FLOT2 | FLVCR1 | FN1 | FNIP1 | FOLR3 |
|  | FOXC1 | FOXE1 | FOXF1 | FOXJ1 | FOXL1 | FOXN1 | FOXO3 | FOXP1 | FOXP3 | FPR3 |
|  | FRK | FRMPD3 | FST | FSTL3 | FTH1 | FTL | FUCA1 | FUCA2 | FUT10 | FUT7 |
|  | FYB1 | FYB2 | FZD5 | FZD7 | FZD8 | FZD9 | G6PD | GAA | GAB2 | GAB3 |
|  | GABPA | GALNS | GALNT2 | GAPDH | GAPT | GAS6 | GATA1 | GATA2 | GATA3 | GBF1 |
|  | GBP1 | GBP3 | GBP5 | GBP6 | GCA | GCH1 | GCNT1 | GCNT3 | GCSAM | GCSAML |
|  | GDI2 | GEM | GFI1 | GGH | GGT1 | GHDC | GLA | GLB1 | GLG1 | GLI2 |
|  | GLI3 | GLIPR1 | GLMN | GLO1 | GLRX5 | GLYCAM1 | GM2A | GNAS | GNL1 | GNS |
|  | GOLGA7 | GOLPH3 | GON4L | GP1BA | GP6 | GPAM | GPATCH3 | GPC1 | GPC3 | GPLD1 |
|  | GPNMB | GPR15 | GPR171 | GPR174 | GPR18 | GPR183 | GPR32P1 | GPR55 | GPR65 | GPR68 |
|  | GPR84 | GPRC5B | GPSM3 | GPX1 | GRAP | GRAP2 | GRB14 | GRB7 | GSDMD | GSTP1 |
|  | GTPBP1 | GUCY1A1 | GUSB | GYG1 | GYPA | GYPB | GYPC | GZMB | GZMH | GZMM |
|  | HAND2 | HAX1 | HBB | HBZ | HCAR2 | HCLS1 | HCST | HDAC4 | HDAC5 | HDAC9 |
|  | HEATR9 | HERC5 | HERC6 | HES1 | HEXB | HEXIM1 | HGSNAT | HHEX | HIF1A | HIPK1 |
|  | HIPK2 | HK1 | HK3 | HLA-DQB2 | HLX | HMGB2 | HMGB3 | HMGN2 | HMHB1 | HMOX2 |
|  | HMSD | HNF1A | HOXA3 | HOXA5 | HOXA7 | HOXA9 | HOXB3 | HOXB4 | HOXB6 | HOXB7 |
|  | HOXB8 | HP | HPRT1 | HPSE | HPX | HRH1 | HRH2 | HRNR | HSD3B7 | HSH2D |
|  | HSP90AA1 | HSP90AB1 | HSP90B1 | HSPA8 | HSPA9 | HSPD1 | HSPH1 | HTN1 | HTRA1 | HUWE1 |
|  | HVCN1 | HYAL2 | ICAM3 | ICAM4 | ICAM5 | ID2 | IDH1 | IFI16 | IFI27 | IFI30 |
|  | IFI35 | IFI44L | IFI6 | IFIT1 | IFIT1B | IFIT2 | IFIT3 | IFIT5 | IFITM1 | IFITM2 |
|  | IFITM3 | IFNL4 | IGBP1 | IGFBP2 | IGHA1 | IGHA2 | IGHD | IGHD1-1 | IGHV1-69D | IGHV1OR15-1 |
|  | IGHV1OR15-9 | IGHV1OR21-1 | IGHV2-70D | IGHV2OR16-5 | IGHV3-64D | IGHV3OR15-7 | IGHV3OR16-10 | IGHV3OR16-12 | IGHV3OR16-13 | IGHV3OR16-8 |
|  | IGHV3OR16-9 | IGHV4OR15-8 | IGKV1OR2-108 | IGKV2-29 | IGKV2D-26 | IGKV3OR2-268 | IGLL1 | IGLL5 | IGSF6 | IHH |
|  | IKZF1 | IKZF3 | IL18BP | ILF2 | ILF3 | IMPDH1 | IMPDH2 | INAVA | INPP5D | INPPL1 |
|  | IP6K2 | IPO7 | IQGAP1 | IQGAP2 | IRAK1 | IRAK1BP1 | IRAK2 | IRAK3 | IRAK4 | IRF2 |
|  | IRF3 | IRF4 | IRF6 | IRF8 | IRGM | IRS2 | IST1 | ITCH | ITFG2 | ITGA1 |
|  | ITGA2 | ITGA2B | ITGA3 | ITGA4 | ITGA5 | ITGA6 | ITGA9 | ITGAD | ITGAM | ITGAX |
|  | ITGB1 | ITGB3 | ITGB5 | ITGB7 | ITK | ITLN1 | ITM2A | ITPKB | JAGN1 | JAK3 |
|  | JAM2 | JAM3 | JAML | JARID2 | JCHAIN | JMJD6 | JUNB | JUP | KAAG1 | KAT2A |
|  | KAT2B | KAT6A | KAT8 | KCMF1 | KCNAB2 | KCNJ8 | KCNN4 | KDELR1 | KDM5D | KDM6B |
|  | KIF11 | KIF13B | KIF16B | KIF18A | KIF22 | KIF23 | KIF26A | KIF2A | KIF2B | KIF2C |
|  | KIF3A | KIF3B | KIF3C | KIF4A | KIF4B | KIF5A | KIF5B | KIFAP3 | KIRREL1 | KIRREL3 |
|  | KIT | KLC1 | KLC2 | KLF1 | KLF10 | KLF13 | KLF2 | KLF4 | KLF6 | KLHL6 |
|  | KLK3 | KLK5 | KLK7 | KLRB1 | KLRF1 | KLRF2 | KMT2A | KMT2B | KMT2C | KMT2D |
|  | KMT2E | KPNB1 | KRT1 | KRT16 | KRT6A | KRT75 | KYNU | L1CAM | L3MBTL1 | L3MBTL3 |
|  | LAMP1 | LAMP2 | LAMP3 | LAMTOR1 | LAMTOR2 | LAMTOR3 | LAT2 | LAX1 | LBP | LCN2 |
|  | LCP1 | LDB1 | LDLR | LEAP2 | LEF1 | LFNG | LGALS1 | LGMN | LIG4 | LILRA1 |
|  | LILRA4 | LIME1 | LIMK1 | LMO1 | LMO2 | LMO4 | LNPEP | LOXL3 | LPCAT1 | LPXN |
|  | LRCH1 | LRG1 | LRP5 | LRRC14 | LRRC17 | LRRC32 | LRRC7 | LRRC70 | LRRC8A | LRRK1 |
|  | LRRK2 | LSM14A | LST1 | LTA4H | LTF | LY6D | LY75 | LY86 | LY96 | LYAR |
|  | LYL1 | LYST | MAD1L1 | MADCAM1 | MAEA | MAFB | MAG | MAGT1 | MAN2B1 | MANBA |
|  | MAP2K4 | MAP2K6 | MAP2K7 | MAP3K1 | MAP3K5 | MAP3K7 | MAP4K2 | MAPK10 | MAPK9 | MAPKAPK2 |
|  | MAPKAPK3 | MATK | MATR3 | MAVS | MB | MBL2 | MBP | MCEMP1 | MCM3AP | MCOLN1 |
|  | MCOLN2 | MECOM | MED1 | MEF2C | MEFV | MEIS1 | MEIS2 | MELK | MEOX1 | METTL3 |
|  | METTL7A | MFAP5 | MFNG | MGAM | MGST1 | MIA3 | MICA | MICB | MID1 | MID2 |
|  | MILR1 | MITF | MIXL1 | MKNK2 | MLEC | MLF1 | MLH1 | MME | MMP1 | MMP12 |
|  | MMP14 | MMP2 | MMP21 | MMP25 | MMP28 | MMP8 | MNDA | MNX1 | MOG | MOSPD2 |
|  | MOV10 | MPIG6B | MPP1 | MPTX1 | MRC1 | MRGPRX2 | MS4A1 | MS4A2 | MS4A3 | MSH2 |
|  | MSH3 | MSH6 | MSN | MSRB1 | MST1 | MST1L | MST1R | MT-RNR2 | MT1G | MT2A |
|  | MTHFD1 | MTOR | MTUS1 | MUC1 | MUC12 | MUC13 | MUC15 | MUC16 | MUC17 | MUC19 |
|  | MUC2 | MUC20 | MUC21 | MUC3A | MUC5AC | MUC5B | MUC6 | MUC7 | MUL1 | MVP |
|  | MX1 | MYB | MYC | MYH2 | MYH9 | MYL9 | MYO10 | MYO18A | MYO1C | MYO1E |
|  | MYO1G | MZB1 | N4BP2L2 | NAIP | NAPRT | NBEAL2 | NBL1 | NBN | NCAM1 | NCBP3 |
|  | NCF1 | NCF2 | NCF4 | NCK1 | NCK2 | NCKAP1 | NCKAP1L | NCKIPSD | NCOA6 | NCSTN |
|  | NDFIP1 | NDUFC2 | NECTIN1 | NECTIN2 | NEU1 | NF1 | NFAM1 | NFASC | NFE2 | NFE2L1 |
|  | NFE2L2 | NFIL3 | NFKB2 | NFKBID | NFKBIL1 | NHEJ1 | NHLRC3 | NIT2 | NKAP | NKX2-3 |
|  | NKX2-5 | NKX3-2 | NLRC3 | NLRC4 | NLRC5 | NLRP1 | NLRP10 | NLRP11 | NLRP12 | NLRP13 |
|  | NLRP14 | NLRP2 | NLRP2B | NLRP3 | NLRP4 | NLRP5 | NLRP6 | NLRP7 | NLRP8 | NLRP9 |
|  | NME1 | NME2 | NMI | NOD2 | NONO | NOP53 | NOTCH1 | NOTCH2 | NOTCH4 | NPC2 |
|  | NPDC1 | NPHS1 | NPLOC4 | NPY5R | NRARP | NRROS | NSD2 | NT5C3A | NTRK1 | NUB1 |
|  | NUDCD1 | NUP85 | OAS2 | OAS3 | OASL | OCSTAMP | OLFM4 | ONECUT1 | OPTN | ORAI1 |
|  | ORM2 | ORMDL3 | OSBPL1A | OSCAR | OSTF1 | OSTM1 | OTUB1 | OTUD4 | OTUD7A | OTUD7B |
|  | OTULIN | P2RX1 | P2RX7 | P2RY12 | P4HTM | PA2G4 | PABPC4 | PADI2 | PADI4 | PAF1 |
|  | PAFAH1B1 | PAFAH1B2 | PAG1 | PAK2 | PAK3 | PARK7 | PARP1 | PARP14 | PARP9 | PATZ1 |
|  | PAWR | PAX1 | PAX5 | PAXIP1 | PBX1 | PCBP2 | PCID2 | PDAP1 | PDCD2 | PDE12 |
|  | PDE1B | PDE4B | PDE4D | PDE5A | PDPK1 | PDPN | PDXK | PDZD11 | PECAM1 | PELI1 |
|  | PELI2 | PELI3 | PFKL | PGAM1 | PGLYRP1 | PGLYRP2 | PGLYRP3 | PGLYRP4 | PGM1 | PGM2 |
|  | PGM3 | PGRMC1 | PHLPP1 | PHPT1 | PI3 | PI4K2A | PIANP | PIAS1 | PIBF1 | PICALM |
|  | PIGR | PIK3AP1 | PIK3C3 | PIK3R4 | PIK3R6 | PILRA | PILRB | PIP5K1C | PITX2 | PKHD1L1 |
|  | PKM | PKN1 | PKNOX1 | PKP1 | PLA2G10 | PLA2G1B | PLA2G2D | PLA2G2F | PLA2G3 | PLA2G6 |
|  | PLA2G7 | PLAC8 | PLCB1 | PLCL2 | PLD1 | PLD2 | PLD4 | PLEK | PLEKHA1 | PLEKHO2 |
|  | PLPP4 | PLVAP | PMAIP1 | PMS2 | PMS2P1 | PMS2P11 | PMS2P2 | PMS2P3 | PNMA1 | PNP |
|  | PODXL2 | POLL | POLM | POLQ | POLR3A | POLR3B | POLR3C | POLR3D | POLR3E | POLR3F |
|  | POLR3G | POLR3H | POLR3K | POU1F1 | POU2AF1 | POU2F2 | POU4F1 | POU4F2 | PPARGC1B | PPIE |
|  | PPIL2 | PPM1B | PPP1R14B | PPP2R3C | PPP6C | PQBP1 | PRAM1 | PRCP | PRDM1 | PRDX3 |
|  | PRDX4 | PRDX6 | PRELID1 | PREX1 | PRG2 | PRG3 | PRG4 | PRKACA | PRKACB | PRKACG |
|  | PRKAR1A | PRKCA | PRKCB | PRKCD | PRKCE | PRKCH | PRKCZ | PRKD1 | PRKD2 | PRKRA |
|  | PRKX | PRMT6 | PRNP | PROCR | PROS1 | PRR7 | PRRC2C | PRSS2 | PRSS3 | PSAP |
|  | PSEN1 | PSG1 | PSMA1 | PSMA2 | PSMA3 | PSMA4 | PSMA5 | PSMA6 | PSMA7 | PSMA8 |
|  | PSMB1 | PSMB10 | PSMB11 | PSMB2 | PSMB3 | PSMB4 | PSMB5 | PSMB6 | PSMB7 | PSMB8 |
|  | PSMB9 | PSMC1 | PSMC2 | PSMC3 | PSMC4 | PSMC5 | PSMC6 | PSMD1 | PSMD10 | PSMD11 |
|  | PSMD12 | PSMD13 | PSMD14 | PSMD2 | PSMD3 | PSMD4 | PSMD5 | PSMD6 | PSMD7 | PSMD8 |
|  | PSMD9 | PSME1 | PSME2 | PSME3 | PSME4 | PSMF1 | PSPC1 | PSTPIP1 | PTBP3 | PTGES2 |
|  | PTK6 | PTMS | PTPN1 | PTPN2 | PTPN22 | PTPRB | PTPRC | PTPRJ | PTPRN2 | PTPRO |
|  | PTPRS | PTPRZ1 | PUM1 | PUM2 | PURB | PVR | PXDN | PYCARD | PYDC1 | PYDC2 |
|  | PYGB | PYGO1 | QPCT | QSOX1 | RAB10 | RAB12 | RAB14 | RAB17 | RAB18 | RAB20 |
|  | RAB24 | RAB27A | RAB29 | RAB31 | RAB32 | RAB33A | RAB34 | RAB35 | RAB37 | RAB3A |
|  | RAB3B | RAB3C | RAB3D | RAB43 | RAB44 | RAB4A | RAB4B | RAB5B | RAB5C | RAB6A |
|  | RAB7A | RAB7B | RAB8B | RAB9B | RACGAP1 | RAET1E | RAET1G | RAET1L | RAF1 | RAG1 |
|  | RAG2 | RAP1A | RAP1B | RAP2B | RAP2C | RAPGEF1 | RARRES2 | RASAL3 | RASGRP4 | RASSF2 |
|  | RB1 | RBBP5 | RBCK1 | RBFOX2 | RBM14 | RBM15 | RBM47 | RBP4 | RBPJ | RC3H1 |
|  | RC3H2 | REG1B | REG3A | REG3G | RELB | RELT | REST | RET | RFTN1 | RFTN2 |
|  | RFX1 | RGCC | RGS1 | RHAG | RHBDD3 | RHEX | RHOA | RHOF | RHOG | RHOH |
|  | RIF1 | RILP | RIOK3 | RIPK1 | RIPK2 | RIPK3 | RIPOR2 | RNASE6 | RNASE8 | RNASET2 |
|  | RNF125 | RNF135 | RNF168 | RNF19B | RNF216 | RNF26 | RNF31 | RNF41 | RNF8 | ROCK1 |
|  | ROGDI | ROMO1 | ROR2 | RPL13A | RPL30 | RPL39 | RPS14 | RPS17 | RPS19 | RPS24 |
|  | RPS27A | RPS3 | RPS6 | RPS6KA3 | RPS6KA5 | RRAS | RRS1 | RTKN2 | RTP4 | RUBCN |
|  | RUNX1 | RUNX2 | RUNX3 | S100A1 | S100A11 | S100A12 | S100A13 | S100A14 | S100A7 | S100A8 |
|  | S100B | S100P | S1PR4 | SAMHD1 | SAMSN1 | SAR1B | SARM1 | SART1 | SART3 | SASH3 |
|  | SATB1 | SBNO2 | SBSPON | SCAMP1 | SCARB1 | SCGB1A1 | SCIN | SCRIB | SDCBP | SDHAF4 |
|  | SEC13 | SEC14L1 | SEC22B | SEC23A | SEC24A | SEC24B | SEC24C | SEC24D | SEC31A | SEC61A1 |
|  | SELENOK | SELENOS | SELL | SELP | SELPLG | SENP1 | SERINC3 | SERINC5 | SERPINA1 | SERPINB1 |
|  | SERPINB10 | SERPINB12 | SERPINB3 | SERPINB4 | SERPINB6 | SERPINB9 | SERPINC1 | SERPINE1 | SERPING1 | SETD1A |
|  | SETD2 | SFPQ | SFRP1 | SFRP2 | SFTPA1 | SFTPA2 | SFTPD | SFXN1 | SGPL1 | SH2B3 |
|  | SH2D1A | SH2D1B | SH2D6 | SH3GL2 | SH3PXD2A | SHH | SHMT2 | SHPK | SIAE | SIGLEC10 |
|  | SIGLEC14 | SIGLEC15 | SIGLEC16 | SIGLEC5 | SIGLEC7 | SIGLEC9 | SIN3A | SIPA1L3 | SIRPG | SIRT1 |
|  | SIRT2 | SIT1 | SIX1 | SIX4 | SKAP1 | SKAP2 | SKIL | SKP1 | SLA | SLA2 |
|  | SLAMF1 | SLC11A2 | SLC15A4 | SLC16A1 | SLC16A3 | SLC16A8 | SLC25A6 | SLC26A6 | SLC27A2 | SLC2A3 |
|  | SLC2A5 | SLC30A8 | SLC39A10 | SLC39A3 | SLC3A2 | SLC40A1 | SLC44A2 | SLC46A2 | SLC7A10 | SLC7A11 |
|  | SLC7A2 | SLC7A5 | SLC7A6 | SLC7A6OS | SLC7A7 | SLC7A8 | SLC7A9 | SLC8A3 | SLC8B1 | SLC9B2 |
|  | SLCO4C1 | SLFN11 | SLFN13 | SLPI | SMAD3 | SMAD6 | SMAD7 | SMAP1 | SMIM1 | SMPD3 |
|  | SMPDL3B | SNAP23 | SNAP25 | SNAP29 | SNCA | SNRK | SNX27 | SNX4 | SOCS5 | SOCS6 |
|  | SOX11 | SOX13 | SOX4 | SOX6 | SP100 | SP2 | SP3 | SP7 | SPACA3 | SPG21 |
|  | SPI1 | SPN | SPNS2 | SPON2 | SPPL2A | SPPL2B | SPPL3 | SPTA1 | SPTAN1 | SPTBN2 |
|  | SQSTM1 | SRF | SRMS | SRP14 | SRPK1 | SRPK2 | SSBP3 | SSC5D | ST6GAL1 | STAP1 |
|  | STAR | STAT2 | STAT5B | STAT6 | STBD1 | STK10 | STK11 | STK11IP | STK3 | STK4 |
|  | STOM | STOML2 | STON2 | STX4 | STX7 | STX8 | STXBP1 | STXBP2 | STXBP3 | STXBP4 |
|  | STYK1 | SUMO1 | SURF4 | SUSD2 | SUSD4 | SVIP | SWAP70 | SYNCRIP | SYNGR1 | TAB1 |
|  | TAB2 | TAB3 | TAL1 | TAP1 | TAP2 | TAPBP | TAPBPL | TARBP2 | TBC1D10C | TBKBP1 |
|  | TBX1 | TBX21 | TCF12 | TCF21 | TCF3 | TCF7 | TCIM | TCIRG1 | TCN1 | TCTA |
|  | TEC | TENM1 | TESC | TESPA1 | TET2 | TFE3 | TFEB | THBD | THBS1 | THBS4 |
|  | THEMIS | THEMIS2 | THOC1 | THOC5 | THY1 | TICAM1 | TICAM2 | TIMP2 | TINAG | TINAGL1 |
|  | TIPARP | TJP2 | TLR10 | TLR2 | TLR5 | TLR6 | TMBIM1 | TMBIM6 | TMC6 | TMEM102 |
|  | TMEM131 | TMEM131L | TMEM176A | TMEM176B | TMEM178A | TMEM179B | TMEM190 | TMEM30A | TMEM63A | TMEM91 |
|  | TMIGD2 | TMOD3 | TNFAIP1 | TNFAIP6 | TNFAIP8L2 | TNFSF12-TNFSF13 | TNIP1 | TNIP2 | TNIP3 | TNK1 |
|  | TNK2 | TNRC6A | TNRC6B | TNRC6C | TOB2 | TOLLIP | TOM1 | TOP2A | TP53 | TP53BP1 |
|  | TP73 | TPD52 | TPO | TRAF2 | TRAF3IP1 | TRAF3IP2 | TRAF6 | TRAFD1 | TRAPPC1 | TRAT1 |
|  | TREM1 | TREML1 | TREML2 | TREML4 | TRIB1 | TRIL | TRIM10 | TRIM11 | TRIM13 | TRIM14 |
|  | TRIM15 | TRIM21 | TRIM23 | TRIM25 | TRIM26 | TRIM28 | TRIM29 | TRIM31 | TRIM32 | TRIM34 |
|  | TRIM35 | TRIM4 | TRIM44 | TRIM56 | TRIM58 | TRIM59 | TRIM6 | TRIM62 | TRIM68 | TRIM8 |
|  | TRPM2 | TRPM4 | TRPV1 | TRPV4 | TSC1 | TSC22D3 | TSC22D4 | TSPAN14 | TSPAN2 | TSPAN32 |
|  | TSPAN6 | TTBK1 | TTC7A | TTR | TUBB | TUBB4B | TWSG1 | TXNDC5 | TYR | UBA52 |
|  | UBASH3A | UBASH3B | UBB | UBC | UBD | UBE2D1 | UBE2D2 | UBE2D3 | UBE2K | UBE2N |
|  | UBE2V1 | UBQLN1 | UBR4 | UFD1 | ULBP1 | ULBP2 | ULBP3 | UMOD | UNC13D | UNG |
|  | USP14 | USP17L2 | USP18 | VAMP2 | VAMP3 | VAMP7 | VAMP8 | VAPA | VAT1 | VCL |
|  | VCP | VNN1 | VPREB1 | VPREB3 | VPS26B | VPS33A | VPS33B | VSIG4 | VSTM1 | WAS |
|  | WASF2 | WASL | WDFY1 | WDR1 | WDR5 | WDR61 | WDR7 | WIPF1 | WIPF2 | WIPF3 |
|  | WNT1 | WNT10B | WNT2B | WNT3A | WNT4 | WRNIP1 | XAF1 | XBP1 | XIAP | XRCC5 |
|  | XRCC6 | YAP1 | YES1 | YPEL5 | YTHDF2 | ZBP1 | ZBTB1 | ZBTB16 | ZBTB24 | ZBTB46 |
|  | ZBTB7B | ZC3H12A | ZC3H8 | ZC3HAV1 | ZEB1 | ZFAT | ZFP36 | ZFP36L1 | ZFP36L2 | ZFPM1 |
|  | ZMIZ1 | ZMYND11 | ZNF16 | ZNF160 | ZNF175 | ZNF3 | ZNF335 | ZNF385A | ZNF580 | ZNF675 |
|  | ZNF683 | ZNF784 | ZP3 | ZP4 |  |  |  |  |  |  |

**Supplementary Table 6.** Melanoma dataset from anti-PD-1 therapy cohorts.

| **Cohort** | **Cancer type** | **Therapy** | **Sample** | **Responder number** | **Non-responder number** |
| --- | --- | --- | --- | --- | --- |
| GSE78220 | Melanoma | anti-PD-1 | 28 | 15 | 13 |
| GSE91061 | Melanoma | anti-PD-1 | 98 | 20 | 78 |
| phs000452 | Melanoma | anti-PD-1 | 153 | 63 | 90 |

**Supplementary Table 7.** Hyperparameter settings for the four machine learning algorithms.

| **Algorithm** | **Method (caret)** | **Hyperparameters** | **Value** |
| --- | --- | --- | --- |
| Random Forest | rf | mtry | Optimized by 5-fold cross-validation |
|  |  | ntree | 500 |
| Gradient Boosting Machine | xgbTree | nrounds | 100 |
|  |  | max_depth | 3 |
|  |  | eta | 0.1 |
|  |  | gamma | 0 |
|  |  | colsample_bytree | 0.7 |
|  |  | min_child_weight | 1 |
|  |  | subsample | 0.7 |
| Kernel Support Vector Machine | svmRadial | C | Optimized by 5-fold cross-validation |
|  |  | sigma | Optimized by 5-fold cross-validation |
| Partial Least Squares | pls | ncomp | Optimized by 5-fold cross-validation |

**Supplementary Table 8.** Prognostic evaluation of STEM-LncCRT across 25 cancer types.

| **STEM-LncCRT** | **Number** | **Cancer type** |
| --- | --- | --- |
| ATAD5/PRR11-AS1/SKP2 | 16 | BLCA, BRCA, CHOL, COAD, GBM, HNSC, KIRC, KIRP, LGG, LIHC, LUAD, OV, READ, SARC, STAD, UCEC |
| ATAD5/PRR11-AS1/EZH2 | 15 | BLCA, BRCA, COAD, HNSC, KIRC, KIRP, LGG, LIHC, LUAD, LUSC, OV, READ, SARC, STAD, UCEC |
| ATAD5/PRR11-AS1/DHX9 | 14 | BRCA, CHOL, COAD, GBM, HNSC, KIRP, LGG, LIHC, LUAD, LUSC, OV, READ, SARC, STAD |
| ATAD5/PRR11-AS1/E2F3 | 14 | BLCA, BRCA, COAD, ESCA, HNSC, KIRC, KIRP, LGG, LIHC, LUAD, OV, READ, STAD, UCEC |
| ATAD5/PRR11-AS1/SMARCC1 | 14 | BRCA, CHOL, COAD, ESCA, GBM, HNSC, KIRP, LGG, LIHC, OV, READ, SARC, STAD, UCEC |
| ATAD5/PRR11-AS1/CPSF3 | 13 | BLCA, BRCA, CHOL, COAD, ESCA, HNSC, KIRP, LGG, LIHC, LUAD, READ, STAD, UCEC |
| ATAD5/PRR11-AS1/POU2F1 | 13 | BLCA, BRCA, CHOL, COAD, ESCA, GBM, HNSC, LGG, LIHC, LUAD, OV, SARC, STAD |
| RIF1/NORAD/E2F3 | 12 | BRCA, CHOL, COAD, ESCA, GBM, HNSC, LGG, LIHC, LUAD, SKCM, STAD, UCEC |
| MSH2/PRR11-AS1/E2F3 | 11 | BLCA, BRCA, ESCA, HNSC, LIHC, LUAD, LUSC, OV, STAD, TGCT, UCEC |
| MSH2/PRR11-AS1/EZH2 | 11 | BLCA, BRCA, HNSC, LIHC, LUAD, LUSC, OV, SARC, STAD, TGCT, UCEC |
| SFPQ/PRR11-AS1/SKP2 | 11 | ESCA, GBM, HNSC, LGG, LIHC, LUAD, OV, READ, SARC, STAD, UCEC |
